# Diverse helical structures made of achiral mesogenic dimers

**DOI:** 10.1038/s41467-026-72565-8

**Published:** 2026-05-06

**Authors:** Abigail Pearson, Ahlam Alshammari, Grant J. Strachan, Magdalena Majewska, Damian Pociecha, John M. D. Storey, Corrie T. Imrie, Nataša Vaupotič, Rebecca Walker, Ewa Gorecka

**Affiliations:** 1 Department of Chemistry, School of Natural and Computing Sciences, University of Aberdeen, Aberdeen, UK; 2 Department of Chemistry, College of Science, University of Ha’il, Ha’il, Saudi Arabia; 3 University of Warsaw, Faculty of Chemistry, ul. Zwirki i Wigury 101, Warsaw, Poland; 4 Department of Physics, Faculty of Natural Sciences and Mathematics, University of Maribor, Koroška 160, Maribor, Slovenia; 5 Jozef Stefan Institute, Jamova 39, Ljubljana, Slovenia

**Keywords:** Liquid crystals, Liquid crystals

## Abstract

The formation of chiral structures from achiral building blocks, and the propagation of chirality across length-scales in soft matter are still poorly understood phenomena. Here, we have studied a system revealing an exceptional diversity of spontaneously chiral phases formed by achiral mesogenic dimers linked by spacers with an odd number of atoms. Depending on terminal chain length and temperature, these compounds form a well-known heliconical nematic (N_TB_) phase and distinct helical smectic phases - ranging from the nanoscale-pitch SmC_TB–SH_, through the SmC_TB–DH_ phase with ~ 50 nm periodicity, to the newly identified SmC_TB–C_ phase featuring a micron-scale helix. In the SmC_TB–C_ phase heliconical order is preserved even though the interlayer molecular intercalations strongly suppress the azimuthal rotation of the director. Resonant soft X-ray scattering (RSoXS) measurements were performed for the first time across an entire homologous series. Results confirmed the double-helical structure of the SmC_TB–DH_ phase in which a longer helix is superimposed on the short one. The intensity of the resonant signals revealed an anomaly: the non-monotonic temperature evolution is due to the transient passage of the structure from a four-layer helix, through a nearly perfect three-layer clock-like helix, before decoupling of the short and long helices at lower temperatures.

## Abstract (web summary)

The formation of chiral structures from achiral building blocks, and the propagation of chirality across length-scales is poorly understood. Here, the authors report the dependency of the terminal chain length and temperature on the formation of diverse chiral phases from achiral mesogenic dimers linked by spacers with an odd number of atoms.

## Introduction

The spontaneous breaking of mirror symmetry is a widespread phenomenon that manifests across a broad spectrum of materials. In crystallography, structural chirality may arise despite the absence of intrinsically chiral building blocks: of the 230 crystallographic space groups, 65 are enantiomorphic and permit the presence of screw-axes. Classical examples of chiral structures made of achiral building blocks include α-quartz^1^, cinnabar (HgS)^2^, and sodium chlorate (NaClO₃)^3^, where assemblies of achiral tetrahedra, octahedra, or simple ions give rise to macroscopic helical lattices. Stable helical conformations are also found among many synthetic polymers composed of achiral monomeric units. Notable cases include poly(phenylacetylene)^4^ and polysilanes^5^, where steric constraints enforce a preferred helical geometry along the polymer backbone. In these systems, helicity persists over long chain lengths, leading to measurable optical activity of the polymer despite the absence of molecular chirality at the monomer level. Spontaneous mirror-symmetry breaking may also be observed in nanoparticle assemblies, e.g. suspensions of achiral rods or platelets sometimes organise into twisted superstructures^6^. Such symmetry breaking usually leads to coexistence of left- and right-handed domains but might also end in a homochiral state. The most striking examples of chiral amplification are the Soai asymmetric autocatalysis reaction^7^ and Viedma ripening^8^; in these processes the system undergoes spontaneous symmetry breaking, converting an initially racemic state into a homochiral one.

**Fig. 1:**
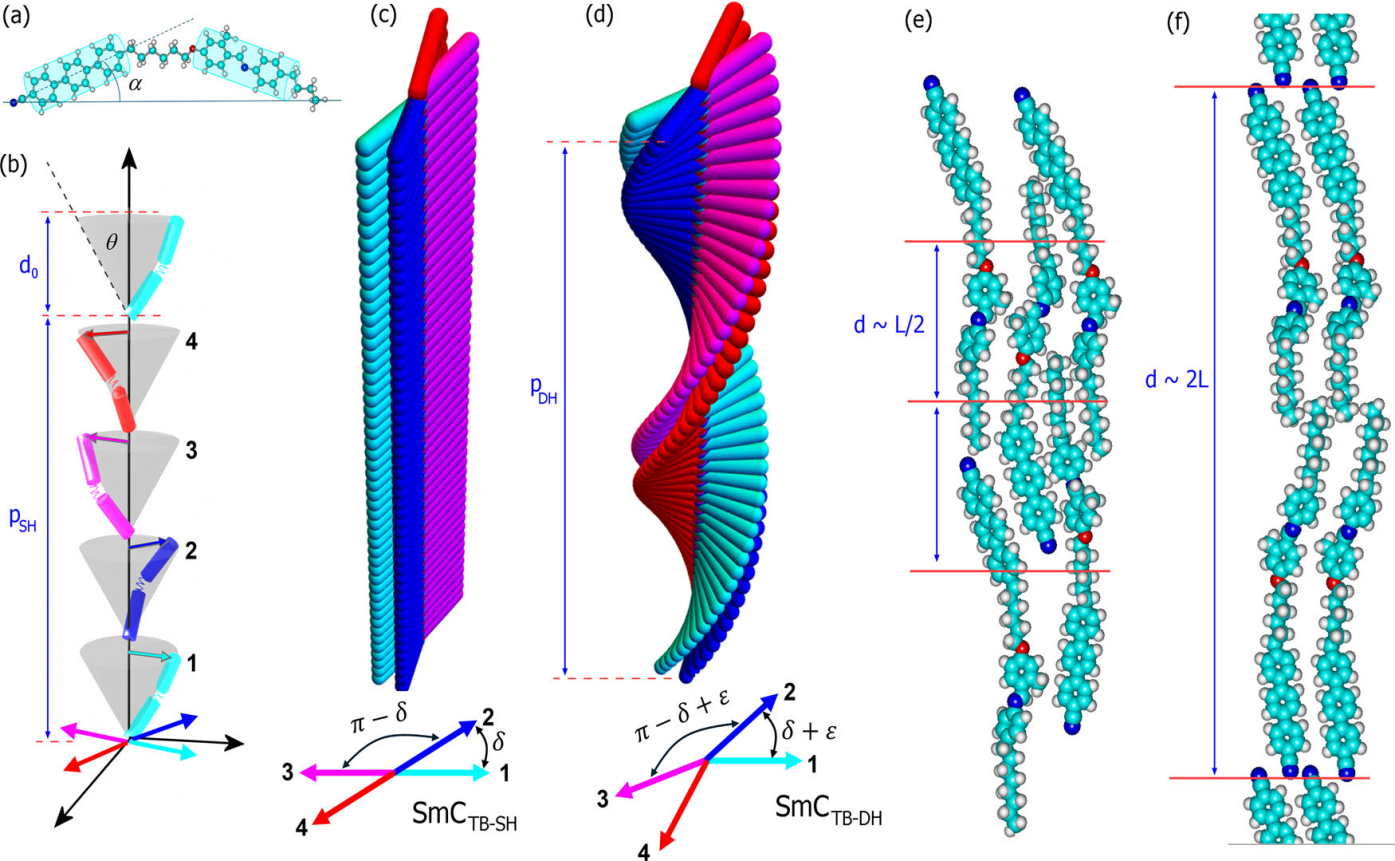
Heliconical smectic phases of bent dimers. **a** Bending of dimeric molecule is defined by an angle α between the direction of a single mesogenic core and the molecular long axis, **b** In SmC_TB_-type phases, molecules are tilted by an angle θ with respect to layer normal, and their azimuthal position on the tilt cone changes with a basic 4-layer periodicity (single layer has thickness d_o_), defining the pitch of a primary helix, p_SH_, arrows indicate projection of molecules in consecutive layers on the xy plane. **c** Distorted clock-like structure of the SmC_TB-SH_ phase (only projections of molecules onto smectic planes are shown for clarity), in which the azimuthal position of molecules on the tilt cone changes by an angle δ and π−δ in consecutive layers. **d** Double helical structure of SmC_TB-DH_, in which a longer helix (with pitch length p_DH_) is superimposed on the short, four-layer one. The secondary helix is obtained by an additional azimuthal rotation of the director (by an angle ε) when going from layer to layer. Schemes of **e** intercalated and **f** bilayer molecular packing modes realised for short and long homologues, respectively.

The emergence of spontaneous helicity extends also to liquid crystals, however the lack of positional correlation between molecules makes it less frequent. For decades, it was believed that fluids composed of achiral molecules could not exhibit chirality but recently, the breaking of mirror symmetry was reported even in an isotropic phase made of some small aggregates of mesogenic molecules^9^. The first observation of chiral liquid crystalline phases made of achiral bent-core mesogens came with the report of layer chirality arising from the combined effects of molecular tilt and polar order^10^. A further breakthrough was the identification of the twist-bend nematic phase (N_TB_), the first example of a heliconical liquid structure formed spontaneously from achiral molecules^11–13^. In the N_TB_ phase molecules are tilted at an oblique angle relative to the helical axis and gradually change the azimuthal direction, forming short helices (usually on the order of 10 nm) of both handedness with equal likelihood. The range of liquid crystalline structures showing spontaneous helicity has since expanded. Dozov’s prediction of heliconical smectics^11^ was realised in the discovery of heliconical twist-bend smectic phases (SmC_TB_) in dimeric mesogens^14–16^. Structures reported to date include not only those with simple heliconical modulations with clock-like azimuthal rotations of the director between successive layers (SmC_TB-α_) predicted by Dozov, but also phases with a distorted clock-like structure (twist-bend smectic single helix, SmC_TB-SH_) and more complex super-helical variants (twist-bend smectic double helix, SmC_TB-DH_), in which a secondary helix is superimposed upon the primary one (Fig. 1)^17,18^. This exemplifies a complex organisation in which simpler helical units assemble into progressively longer helical superstructures. Such hierarchical chiral ordering appears to be relatively rare in nature for achiral building blocks. In the liquid crystal field, an example is the B_4_ phase: this lamellar, soft-crystal-like structure forms twisted filaments, which may further assemble into twisted aggregates due to geometric packing effects^19^.

The structure of the SmC_TB_-type phases is primarily governed by elastic and steric interactions. In systems composed of bent molecules, a negative bend elastic constant^20^ favours the formation of a twist-bend structure with the uniform precession of the director on the tilt cone between adjacent layers. However, steric and entropic effects act in the opposite direction. Molecules tend to remain in the same tilt plane in neighbouring layers because this arrangement facilitates diffusion of molecules between layers, maximising the entropy of the system. As a result of this competition, a distorted clock-like structure forms, in which the azimuthal angle describing molecular position on the tilt cone changes between neighbouring layers in a non-uniform manner, alternating: δ and 180 −  δ when moving from layer to layer (Fig. 1). The sequence SmC_TB–SH_-SmC_TB-DH_ may be understood as a gradual evolution from a tilted smectic structure with alternating synclinic and anticlinic interfaces (4-layer periodic unit cell) toward a more stable anticlinic smectic phase at lower temperature^17^. For bent-core molecules the anticlinic arrangement is generally energetically favoured over the synclinic one^21^. A similar phenomenon is known for chiral rod-like liquid crystal systems, where the anticlinic SmC_A_ phase appears at temperatures below the synclinic SmC_S_ phase. In rod-like systems, however, the transition between synclinic and anticlinic order is discontinuous and may involve intermediate commensurate phases, with four-layer (SmC_FI2_) or three-layer (SmC_FI1_) periodicity units^22–24^. In contrast, for bent-shaped dimers the evolution toward anticlinic order seems to be continuous. The SmC_TB-DH_ phase forms a double-helix structure with a secondary helix pitch that depends on temperature^17^. The extreme cases of this system correspond to a four-layer arrangement with alternating synclinic and anticlinic interfaces and a two-layer anticlinic structure. The transition between these extremes can be obtained by a continuous change of the angle ε, which defines the secondary helix (Fig. 1), from 0° to 180°. Therefore, the transformation between the four-layer and bilayer structures may occur gradually through a progressive winding of the secondary helix on cooling.

**Fig. 2:**
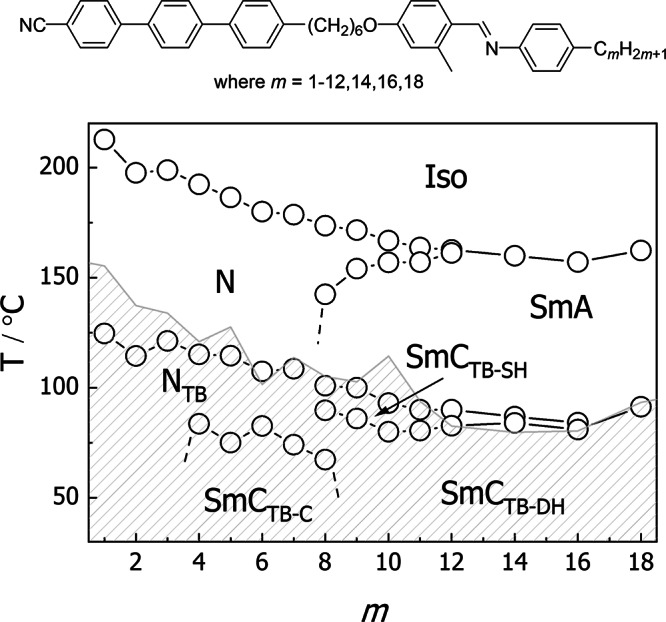
Molecular structure and a phase diagram for the CT6O2Me.*m* series. Grey striped area marks the range of monotropic LC behaviour. The N_TB_, SmC_TB-SH_, SmC_TB-DH_ and SmC_TB-C_ phases are heliconical phases.

To confirm this hypothesis we have designed a new series of dimers, referred to as CT6O2Me.*m*, with an odd number of atoms in the spacer linking two different mesogenic cores, and a terminal alkyl chain, the length of which is systematically varied with $m=1-18$ (Fig. 2). The mesogenic units are rod-like: a cyanoterphenyl unit and a benzylideneaniline group with a lateral methyl substituent (*ortho* to the imine C atom). Introducing a lateral group into the molecular structure of the dimers lowered the isotropisation temperature (~50 K), as compared to the previously studied analogues, CT6O.*m*, without a lateral substituent^25^. Lowering the shape anisotropy of the molecular arm also reduced the tendency toward the formation of lamellar structures, as all the previously studied non-substituted homologues CT6O.1–CT6O.10 exhibited a strong stability of smectic phases. CT6O2Me.*m* homologues show a much wider temperature range of nematic phases, including both the conventional nematic and the twist–bend nematic phase. These materials also form three variants of heliconical smectic phases, including a new intercalated smectic phase that appears below the N_TB_ phase. The structures of these helical phases were resolved using resonant X-ray diffraction, representing the first systematic study of an entire homologous series of dimers carried out with this method. Resonant X-ray scattering, which is sensitive not only to the electron density distribution but also to molecular orientation, has proven to be a very powerful technique for investigating helical structures. This method was crucial for resolving the structures of the SmC* subphases formed by rod-like molecules^22,23,26^ and  bent-core molecules^27,28^, the N_TB_ phase^29^, and the SmC_TB_ phases^17,18^.

## Results

The relationship between phase behaviour and the length of the terminal alkyl chain, $m$, is illustrated in Fig. 2 and thermal data given in the SI (Supplementary Table 2). Shorter homologues, where $m < 8$, form conventional and twist-bend nematic phases. The local structure of these phases is intercalated, the basic periodicity along the director is defined by a single mesogenic core length. Homologues with $m=4-7$ also exhibit an intercalated twist-bend smectic phase (with layer spacing $d \sim L/2$, where $L$ is molecular length, see Fig. 1e). We have named this phase SmC_TB-C_, where c = intercalated reflects historical nomenclature for liquid crystal dimers exhibiting intercalated (SmX_c_) and interdigitated (SmX_d_) smectic variants (X = A, C)^30^, and the structure of this phase will be the focus of subsequent discussion. The intercalated structure is driven by steric effects – the length of the terminal chain being comparable to the length of the internal spacer allows for efficient mixing of terminal chains and internal spacers in alkyl sublayers, dividing regions of aromatic cores^31^. For the dimers studied here, this results in an island of stability for intercalated smectic phases in homologues $m=4-8$.

Longer homologues, $m > 8$, below the conventional nematic phase form a smectic A phase with layer spacing $d \sim 2L$, where the basic structural unit is bilayer due to the antiparallel orientation of dimers in consecutive layers (see Fig. 1f). Upon cooling the SmA phase, tilted heliconical smectic phases, SmC_TB-SH_ and SmC_TB-DH_, are observed. With increasing terminal chain length, the range of the SmC_TB-SH_ phase narrows and for the homologue with $m=18$ a direct transition from the SmA phase to the SmC_TB-DH_ phase is found. The most interesting phase sequence is observed for the homologue with $m=\,8$, which has four smectic phases below the N phase, three of them having bilayer-type structures with $d \sim 2L$: the SmA, SmC_TB-SH_ and SmC_TB-DH_ phases, and the fourth, the SmC_TB-C_ phase, exhibiting an intercalated structure with $d \sim L/2$.

The mesophases were identified primarily using optical and X-ray diffraction (XRD) techniques. Characteristic textures were observed using the polarised optical microscope (POM): the nematic schlieren texture, the striped texture of the N_TB_ phase, and focal conic fans with regions of homeotropic alignment typical of the SmA phase (Supplementary Fig. 4). At the SmA-SmC_TB-SH_ phase transition a schlieren texture with stripes emerged from homeotropic regions, and these birefringent regions once again became homeotropic in the SmC_TB-DH_ phase (Supplementary Fig. 4). These observations are consistent with earlier reports^17,18^. The XRD experiments supported these assignments: the N and N_TB_ phases, with short-range positional ordering, show only weak, diffuse signals in both small- and wide-angle regions; in all smectic phases, the sharp small-angle signal corresponds to the layer spacing $d$, while the wide-angle signal remains diffuse, indicative of liquid-like ordering within the smectic layers.

**Fig. 3:**
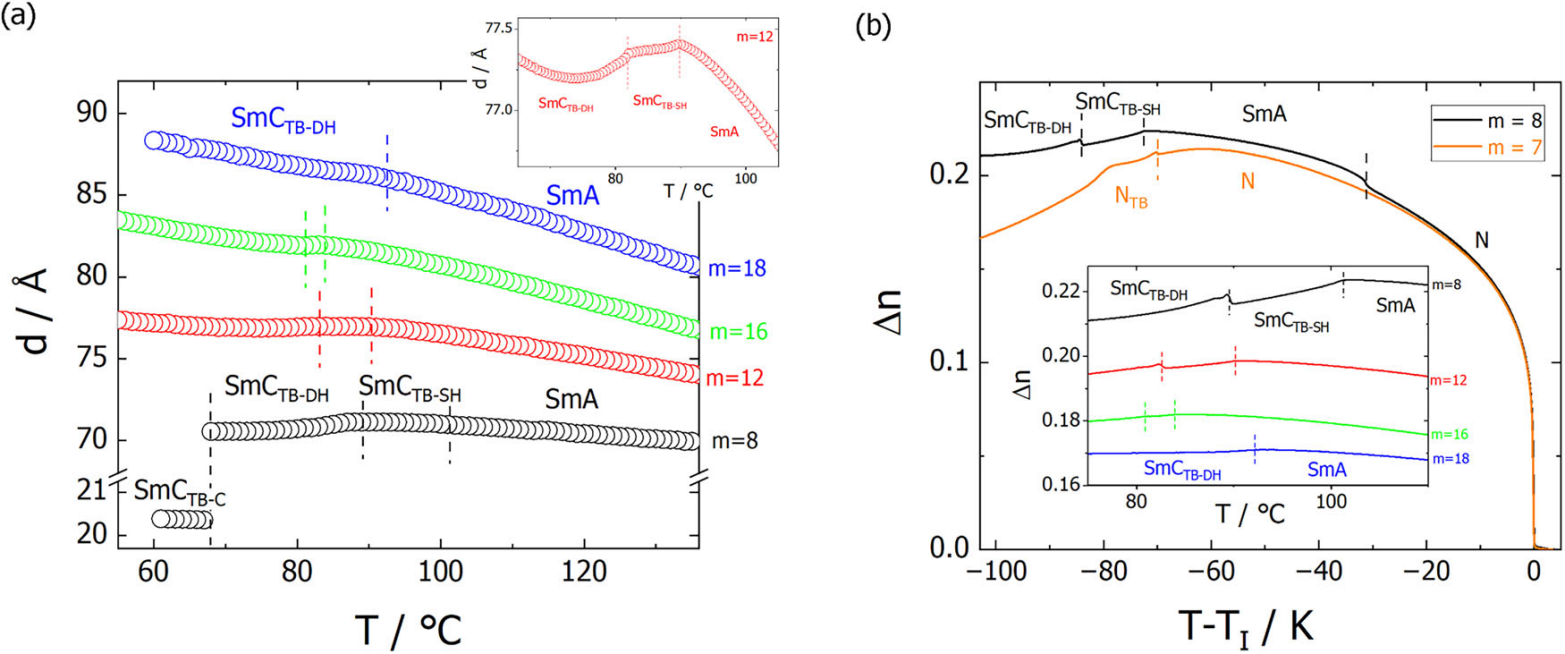
Smectic layer thickness and optical birefringence vs. temperature. **a** The layer spacing, $d$, shows a strong increase on cooling due to conformational changes, which partially screens the decrease of layer thickness caused by tilting of molecules in the SmC_TB_-type phases. Blue circles show layer spacing for homologue *m* = 18, green circles *m* = 16, red circles *m* = 12 and black circles *m* = 8. In the inset: an enlarged part of the $d$ vs. ${T}$ dependence for the homologue with $m=12$. **b** The birefringence, $\varDelta n$, in the N phase follows a critical increase of orientational order, while the small increase of $\varDelta n$ in the SmA phase is due to the coupling of orientational order to positional order in the smectic phase. The decrease of $\varDelta n$ in N_TB_ and SmC_TB_ phases is due to the helix formation. The black line shows the change in optical birefringence with temperature for homologue *m* = 8, the orange line *m* = 7. Inset colours are as for (**a**).

The $d$-spacings of the SmA, SmC_TB-SH_, and SmC_TB-DH_ phases (Fig. 3a) were found to be approximately twice the molecular length, $L$, consistent with a bilayer arrangement (Fig. 1f). All compounds exhibit strong negative thermal expansion of the layers in smectic phases, with the expansion coefficient increasing with elongation of the terminal chain (from 0.03 Å K⁻¹ to 0.13 Å K⁻¹ for $m=8$ and $m=18$, respectively), likely due to a higher probability of non–all-*trans* conformations for longer chains. In smectic C–type phases, the expected decrease of the layer spacing due to molecular tilt is partially compensated by an increase in the layer spacing caused by conformational changes. This compensation is more pronounced for longer homologues, and as a result the step down in layer spacing associated with the formation of tilted SmC-type phases becomes progressively smaller as the terminal chain length increases. In the SmC_TB-C_ phase, the layer spacing corresponds to roughly half the molecular length. Optical birefringence measurements showed typical behaviour at the transition from the N phase to the SmA phase (Fig. 3b and Supplementary Fig. 5): $\Delta n$ slightly increases as layer formation enhances the orientational order of molecules^32^. Conversely, in the N_TB_ phase, $\Delta n$ decreases because the formation of the heliconical structure reduces the refractive index along the helical axis while increasing that perpendicular to the helix^33^. A similar trend is observed at the transition from the SmA phase to heliconical smectic phases: molecular tilting leads to a decrease in $\Delta n$; a small step up at the SmC_TB-SH_ to SmC_TB-DH_ phase transition is due to a slight increase in orientational order. It was also observed that the decrease in the optical birefringence due to the tilt in the SmC_TB_ phases is less pronounced for longer homologues, consistent with the observed changes in layer thickness. Based on the decrease of $\Delta n$ in the smectic C phases, it could be estimated that tilt angle increases critically on cooling, approaching 20 deg (Supplementary Fig. 5).

**Fig. 4:**
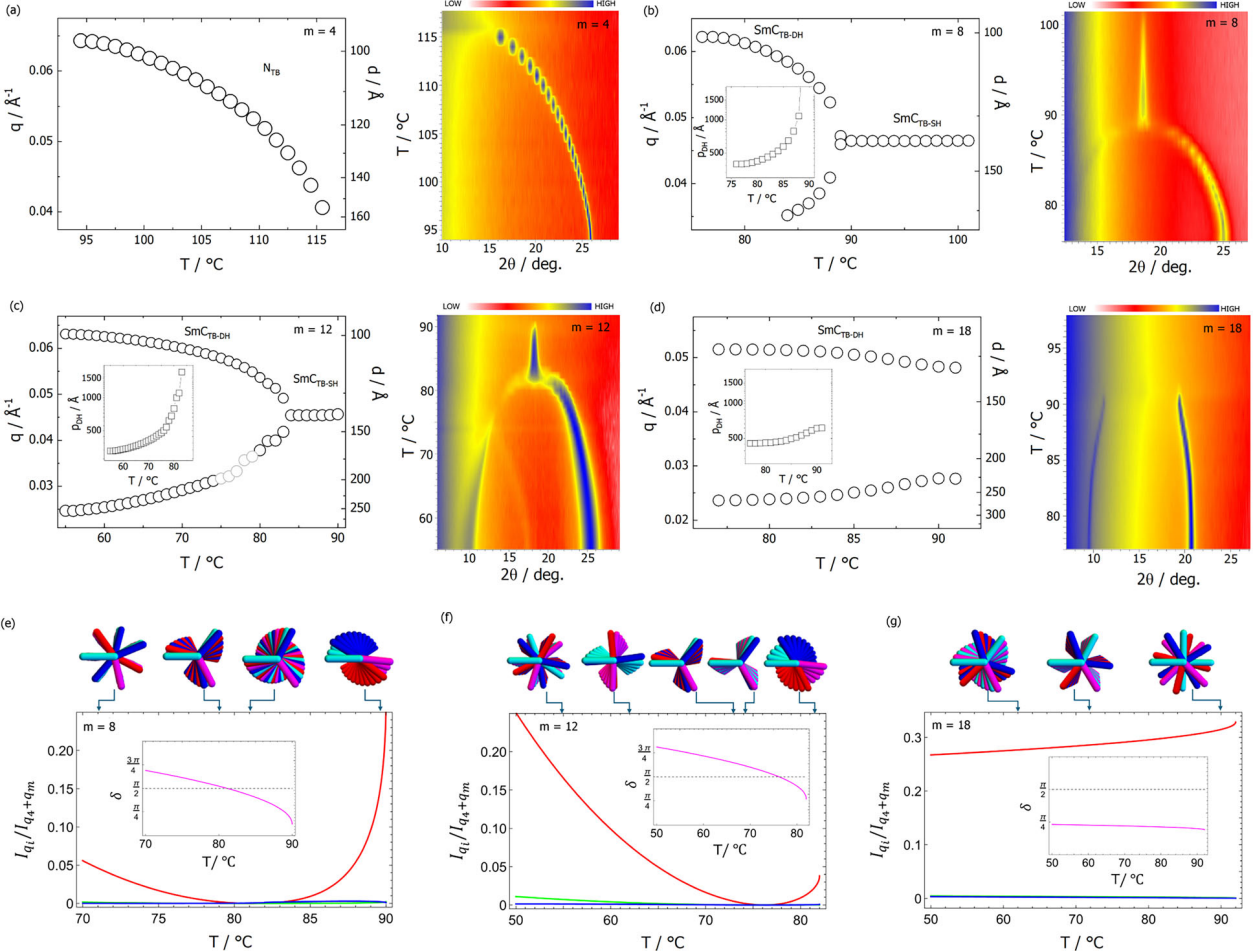
RSoXS data for homologues (a) m=4, (b) m=8 (c) m=12 and (d) m=18. Colour images show evolution of resonant diffraction signals with temperature; the angular position ($2\theta$) of signals was recalculated to the magnitude of the scattering vector $q$ and the corresponding periodicity, $d$. In the N_TB_ phase (homologue with $m=4$) the resonant signal appears due to the helix formation, and the helix unwinds on approaching the N phase. In the SmC_TB-SH_ phase (homologues with $m=8$ and $m=12$) the signal at ${q}_{4}=2\pi /(4{d}_{0})$, where ${d}_{0}$ is a single molecular layer thickness, corresponds to a four layer structure; in the SmC_TB-DH_ symmetric splitting of the signal to ${q}_{4}-{q}_{m}$ and ${q}_{4}+{q}_{m}$ is due to the additional modulation superimposed on the four layer structure, where ${q}_{m}$ is the scattering vector magnitude due to the superimposed helix (with a pitch ${p}_{{DH}}$, the temperature evolution of which is given in the insets in (**b–d**)). The homologue with $m=18$ shows a direct first order transition from the SmA to the SmC_TB-DH_ phase. **e–g** Temperature dependence of the intensity ratios between the peaks at ${q}_{i}={q}_{4}-{q}_{m}$ (red curves), ${q}_{i}=2{q}_{m}$ (green curves), ${q}_{i}={q}_{2}-2{q}_{m}$ (blue curves) and the peak at ${q}_{4}+{q}_{m}$ for (**e**) ${m}=8$, (**f**)$\,m=12$ and (**g**) $m=18$. The insets give the modelled temperature variation of the angle $\delta$. Top view on the helical structure is given at some temperatures. Model parameters are given in the main text and Supplementary Table 1.

To resolve the structure of the heliconical phases, resonant soft X-ray scattering (RSoXS) measurements were performed for selected homologues showing N_TB_, SmC_TB-SH_ and SmC_TB-DH_ phases (Fig. 4 and Supplementary Fig. 6). Resonant X-ray scattering relies on the energy dependence of the atomic scattering factor^26^. When the incident photon energy is tuned to the absorption edge of an element present in the molecular structure the atomic form factor acquires an anisotropic tensorial contribution, which couples to the direction of chemical bonds and thus provides information about molecular orientation. By analysing the position of the resonant diffraction signal for the homologue with ${m}\,=\,4$ in the N_TB_ phase it was found that the length of the helical pitch is approximately three to four molecular lengths and on heating it exhibits a critical unwinding when approaching the conventional nematic phase. In the SmC_TB-SH_ phase, formed by longer homologues, the detected resonant signal corresponds to four molecular lengths, which is twice the periodicity found by non-resonant XRD. This periodicity is very weakly temperature dependent, it exhibits only a small layer expansion with decreasing temperature, which is also detected by non-resonant XRD. In the RSoXS pattern the resonant signal is at ${q}_{4}=2\pi /{p}_{{SH}}$, where ${p}_{{SH}}=4{d}_{0}$ is a helical pitch and ${d}_{0}$ is the thickness of a single molecular layer, suggesting a short helix made of four molecules. The optical biaxiality detected for light propagating along the layer normal indicates that the helix is distorted from an ideal clock-like structure, i.e. in consecutive layers the director rotates on the tilt cone by an azimuthal angle $\delta \ne 90$° (Fig. 1). On cooling into the SmC_TB-DH_ phase, formation of a secondary helix (with a helical pitch ${p}_{{DH}}$, incommensurate with the four-layer primary helix) causes the RSoXS signal to split into ${q}_{4}-{q}_{m}$ and ${q}_{4}+{q}_{m}$ (with ${q}_{m}=2\pi /{p}_{{DH}}$). The secondary helix results from an additional azimuthal rotation, by angle $\varepsilon=2\pi {d}_{0}/{p}_{{DH}}$, superimposed on the rotation of the director in the four-layer unit (defined by angle $\delta$). The splitting between the resonant signals increases on cooling, indicating a shortening of the secondary helix with decreasing temperature. The non-equal intensities of the signals at ${q}_{4}-{q}_{m}$ and ${q}_{4}+{q}_{m}$ suggest that the basic four-layer helical unit remains of a distorted–clock type in the SmC_TB-DH_ phase. For the homologue with $m=8$ the intensity ratio of the split signals, ${I}_{{q}_{4}-{q}_{m}}/{I}_{{q}_{4}+{q}_{m}}$, is $\sim 0.2$ close to the transition and decreases on cooling (Supplementary Fig. 6). The pitch of the secondary helix quickly decreases with decreasing temperature, to ~50 nm, moving the ${q}_{4}-{q}_{m}$ branch out of the angle range accessible in RSoXS measurements. The longest studied homologue, $m=18$, shows a strongly first order direct SmA-SmC_TB-DH_ transition, that is marked by the sudden appearance of two resonant signals related to the double helical structure. For this homologue the ${I}_{{q}_{4}-{q}_{m}}/{I}_{{q}_{4}+{q}_{m}}$ intensity ratio is $\sim 0.3$, and the pitch of the secondary helix (${p}_{{DH}} \sim 50\,{{\rm{nm}}}$) is only weakly temperature dependent. A non-resonant signal at ${q}_{2}=2\pi /(2{d}_{0})$, related to the bilayer structure, is also detected in the accessible angle range of the RSoXS experiment, but, interestingly, no split signals (expected at ${q}_{2}-2{q}_{m}$ and ${q}_{2}+2{q}_{m}$) were detected around this signal, and neither was the signal at $q=2{q}_{m}$. The most interesting results were obtained for the homologue with $m=12$. In this compound the intensity ratio ${I}_{{q}_{4}-{q}_{m}}/{I}_{{q}_{4}+{q}_{m}}$ does not change monotonically with temperature; a few K below the transition to the SmC_TB-DH_ phase, the intensity of the lower-angle split signal diminishes to approximately zero and on further cooling the signal reappears (Supplementary Fig.6). Additionally, a weak signal at $2{q}_{m}$ is observed.

For the homologue with $m=18$, the intensity ratio ${I}_{{q}_{4}-{q}_{m}}/{I}_{{q}_{4}+{q}_{m}} \approx 0.3$ is higher than that for the homologue with $m=12$, and it slightly decreases with decreasing temperature.

**Fig. 5:**
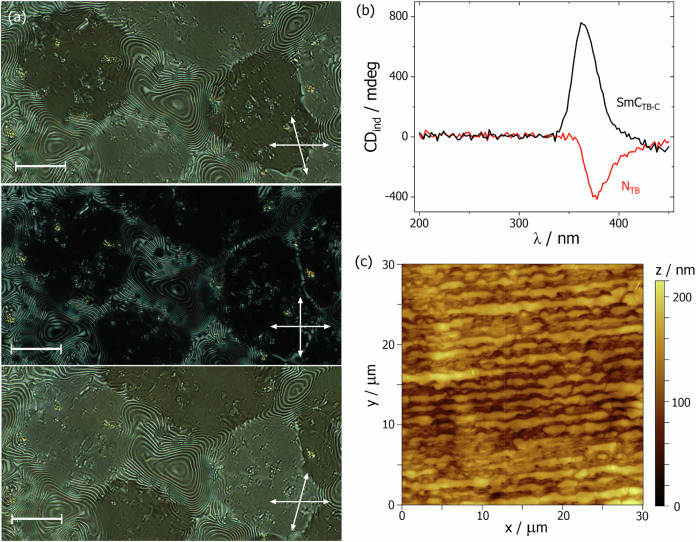
Intercalated helical twist-bend smectic phase SmC_TB-C._ **a** Optical texture for the homologue with $m=8$ observed between crossed and slightly uncrossed polarizers; de-crossing of polarizers reveals domains with the opposite optical rotatory power. Between these domains a dense schlieren texture is formed. Scale bar corresponds to 50 μm. **b** CD signals recorded in the SmC_TB-C_ and N_TB_ phases for the homologue with $m=4$. **c** The AFM image of the surface in the SmC_TB-C_ phase at room temperature for the homologue with $m=8$.

We now turn our attention to the SmC_TB-C_ phase, observed in homologues with $m=4-8$. The layer spacing $d\sim L/2$, indicates an intercalated molecular arrangement with each smectic layer composed of mixed cyanoterphenyl and benzylideneaniline mesogenic cores, separated by alkyl sublayers made of the linking and terminal chains. The SmC_TB-C_ phase exhibits focal conic fan textures when observed in cells with planar anchoring, whereas in samples with a free surface - where boundary conditions favour homeotropic alignment - optically active domains on the millimetre scale appear, separated by regions of dense schlieren texture (Fig. 5a). The presence of a schlieren texture indicates optical biaxiality, but the simultaneous observation of optical activity is inconsistent with either a conventional smectic C phase (SmC) or a biaxial smectic A phase (SmA_b_). The optical activity is clearly visualised by uncrossing the polarisers (Fig. 5a) and it suggests a degree of structural chirality, namely a helical structure. The optical rotatory power (ORP) is measured to be of the order of $1-2{\ }deg/{{\rm{\mu }}}{{\rm{m}}}$, which according to the de’Vries formula^34^, suggests a micron-scale pitch, which is much longer than in the SmC_TB-DH_ phase. Unfortunately, for RSoXS performed at the carbon atom absorption edge, the small layer periodicity of this intercalated phase prevents the observation of diffraction signals due to the helix, as they are out of the accessible angle range.

The proposed helical structure of the SmC_TB-C_ phase was further probed through measurements of circular dichroism (CD). Synchrotron CD measurements of the homologue with $m=4$ in the temperature range of its smectic phase gave a single, clear signal at the wavelength of absorption, indicative of chirality in the system (with some imbalance of right- and left-handed helices in the tested areas) (Fig. 5b). Upon heating into the helical N_TB_ phase, the signal (recorded from the same area) inverts, suggesting a reversal in the handedness of the helix at the N_TB_-SmC_TB-C_ transition. The accompanying decrease in the intensity of CD signals is likely due to the helical pitch being much shorter in the N_TB_ phase and/or a change in the size of chiral domains (i.e. regions of a single handedness of helix), which could be smaller in the N_TB_ phase compared to the smectic phase. Observations of the SmC_TB-C_ surface morphology by atomic force microscopy (AFM) revealed a stripe pattern with a periodicity of approximately 1.5 µm (Fig. 5c), which can be taken as an estimate of the helical pitch. This result is in good agreement with the helical pitch estimated from measurements of ORP.

To investigate the cross-over between intercalated and bilayer helical smectic phases, binary mixtures of homologues with $m=7$ and $m=8$ were prepared (Supplementary Fig. 7). For concentrations below 0.28 wt.% of the material with $m=8$, the same phase sequence as in the homologue with $m=7$ was found, while for concentrations above 0.75 wt.% of the material with $m=8$, the phase sequence was similar to that of the homologue with $m=8$. In the intermediate concentration range a phase sequence with re-entrant nematic order was found: N – SmA – SmC_TB-SH_ – reN_TB_ – SmC_TB-C_. The sequence was confirmed by XRD measurements. For a temperature range of just  a few K between the bilayer and intercalated smectic phases, a diffused diffraction signal is observed, signifying the lack of long-range positional order (Supplementary Fig. 8). Optical observations confirmed that this phase is N_TB_-type. The behaviour suggests that upon strong frustration between bilayer and intercalated packing, the system favours disordered nematic-type ordering.

## Discussion

The asymmetric dimers studied in this work form a range of heliconical liquid crystal phases, with both nematic (N_TB_) and smectic order (SmC_TB-SH_, SmC_TB-DH_, and SmC_TB-C_). The pitch length in these phases ranges from a few nm (N_TB_) to over a micron (SmC_TB-C_). This is a much longer pitch length than has been reported for the other twist-bend type phases and may be rationalised by considering the packing arrangement within the smectic layers of the SmC_TB-C_ phase. The intercalation of mesogenic units will dramatically constrain the rotation of molecules between neighbouring smectic layers, producing a much longer helix than in phases with either monolayer (SmC_TB-α_) or bilayer (SmC_TB-SH/DH_) structure. The observed variation in phase structure appears to be a result of the competition between different effects governing the packing of the molecules. The phase diagram reveals two distinct structural regimes: short homologues adopt an intercalated arrangement (N_TB_ and SmC_TB-C_), while longer homologues form bilayer-type structures (SmC_TB-SH_ and SmC_TB-DH_). For the intermediate homologue, a temperature-driven transition from the bilayer structure at higher temperatures to the intercalated one at lower temperatures is observed. Interestingly, the competition between these two packing modes leads to the re-entrant appearance of a nematic (N_TB_) phase between the intercalated and bilayer smectic phases, which could be seen in mixtures of neighbouring homologues. The bent molecular geometry of the studied compounds contributes to further complexity of liquid crystal structures due to the low bending elasticity in such systems. Although the bending of the studied dimeric molecules is much lower than expected for the all-*trans* conformation of the odd-membered spacer, it appears that it renders negative values of the bend elastic constant and facilitates spontaneous deformations of the director field. Under these conditions, the uniform nematic or smectic state becomes unstable, and a modulated, heliconical structure with a finite pitch is formed instead. This mechanism is general, and when combined with smectic layering it gives rise to a variety of heliconical phases. In the simplest scenario, one might expect a clock-like helix, in which the director orientation on a tilt cone rotates uniformly from layer to layer. Molecular diffusion between adjacent layers (an entropic effect) destabilises this structure and favours tilting of molecules in a single plane^35^. This transforms the clock-like helix of the SmC_TB-α_ phase into a distorted-clock structure of the SmC_TB-SH_ phase observed for longer homologues. The helical pitch of this structure is exactly four molecular layers for all the studied materials – this result could be straightforwardly determined from the position of the RSoXS signal by comparing it to the layer thickness obtained from the non-resonant X-ray data. In the SmC_TB-DH_ phase the RSoXS signal corresponding to the 4-layer structure splits, signifying an additional periodic helical modulation superimposed on the primary four-layer structure; in general, the primary and secondary helices are incommensurate.

The analysis of resonant X-ray scattering data for modulated helical structures formed by rod-like molecules is relatively straightforward^26^, but in the case of dimers, the analysis is more complex due to their bent molecular geometry^17,27^. The signal intensities are influenced not only by the position of the director on the tilt cone in consecutive layers (defined by angles $\delta$), but they also depend significantly on the molecular bending, i.e. the angle between the mesogenic cores (defined as $\pi -2\alpha$, where $\alpha$ is a deviation of the mesogenic core axis from the molecular long axis; see Fig. 1a and Supplementary Fig. 9a). We modelled the RSoXS response in the SmC_TB-DH_ phase for materials with $m=8,\,12$ and $18$ (parameters are given in Supplementary Table 3). The temperature dependence of $\theta$ was deduced from the measurements of optical birefringence and of angle $\varepsilon$ from the splitting of the ${q}_{4}$ resonant signal, considering the relation ${q}_{m}=\varepsilon /{d}_{0}$, where the layer thickness ${d}_{0}$ is obtained from the relation ${q}_{4}=2\pi /(4{d}_{0})$. Small variations of the layer thickness with temperature were neglected. Close to the phase transition temperature from the single helix to the double helix phase, experimental values of $\theta$ and $\varepsilon$ follow approximately a square root dependence on temperature, so we chose: $\varepsilon={\varepsilon }_{0}+{k}_{\varepsilon }\sqrt{{T}_{0}-T}$ and $\theta={\theta }_{0}+{k}_{\theta }\sqrt{{T}_{0}-T}$, where parameters ${\varepsilon }_{0}$, ${k}_{\varepsilon }$, ${T}_{0}$, ${\theta }_{0}$ and ${k}_{\theta }$ were found by fitting to the measured values. The angle $\delta$ should increase with decreasing temperature, because from previous studies^17^ we know that ${{\rm{\delta }}}\approx \pi /2$ at the temperature at which the peak at ${q}_{4}-{q}_{m}$ disappears. Having no other argument for the explicit temperature dependence of the angle $\delta$, we used a square root dependence: ${{\rm{\delta }}}={{{\rm{\delta }}}}_{0}+{k}_{\delta }\sqrt{{T}_{0}-T}$, and then searched for values of ${\delta }_{0}$, ${k}_{\delta }$ and angle $\alpha$ that capture the main features of the measured intensity ratios for a given homologue. We checked that the choice of mathematical function for the temperature dependence of $\delta$ is irrelevant, as long as the temperature trend is the same.

First, we present results for the homologue with $m=12$, due to the unusual intensity changes of its resonant peak. For this homologue we find: ${\varepsilon }_{0}=0$, ${k}_{\varepsilon }=0.18\,{{{\rm{K}}}}^{-0.5}$, ${k}_{\theta }=0.023\,{{{\rm{K}}}}^{-0.5}$, ${\theta }_{0}=0.13$ and T_0_ = 82 °C. We searched for parameter values ${\delta }_{0}$, ${k}_{\delta }$ and $\alpha$ that yield the intensity of the $2{q}_{m}$ peak (observed in the experiment) higher than that of the ${q}_{2}-2{q}_{m}$ peak (not observed). This condition is satisfied only within a very narrow range of $\alpha$ values; $\alpha$ must be close to zero and the apex of the dimer must point in the direction opposite to the helix rotation. The graphs shown in Fig. 4f were obtained with ${\delta }_{0}=1.0$, ${k}_{\delta }=0.30\,{{{\rm{K}}}}^{-0.5}$ and $\alpha=-0.10$ (the effect of variation of these parameters is shown in Supplementary Fig. 11). The negative sign of $\alpha$ denotes the tip of the dimer being oriented in the opposite direction of the helix rotation. Such a low bending angle is consistent with the previous results for dimers with odd-numbered spacers^30^. As shown by the top view on the helical structure in Fig. 4f, in the temperature range in which ${I}_{{q}_{4}-{q}_{m}}$ approaches 0, the double-helical structure reduces to almost a perfect three-layer clock structure, with the angles $\delta \approx \pi /2$ and $\varepsilon \approx 0.5$. Thus, the molecular arrangement evolves from a four-layer structure in the SmC_TB-SH_ phase, through a three-layer structure in the SmC_TB-DH_ phase a few degrees below the phase transition, to a multiple-layer structure deep in the SmC_TB-DH_ phase (Fig. 4f).

For the homologue with $m=18$, we find ${\varepsilon }_{0}=0.34$, ${k}_{\varepsilon }=0.039\,{{{\rm{K}}}}^{-0.5}$, ${k}_{\theta }=0.020\,{{{\rm{K}}}}^{-0.5}$, ${\theta }_{0}=0.07$ and T_0_=92 °C. For this homologue the intensity ratio ${I}_{{q}_{4}-{q}_{m}}/{I}_{{q}_{4}+{q}_{m}}\,\approx 0.3$ is higher than that for the homologue with $m=12$, and it slightly decreases with decreasing temperature. The peaks at $2{q}_{m}$ and ${q}_{2}-2{q}_{m}$ are not observed. These results are qualitatively well reproduced (see Fig. 4f) by using the following set of parameters: ${\delta }_{0}=0.60$, ${k}_{\delta }=0.020\,{{{\rm{K}}}}^{-0.5}$ and $\alpha=0$ (see Supplementary Fig. 12 for the effect of variation of these parameters). We see that the angle $\delta$ is only very weakly temperature dependent, while $\alpha=0$ suggests that for larger $m$ the dimers adopt an almost rod-like shape.

Finally, for the homologue with $m=8$, ${\varepsilon }_{0}=0$, ${k}_{\varepsilon }=0.16\,{{{\rm{K}}}}^{-0.5}$, ${k}_{\theta }=0.023\,{{{\rm{K}}}}^{-0.5}$, ${\theta }_{0}=0.19$ and T_0_=90 °C. The peak at ${q}_{4}-{q}_{m}$ is observed only in a narrow temperature range (less than $10\,{{\rm{K}}}$) below the transition temperature. Within this range the temperature variation of the intensity ratio ${I}_{{q}_{4}-{q}_{m}}/{I}_{{q}_{4}+{q}_{m}}$ is well reproduced by ${\delta }_{0}=0.38$, ${k}_{\delta }=0.40\,{{{\rm{K}}}}^{-0.5}$ and $\alpha=-0.10$ (Fig. 4e) and the effect of variation of these parameters is shown in Supplementary Fig. 10.

Thus, by assuming a temperature dependence of angle $\delta$, which defines the basic four-layer structure, we can reproduce the main characteristics of the RSoXS spectra for the studied homologue series.

In conclusion, in this work we report the structural characterisation of different types of heliconical liquid crystal phases formed by a series of asymmetric bent dimers, including the first report of an intercalated twist-bend SmC phase (SmC_TB-C_). These heliconical phases differ in the degree of order (nematic or smectic), the packing of molecules (intercalated or bilayer), the length scale of the helical pitch (from a few nm to micron), as well as the presence of multiple helices (SmC_TB_-_DH_). The substantially longer helical pitch of this new SmC_TB-C_ phase may be rationalised by considering the packing arrangement within the smectic layers. It appears that intercalation inhibits significant azimuthal rotation of the director between consecutive smectic layers, resulting in a much longer, micron-scale pitch. In contrast, the SmC_TB-SH_ and SmC_TB-DH_ phases have a bilayer arrangement of molecules with only a small interdigitation of the terminal chains between the layers, and it appears that the development of the double helical structure provides a mechanism for the progression from a 4-layer structure towards a simple, two-layer anticlinic arrangement.

The evolution of heliconical structure in these phases was studied using resonant soft X-ray scattering (RSoXS) measurements. For the SmC_TB-DH_ structure, these revealed that both the distortion angle in the four-layer basic unit, as well as the pitch of the longer helix superimposed on it, are temperature dependent. Their simultaneous changes result in transitional states, in which the four-layer distorted-clock helix, observed at higher temperatures, gradually evolves into a nearly perfect three-, or six-layer clock-like structure with decreasing temperature. This structural evolution leads to non-monotonic changes in the intensities of resonant diffraction signals. This behaviour differs markedly from that observed in liquid crystals composed of rod-like chiral molecules, where with decreasing temperature the changes occur by a series of step-like transitions, between 4-layer (SmF_Fi2_), 3-layer (SmF_Fi1_) and bilayer (SmC_AF_) structures^36^.

Dimers—despite their relatively simple molecular architecture—show an exceptional diversity of structures and phase behaviour. It was well known that their behaviour was governed by shape (bending vs. straight structure depending on the parity of the spacer) and chemical compatibility of the connected units (intercalation vs. monolayer or bilayer lamellar structure) but it is now evident that dimers with odd-membered spacers, combining bent molecular shape with considerable flexibility, although achiral are capable of forming an exceptional variety of chiral phases. The dimers studied here broaden the range of known systems exhibiting spontaneous mirror symmetry breaking. The observed structural diversity, from a simple helix with a pitch length in the optical range in the intercalated smectic phase, through the short nanometre scale helix of SmC_TB-SH_, to the double helix of SmC_TB-DH_, highlights how even small structural modifications, such as extension of the terminal chain, can lead to different levels of hierarchical chiral ordering. This is important for understanding how structural chirality can emerge in condensed matter in the absence of molecular chirality and provides valuable insight into the design of new functional materials with unusual optical and electro-optical properties.

## Methods

### Optical studies

Phase characterisation was performed by polarised optical microscopy (POM) using a Zeiss AxioImager A2m microscope equipped with a Linkam T96 heating stage. Samples were sandwiched between glass plates, giving several μm-thick films. Selected samples were also prepared in commercial cells purchased from INSTEC or WAT, with a cell thickness ranging from 1.5 to 5 µm and treated for planar or homeotropic anchoring. Measurements of optical birefringence were performed with a set-up based on a photoelastic modulator (PEM-90, Hinds) working at a modulation frequency *f* = 50 kHz, according to procedure described fully by Kemp^37^ and in PEM-90 manual. A halogen lamp (Hamamatsu LC8), equipped with a narrow bandpass filter (532 nm), was used as a light source. The intensity of transmitted light vs. time recorded with a photodiode (FLC Electronics PIN−20) was de-convoluted with a lock-in amplifier (EG&G 7265) into 1 f and 2 f components, the  ratio of which is proportional to optical retardation, *R*, induced by the sample, $\frac{{V}_{1f}}{{V}_{2f}}=\frac{{J}_{1}(A)}{{J}_{2}(A)}\tan R$, where values of Bessel functions, ${J}_{1}$ and ${J}_{2}$ are taken for argument *A* being the PEM modulation amplitude, π/2. Knowing the sample thickness, *d*, the retardation was recalculated into optical birefringence, $\Delta n=\frac{R\lambda }{2\pi d}$. Samples with homogeneous planar alignment were prepared in cells of 1.5 µm thickness purchased from the WAT.

### Differential scanning calorimetry

Studies were performed using a Mettler-Toledo DSC1 or DSC3 instruments, fitted with an intracooler and calibrated using indium and zinc as standards. Heating and cooling rates were 10 K min^−1^, and all samples were measured under a nitrogen atmosphere. Transition temperatures and associated enthalpy changes were extracted from the second heating trace unless otherwise noted. For each sample, two aliquots were measured, and the data listed are the average of the two sets of data.

### X-ray diffraction

2D XRD patterns were obtained with the Bruker D8 GADDS system (CuKα radiation, Goebel mirror monochromator, 0.5 mm point beam collimator, VANTEC 2000 area detector), equipped with a modified Linkam heating stage. Samples were prepared as droplets on a heated surface. The temperature dependence of the smectic layer thickness was determined from the small-angle X-ray diffraction experiments performed with the Bruker D8 Discover system (CuKα line, Goebel mirror, Anton Paar DCS350 heating stage, scintillation counter) working in the reflection mode. Homeotropically aligned samples were prepared as a thin film on a silicon wafer. The small-angle X-ray diffraction (SAXS) patterns for powder samples were obtained with a Bruker Nanostar system using CuKα radiation, and patterns were collected with a VANTEC 2000 area detector. The temperature of the samples was controlled with a precision of ±0.1 K with MRI heating stage.

### Resonant soft X-ray diffraction (RSoXS)

Studies were performed at the Advanced Light Source, Lawrence Berkeley National Laboratory on the soft X-ray beam line (11.0.1.2). The energy of the incident beam was tuned to the K-edge of carbon absorption (283 eV). Samples with a thickness lower than 1 μm were placed on a transmission electron microscopy grid. The scattering intensity was recorded using the Princeton PI-MTE CCD detector.

### Circular dichroism

Measurements of the circular dichroism were performed at the B23 beamline at the Diamond Light Source using the Mueller Matrix Polarimetry (MMP) method developed by G. Siligardi^38^. Samples were prepared in cells consisting of two untreated quartz slides and CD spectra were acquired in the 200–450 nm range.

### Atomic Force Microscopy

AFM measurements were performed using a Bruker Dimension Icon Microscope working in tapping or scan assist mode and cantilevers with elastic constant of 0.4 N/m were applied.

### Structure and purity analysis

The molecular structure of all the final products and their intermediates were characterised using a combination of ^1^H and ^13^C NMR, and FTIR spectroscopies. ^1^H and ^13^C NMR spectra were recorded on a 400 MHz Bruker Avance III HD NMR spectrometer. Infra-red spectra were recorded on a Perkin Elmer Spectrum Two FTIR spectrometer with an ATR diamond cell. High-resolution mass spectrometry was carried out using a Waters XEVO G2 Q-Tof mass spectrometer operated by Dr Morag Douglas at the University of Aberdeen.

### Synthesis

The synthetic route used to prepare the CT6O2Me.*m* series is outlined in Supplementary Fig. 1^25^. The cyanoterphenyl-based mesogenic unit was formed by a Friedel-Crafts acylation of 4-bromobiphenyl and 6-bromohexanoyl chloride and subsequent ketone reduction, followed by a Suzuki-Miyaura cross-coupling reaction. A Williamson ether synthesis between CT6Br and 4-hydroxy−2-methylbenzaldehyde gave the dimeric aldehyde (CT6O2MeAH) which was combined in a series of Schiff base syntheses with the relevant 4-alkylanilines to form the final products. Full synthetic details and analytical data for all intermediates and final products are included in the Supplementary Information.

## Supplementary information

Supplementary Information

Transparent Peer Review file

## Source data

source data

## Data Availability

The DSC, X-ray, optical birefringence, RSoXS, and CD data generated in this study are provided in the Source Data file. All additional data are available from the corresponding author upon request. Source data are provided with this paper.
