# Supplementary material for: Diverse helical structures made of achiral mesogenic dimers
Source: Nat Commun. 2026 May 6;17:6090. doi: 10.1038/s41467-026-72565-8 (PMC13357721; doi:10.1038/s41467-026-72565-8)
Supplement: Supplementary file 1 — Supplementary Information [file 41467_2026_72565_MOESM1_ESM.pdf]

Supplementary Information

Diverse helical structures made of achiral mesogenic dimers

Abigail Pearson[1], Ahlam Alshammari[1], Grant J. Strachan[2], Magdalena Majewska[2], Damian Pociecha[2], John M. D. Storey[1], Corrie T. Imrie[1], Nataša Vaupotič[3,4], Rebecca Walker[1,*], Ewa Gorecka[2,*]

1. Department of Chemistry, School of Natural and Computing Sciences, University of Aberdeen, AB24 3UE Scotland, United Kingdom.
2. University of Warsaw, Faculty of Chemistry, ul. Zwirki i Wigury 101, 02-089 Warsaw, Poland
3. Department of Physics, Faculty of Natural Sciences and Mathematics, University of Maribor, Koroška 160, 2000 Maribor, Slovenia
4. Jozef Stefan Institute, Jamova 39, 1000 Ljubljana, Slovenia

[*] rebecca.walker@abdn.ac.uk, gorecka@chem.uw.edu.pl

1. Organic synthesis and analytical data

2. Additional experimental results

3. Theoretical model

1. Organic synthesis and analytical data

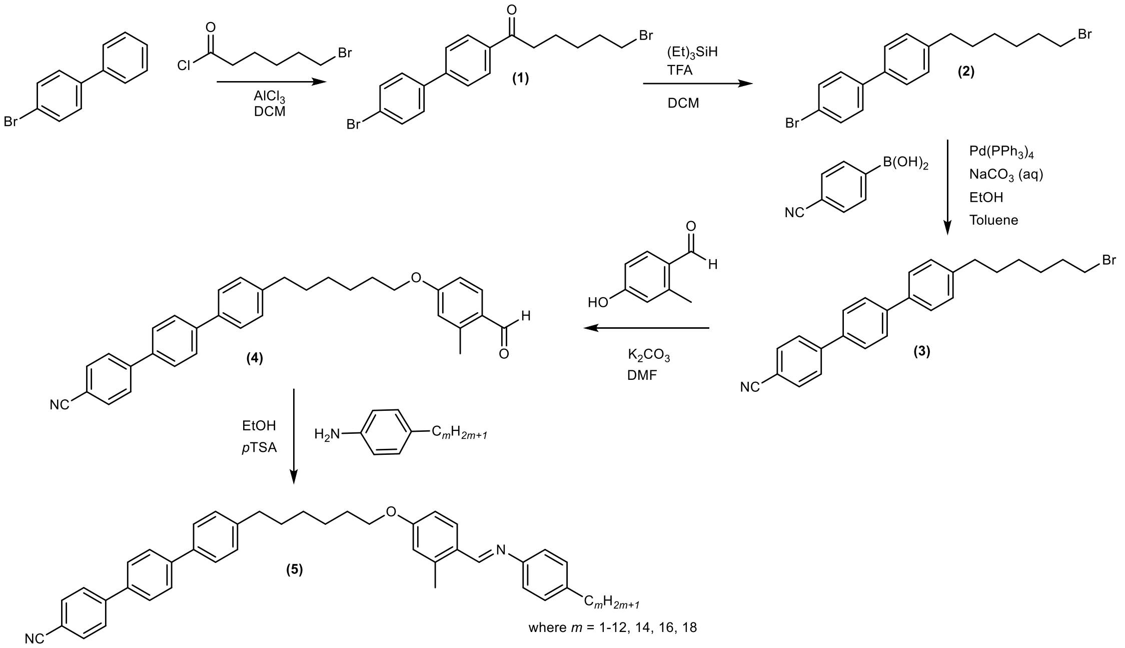

Supplementary Figure 1. Synthetic route to CT6O2Me.*m* series, where *m* = 1-12, 14, 16, 18.

(1) 6-Bromo-1-[4-(4-bromophenyl)phenyl]hexan-1-one, BrBK5Br

To a reaction vessel containing 4-bromobiphenyl (1 eq, 10.0 g, 42.9 mmol) in DCM (20 mL), $AlCl_3$ (1.1 eq, 6.29 g, 47.2 mmol) was added with stirring under argon, in an ice bath, wrapped in tinfoil to protect from light. Bromohexanoyl chloride (1.1 eq, 7.22 mL, 47.2 mmol) was added dropwise and the reaction mixture allowed to come to room temperature gradually and left to proceed. TLC (Dichloromethane, R_f 0.74) of the reaction mixture after 24 hours showed the reaction to be complete.

When the reaction was complete, the mixture was added slowly to water and extracted with DCM (3 x 100 mL). The organic layer was washed with sodium bicarbonate (1 x 100 mL) and brine (1 x 100 mL) and dried with $MgSO_4$. After filtration to remove the $MgSO_4$, the solvent was removed *in vacuo* and the crude product recrystallised from EtOH (400 mL) to give off-white crystals. Yield 13.3 g (75%).

m.p. 85 °C.

^{1}H NMR (400 MHz, $CDCl_3$) δ ppm: 8.02 (d, ^3J = 8.4 Hz, 2H, Ar-H), 7.64 (d, ^3J = 8.4 Hz, 2H, Ar-H), 7.60 (d, ^3J = 8.5 Hz, 2H, Ar-H), 7.49 (d, ^3J = 8.5 Hz, 2H, Ar-H), 3.44 (t, ^3J = 6.7 Hz, 2H, Ar-C(=O)$\underline{CH_2}CH_2CH_2CH_2CH_2Br$), 3.02 (t, ^3J = 7.3 Hz, 2H, Ar-C(=O)$CH_2CH_2CH_2CH_2\underline{CH_2}Br$), 1.93 (m, 2H, Ar-C(=O)$CH_2\underline{CH_2}CH_2CH_2CH_2Br$), 1.80 (p, ^3J = 7.3 Hz, 2H, Ar-C(=O)$CH_2CH_2CH_2\underline{CH_2}CH_2Br$), 1.56 (m, 2H, Ar-C(=O)$CH_2CH_2\underline{CH_2}CH_2CH_2Br$). ^{13}C NMR (101 MHz, $CDCl_3$) δ ppm: 199.59, 144.53, 138.92, 136.08, 132.25 (2C), 128.95 (2C), 128.87 (2C), 127.20 (2C), 122.79, 38.50, 33.77, 32.77, 28.02, 23.50. IR (vmax/cm^{-1}): 2938 (sp^2 hybridised C-H stretching), 2865 (sp^2 hybridised C-H stretching), 1909 (aromatic overtones), 1679 (ketone carbonyl stretching).

(2) 1-Bromo-4-[4-(6-bromohexyl)phenyl]benzene, BrB6Br

BrBK5Br (1 eq, 11.2 g, 27.4 mmol) and TFA (8 eq, 16.8 mL, 219.2 mmol) were combined with the minimal amount of DCM (10 mL) while stirring in an ice bath under argon. Dropwise, triethyl silane (4 eq, 17.5 mL, 109.2 mmol) was added, and the reaction allowed to come gradually to room temperature. After 6 hr, the reaction was checked by TLC (Dichloromethane R_f 0.88) and appeared to be complete.

The reaction mixture was then poured into a mixture of DCM (200 mL) and water (200 mL) and the organic layer extracted and washed with water (3 x 150 mL). The organic fraction was dried with $MgSO_4$ and after filtration to remove the $MgSO_4$, the solvent was removed *in vacuo*. The crude product was recrystallised from ethanol to give a white solid. Yield 10.66 g (98%).

m.p. 75 °C.

^{1}H NMR (400 MHz, $CDCl_3$) δ ppm: 8.02 (d, ^3J = 8.4 Hz, 2H, Ar-H), 7.64 (d, ^3J = 8.4 Hz, 2H, Ar-H), 7.60 (d, ^3J = 8.5 Hz, 2H, Ar-H), 7.49 (d, ^3J = 8.5 Hz, 2H, Ar-H), 3.44 (t, ^3J = 6.7 Hz, 2H, Ar-C(=O)$\underline{CH_2}CH_2CH_2CH_2CH_2Br$), 3.02 (t, ^3J = 7.3 Hz, 2H, Ar-C(=O)$CH_2CH_2CH_2CH_2\underline{CH_2}Br$), 1.93 (m, 2H, Ar-C(=O)$CH_2\underline{CH_2}CH_2CH_2CH_2Br$), 1.80 (p, ^3J = 7.3 Hz, 2H, Ar-C(=O)$CH_2CH_2CH_2\underline{CH_2}CH_2Br$), 1.56 (m, 2H, Ar-C(=O)$CH_2CH_2\underline{CH_2}CH_2CH_2Br$). ^{13}C NMR (101 MHz, $CDCl_3$) δ ppm: 142.32, 140.17, 137.56, 131.94 (2C), 129.09 (2C), 128.70 (2C), 126.96 (2C), 121.34, 58.63, 35.56, 34.08, 32.86,

31.33, 28.53, 28.16, 18.59. IR (νmax/cm^{-1}): 2931 (sp^2 hybridised C-H stretching), 2855 (sp^2 hybridised C-H stretching), 1903 (aromatic overtones).

(3) 4-[4-[4-(6-Bromohexyl)phenyl]phenyl]benzonitrile, CT6Br

BrB6Br (1 eq, 5.00 g, 0.013 mol), 4-cyanophenylboronic acid (1.1 eq, 2.06 g, 0.014 mol) and aqueous $NaCO_3$ (2.5 eq, 2M, 15mL) were combined in ethanol (30 mL) and toluene (20 mL) with stirring and the reaction vessel sparged with argon. Once sparged, the reaction vessel was kept under argon and $Pd(PPh_3)_4$ (0.015 eq, 0.23 g, 0.195 x10^{-3} mol) was added and the reaction heated to reflux. The reaction was left to proceed for 50 hours, at which point TLC (8:2 Dichloromethane:Petroleum Ether R_f 0.73) indicated the reaction had gone to completion.

Once completed, the reaction mixture was allowed to gradually come to room temperature. To the cooled reaction mixture, 2 M HCl was added dropwise until gas stopped being produced, then solvent was removed *in vacuo* until only water remained. Using ethyl acetate (150 mL), the product was extracted and the resulting organic layer was washed with water (3 x 150 mL) and brine (1 x 100 mL). The organic fraction was dried with $MgSO_4$, after filtration to remove the $MgSO_4$, the solvent was removed *in vacuo* to give an off-white solid. The crude product was purified by column chromatography (8:2 DCM:Petroleum Ether R_f 0.73) and the solvent removed *in vacuo*. The product was then recrystalised from ethanol (200 mL) and ethyl acetate (20 mL) to give a white powder. Yield 3.09 g (57%).

m.p. 101 °C, $T_{N\text{-}I}$ 200 °C.

^{1}H NMR (400 MHz, $CDCl_3$) δ ppm: 7.74 (m, appt s, 4H, Ar-H), 7.71 (d, ^3J = 8.6 Hz, 2H, Ar-H), 7.66 (d, ^3J = 8.6 Hz, 2H, Ar-H), 7.56 (d, ^3J = 8.3 Hz, 2H, Ar-H), 7.28 (d, ^3J = 8.3 Hz, 2H, Ar-H), 3.42 (t, ^3J = 6.8 Hz, 2H, Ar-$\underline{CH_2}CH_2CH_2CH_2CH_2CH_2$Br), 2.68 (t, ^3J = 7.6 Hz, 2H, Ar-$CH_2CH_2CH_2CH_2CH_2\underline{CH_2}$Br), 1.88 (m, 2H, Ar-$CH_2\underline{CH_2}CH_2CH_2CH_2CH_2$Br), 1.69 (m, 2H, Ar-$CH_2CH_2CH_2CH_2\underline{CH_2}CH_2$Br), 1.49 (m, 2H, Ar-$CH_2CH_2\underline{CH_2}CH_2CH_2CH_2$Br), 1.40 (m, 2H, Ar-$CH_2CH_2CH_2\underline{CH_2}CH_2CH_2$Br). ^{13}C NMR (101 MHz, $CDCl_3$) δ ppm: 145.36, 142.46, 141.63, 137.81, 137.75, 132.79 (2C), 129.13 (2C), 127.76 (2C), 127.71 (2C), 127.68 (2C), 127.10 (2C), 119.12,

111.00, 35.60, 34.09, 32.87, 31.36, 28.55, 28.17. IR (νmax/cm^{-1}): 2928 (sp^2 hybridised C-H stretching), 2855 (sp^2 hybridised C-H stretching), 2225 (nitrile stretching), 1916 (aromatic overtones).

(4) 4-[4-[4-[6-(4-Formyl-3-methylphenoxy)hexyl]phenyl]phenyl]benzonitrile, CT6O2MeAH

A mixture of CT6Br (1 eq, 1.89 g, 4.53 mmol), 2-methyl-4-hydroxybenzaldehyde (1.1 eq, 0.68 g, 4.98 mmol) and potassium carbonate (2 eq, 1.25 g, 9.06 mmol) in DMF (10 mL) was heated to 90 °C with stirring and left to proceed overnight. After 15 hr, TLC (8:2 Dichloromethane:Petroleum Ether R_f 0.33) indicated the reaction had gone to completion.

Once completed, the reaction was allowed to cool to room temperature and the reaction mixture was added to water (200 mL) resulting in the formation of a white precipitate. The solid was collected and the crude product recrystallised from ethanol (50 mL) and ethyl acetate (5 mL). Yield 1.86 g (86 %).

m.p. 116 °C, $T_{N\text{-}I}$ 178 °C.

^{1}H NMR (400 MHz, $CDCl_3$) δ ppm: 10.11 (s, 1H, Ar-C(=O)H), 7.74 (m, 5H, Ar-H), 7.70 (d, ^3J = 8.6 Hz, 2H, Ar-H), 7.66 (d, ^3J = 8.6 Hz, 2H, Ar-H), 7.56 (d, ^3J = 8.2 Hz, 2H, Ar-H), 7.29 (d, ^3J = 8.2 Hz, 2H, Ar-H), 6.82 (dd, ^3J = 8.6 Hz, ^4J = 2.5 Hz, 1H, Ar-H), 6.73 (d, ^4J = 2.5 Hz, 1H, Ar-H), 4.02 (t, ^3J = 6.4 Hz, 2H, Ar-O$\underline{CH_2}CH_2CH_2CH_2CH_2CH_2$-Ar), 2.69 (t, ^3J = 7.7 Hz, 2H, Ar-$OCH_2CH_2CH_2CH_2CH_2\underline{CH_2}$-Ar), 2.64 (s, 3H, Ar-$\underline{CH_3}$), 1.82 (m, 2H, Ar-$OCH_2\underline{CH_2}CH_2CH_2CH_2CH_2$-Ar), 1.71 (p, ^3J = 7.7 Hz, 2H, Ar-$OCH_2CH_2CH_2CH_2\underline{CH_2}CH_2$-Ar), 1.49 (m, 4H, Ar-$OCH_2CH_2\underline{CH_2}\underline{CH_2}CH_2CH_2$-Ar). ^{13}C NMR (101 MHz, $CDCl_3$) δ ppm: 191.29, 163.37, 145.33, 143.40, 142.49, 141.58, 137.81, 137.72, 134.89, 132.78 (2C), 129.13 (2C), 127.92, 127.73 (2C), 127.71 (2C), 127.67 (2C), 127.07 (2C), 119.11, 117.60, 112.03, 111.00, 68.23, 35.62, 31.43, 29.14, 29.05, 25.98, 20.03. IR (νmax/cm^{-1}): 2938 (sp^2 hybridised C-H stretching), 2857 (sp^2 hybridised C-H stretching), 2225 (nitrile stretching), 1915 (aromatic overtones), 1672 (aldehyde C=O stretching)

(5) 4″-(6-(3-Methyl-4-((alkylphenylimino)methyl)phenoxy)hexyl)-[1,1′:4,1′-terphenyl]-4-carbonitriles, CT6O2Me.*m*

Under an inert argon atmosphere, 4″-(6-(4-formyl-3-methylphenoxy)hexyl)-[1,1′:4,1″-terphenyl]-4-carbonitrile (CT6O2MeAH) (Supplementary Tab. 1) was dissolved in 15 mL ethanol and a small

amount of toluene (3 ml) at 60 °C. The appropriate 4-alkylaniline was added (Supplementary Tab. 1), followed by a crystal of *p*-toluenesulfonic acid. The reaction was then heated at 90 °C overnight. The reaction mixture was cooled to room temperature and a white precipitate formed which was collected by vacuum filtration. The crude product was recrystallised twice from hot EtOH with chloroform in varying, small amounts.

Supplementary Table 1: Quantities of reagents used in the synthesis of the CT6O2Me.*m* series.

	Quantities of CT6O2MeAH		Quantities of alkylaniline	
m	Mass / g	Moles / mmol	Mass / g	Moles / mmol
1	0.200	0.420	0.050	0.460
2	0.200	0.420	0.056	0.460
3	0.200	0.420	0.062	0.460
4	0.200	0.420	0.069	0.460
5	0.200	0.420	0.076	0.460
6	0.200	0.420	0.089	0.460
7	0.250	0.530	0.111	0.580
8	0.250	0.530	0.119	0.580
9	0.250	0.530	0.127	0.580
10	0.250	0.530	0.136	0.580
11	0.150	0.310	0.086	0.340
12	0.250	0.530	0.152	0.580
14	0.250	0.530	0.168	0.580
16	0.250	0.530	0.184	0.580
18	0.250	0.530	0.200	0.580

CT6O2Me.1

Yield: 66 %. m.p. 156 °C, (T_{NTB-N} 122 °C), T_{N-I} 212°C.

^{1}H NMR (400 MHz, $CDCl_3$) δ ppm: 8.65 (s, 1H, <u>CH</u>=N), 8.02 (d, ^{3}J = 8.7 Hz, 1H, Ar-H), 7.73 (m, appt s, 4H, Ar-H), 7.70 (d, ^{3}J = 8.4 Hz, 2H, Ar-H), 7.66 (d, ^{3}J = 8.4 Hz, 2H, Ar-H), 7.55 (d, ^{3}J = 8.1 Hz, 2H, Ar-H), 7.28 (d, ^{3}J = 8.1 Hz, 2H, Ar-H), 7.18 (d, ^{3}J = 8.2 Hz, 2H, Ar-H), 7.09 (d, ^{3}J = 8.2 Hz, 2H, Ar-H), 6.81 (dd, ^{3}J = 8.7 Hz, ^{4}J = 2.6 Hz, 1H, Ar-H), 6.72 (d, ^{4}J = 2.6 Hz, 1H, Ar-H), 4.00 (t, ^{3}J = 6.5 Hz, 2H, $ArCH_2(CH_2)_4\underline{CH_2}$-O), 2.69 (t, ^{3}J = 7.6 Hz, 2H, $Ar\underline{CH_2}(CH_2)_4CH_2$-O), 2.54 (s, 3H, $Ar\underline{CH_3}$), 2.37 (s, 3H, $Ar\underline{CH_3}$), 1.82 (m, 2H, $Ar(CH_2)_4\underline{CH_2}CH_2$-O), 1.71 (p, ^{3}J = 7.6 Hz, 2H, $ArCH_2\underline{CH_2}(CH_2)_4$-O), 1.54 (m, 2H, $Ar(CH_2)_3\underline{CH_2}CH_2CH_2$-O), 1.46 (m, 2H, $ArCH_2CH_2\underline{CH_2}(CH_2)_3$-O). ^{13}C NMR (101 MHz, $CDCl_3$) δ ppm: 161.34, 157.86, 150.59, 145.33, 142.54, 141.61, 140.65, 137.75, 137.68, 135.31, 132.76 (2C), 129.84 (2C), 129.74, 129.14 (2C), 127.74 (2C), 127.69 (2C), 127.65 (2C), 127.36, 127.06 (2C), 120.94, 119.12, 116.67, 112.59, 110.96, 67.98, 35.59, 31.42, 29.23, 29.02, 25.98, 21.11, 19.73. IR (νmax/cm^{-1}): 2918, 2850 (C-H, sp^2), 2227 (C≡N stretch), 1603 (para disubstituted benzene), 1585, 1489, 1288, 1259, 1102, 808, 518. HRMS: (TOF ESI^+) (m/z): $[M+H]^+$ Calculated for $C_{40}H_{39}N_2O$ = 562.3062; found, 563.3038 (- 4.3 PPM difference)

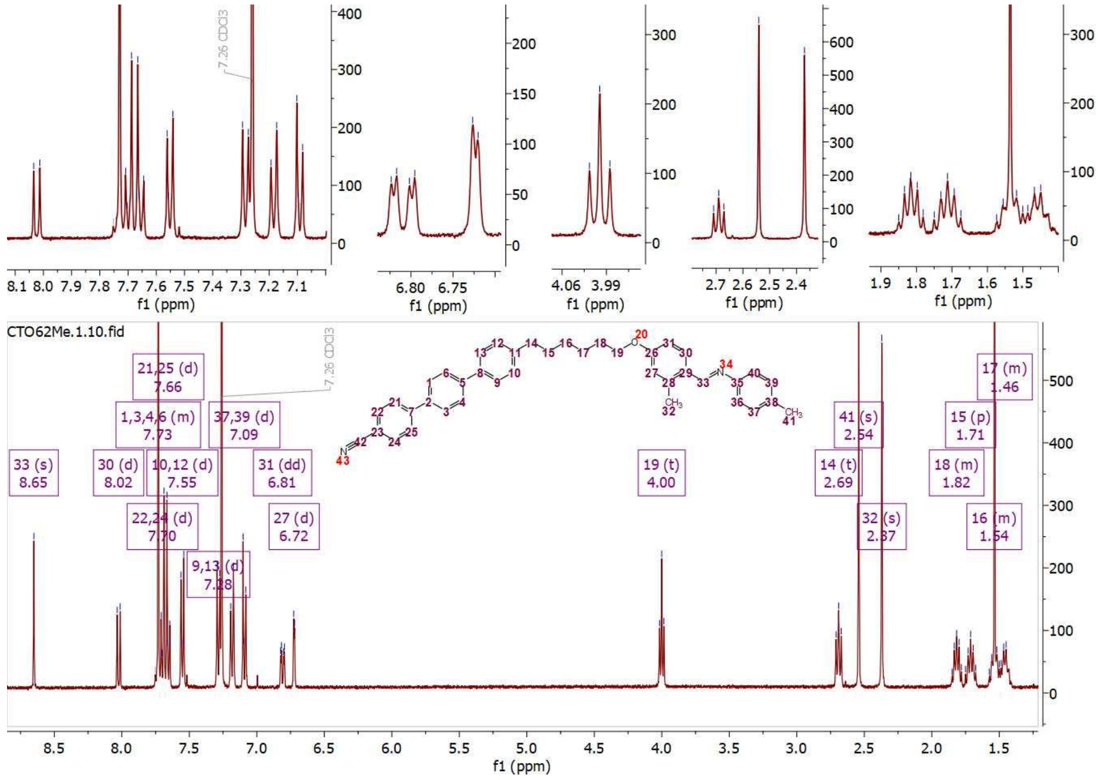

Supplementary Figure 2. ^{1}H NMR spectrum of CT6O2Me.1 (400 MHz, $CDCl_3$).

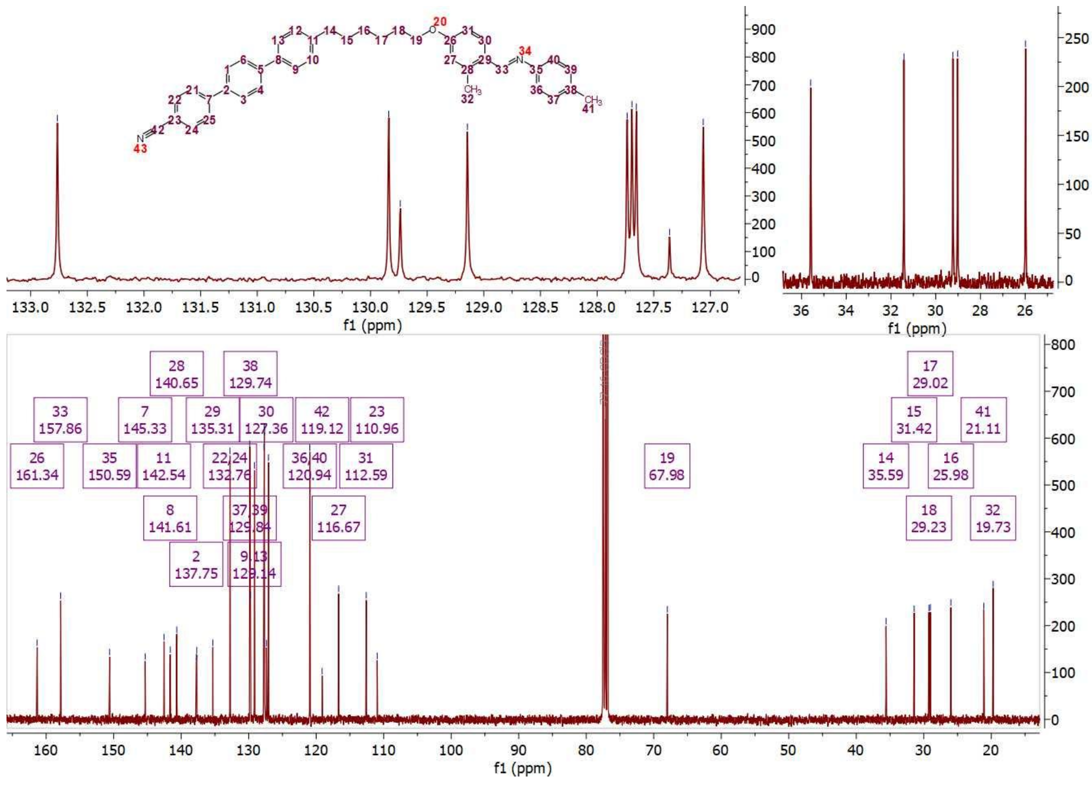

Supplementary Figure 3. ^{13}C NMR spectrum of CT6O2Me.1 (101 MHz, $CDCl_3$).

CT6O2Me.2

Yield: 89 %. m.p. 139 °C, ($T_{NTB\text{-}N}$ 114), $T_{N\text{-}I}$ 202 °C.

^{1}H NMR (400 MHz, $CDCl_3$) δ ppm: 8.66 (s, 1H, $\underline{CH}$=N), 8.03 (d, ^3J = 8.7 Hz, 1H, Ar-H), 7.73 (m, appt s, 4H, Ar-H), 7.70 (d, ^3J = 8.3 Hz, 2H, Ar-H), 7.66 (d, ^3J = 8.3 Hz, 2H, Ar-H), 7.55 (d, ^3J = 7.9 Hz, 2H, Ar-H), 7.29 (d, ^3J = 7.9 Hz, 2H, Ar-H), 7.21 (d, ^3J = 8.1 Hz, 2H, Ar-H), 7.12 (d, ^3J = 8.1 Hz, 2H, Ar-H), 6.81 (dd, ^3J = 8.7 Hz, ^4J = 2.5 Hz, 1H, Ar-H), 6.73 (d, ^4J = 2.5 Hz, 1H, Ar-H), 4.00 (t, ^3J = 6.5 Hz, 2H, ArCH$_2$(CH$_2$)$_4$$\underline{CH_2}$-O), 2.68 (m, 4H, Ar$\underline{CH_2}$(CH$_2$)$_4CH_2$-O, Ar$\underline{CH_2}CH_3$), 2.54 (s, 3H, Ar$\underline{CH_3}$), 1.82 (m, 2H, Ar(CH$_2$)$_4$$\underline{CH_2}CH_2$-O), 1.71 (p, ^3J = 7.5 Hz, 2H, ArCH$_2$$\underline{CH_2}$(CH$_2$)$_4$-O), 1.54 (m, 2H, Ar(CH$_2$)$_3$$\underline{CH_2}CH_2CH_2$-O), 1.46 (m, 2H, ArCH$_2CH_2$$\underline{CH_2}$(CH$_2$)$_3$-O), 1.26 (t, 7.6 Hz, 3H, ArCH$_2$$\underline{CH_3}$). ^{13}C NMR (101 MHz, $CDCl_3$) δ ppm: 161.34, 157.90, 150.80, 145.34, 142.54, 141.76, 141.62, 140.66, 137.76, 137.69, 132.77 (2C), 129.76, 129.15 (2C), 128.65 (2C), 127.74 (2C), 127.70 (2C), 127.66 (2C), 127.39, 127.07 (2C), 121.01 (2C), 119.12, 116.67, 112.59, 110.97, 67.99, 35.60, 31.43, 29.23, 29.03, 28.55, 25.99, 19.74, 15.86.

IR (vmax/cm^{-1}): 2918, 2850 (C-H, sp^2), 2221 (C≡N stretch), 1602 (para disubstituted benzene), 1585, 1504, 1488, 1259, 1102, 1003, 808, 517.

HRMS: (TOF ESI$^+$) (m/z): [M+H]$^+$ Calculated for $C_{41}H_{41}N_2O$ =577.3219; found, 577.3191 (- 4.9 PPM difference).

CT6O2Me.3

Yield: 82 %. m.p. 134 °C, ($T_{NTB\text{-}N}$ 122 °C), $T_{N\text{-}I}$ 201 °C.

^{1}H NMR (400 MHz, $CDCl_3$) δ ppm: 8.66 (s, 1H, $\underline{CH}$=N), 8.02 (d, ^3J = 8.7 Hz, 1H, Ar-H), 7.73 (m, appt s, 4H, Ar-H), 7.70 (d, ^3J = 8.3 Hz, 2H, Ar-H), 7.66 (d, ^3J = 8.3 Hz, 2H, Ar-H), 7.55 (d, ^3J = 7.9 Hz, 2H, Ar-H), 7.28 (d, ^3J = 7.9 Hz, 2H, Ar-H), 7.19 (d, ^3J = 8.0 Hz, 2H, Ar-H), 7.11 (d, ^3J = 8.1 Hz, 2H, Ar-H), 6.81 (dd, ^3J = 8.7 Hz, ^4J = 2.6 Hz, 1H, Ar-H), 6.72 (d, ^4J = 2.6 Hz, 1H, Ar-H), 4.00 (t, ^3J = 6.4 Hz, 2H, ArCH$_2$(CH$_2$)$_4$$\underline{CH_2}$-O), 2.69 (t, ^3J = 7.7 Hz, 2H, Ar$\underline{CH_2}$(CH$_2$)$_4$CH$_2$-O), 2.60 (t, ^3J = 7.6 Hz, 2H, Ar$\underline{CH_2}$CH$_2$CH$_3$), 2.54 (s, 3H, Ar$\underline{CH_3}$), 1.82 (m, 2H, Ar(CH$_2$)$_4$$\underline{CH_2}CH_2$-O), 1.68 (m, 4H,

$ArCH_2\underline{CH_2}(CH_2)_4$-O, $ArCH_2\underline{CH_2}CH_3$), 1.53 (m, 2H, $Ar(CH_2)_3\underline{CH_2}CH_2CH_2$-O), 1.46 (m, 2H, $ArCH_2CH_2\underline{CH_2}(CH_2)_3$-O), 0.96 (t, 7.3 Hz, 3H, $ArCH_2CH_2\underline{CH_3}$). ^{13}C NMR (101 MHz, $CDCl_3$) δ ppm: 161.33, 157.86, 150.78, 145.35, 142.55, 141.62, 140.66, 140.21, 137.77, 137.69, 132.77 (2C), 129.77, 129.26 (2C), 129.15 (2C), 127.75 (2C), 127.70 (2C), 127.66 (2C), 127.40, 127.07 (2C), 120.92 (2C), 119.12, 116.68, 112.58, 110.97, 67.99, 37.71, 35.60, 31.43, 29.24, 29.03, 25.99, 24.81, 19.75, 13.97. IR (vmax/cm^{-1}): 2919, 2851 (C-H, sp^2), 2221 (C≡N stretch), 1603 (para disubstituted benzene), 1585, 1488, 1259, 1102, 1002, 807, 516. HRMS: (TOF ESI^+) (m/z): $[M+H]^+$ Calculated for $C_{42}H_{43}N_2O$ = 591.3375; found, 591.3356 (- 3.2 PPM difference).

CT6O2Me.4

Yield: 92 %. m.p. 122 °C, ($T_{SmCTBC\text{-}NTB}$ 86 °C), ($T_{NTB\text{-}N}$ 115 °C), $T_{N\text{-}I}$ 193 °C.

^1H NMR (400 MHz, $CDCl_3$) δ ppm: 8.66 (s, 1H, $\underline{CH}$=N), 8.02 (d, ^3J = 8.7 Hz, 1H, Ar-H), 7.73 (m, appt s, 4H, Ar-H), 7.70 (d, ^3J = 8.6 Hz, 2H, Ar-H), 7.66 (d, ^3J = 8.6 Hz, 2H, Ar-H), 7.55 (d, ^3J = 8.1 Hz, 2H, Ar-H), 7.28 (d, ^3J = 8.1 Hz, 2H, Ar-H), 7.19 (d, ^3J = 8.2 Hz, 2H, Ar-H), 7.11 (d, ^3J = 8.2 Hz, 2H, Ar-H), 6.81 (dd, ^3J = 8.7 Hz, ^4J = 2.6 Hz, 1H, Ar-H), 6.72 (d, ^4J = 2.6 Hz, 1H, Ar-H), 4.00 (t, ^3J = 6.5 Hz, 2H, $ArCH_2(CH_2)_4\underline{CH_2}$-O), 2.69 (t, ^3J = 7.6 Hz, 2H, $Ar\underline{CH_2}(CH_2)_4CH_2$-O), 2.63 (t, ^3J = 7.7 Hz, 2H, $Ar\underline{CH_2}CH_2CH_2CH_3$), 2.54 (s, 3H, $Ar\underline{CH_3}$), 1.82 (m, 2H, $Ar(CH_2)_4\underline{CH_2}CH_2$-O), 1.71 (p, ^3J = 7.6 Hz, 2H, $ArCH_2\underline{CH_2}(CH_2)_4$-O), 1.62 (m, 2H, $ArCH_2\underline{CH_2}CH_2CH_3$), 1.53 (m, 2H, $Ar(CH_2)_3\underline{CH_2}CH_2CH_2$-O), 1.45 (m, 2H, $ArCH_2CH_2\underline{CH_2}(CH_2)_3$-O), 1.37 (h, ^3J = 7.4 Hz, 2H, $ArCH_2CH_2\underline{CH_2}CH_3$), 0.94 (t, ^3J = 7.4 Hz, 3H, $ArCH_2CH_2CH_2\underline{CH_3}$). ^{13}C NMR (101 MHz, $CDCl_3$) δ ppm: 161.33, 157.85, 150.74, 145.35, 142.55, 141.63, 140.66, 140.44, 137.77, 137.70, 132.78 (2C), 129.77, 129.21 (2C), 129.15 (2C), 127.75 (2C), 127.70 (2C), 127.67 (2C), 127.41, 127.08 (2C), 120.93 (2C), 119.13, 116.68, 112.59, 110.98, 68.00, 35.61, 35.32, 33.90, 31.43, 29.24, 29.04, 25.99, 22.50, 19.75, 14.12. IR (vmax/cm^{-1}): 2920, 2852 (C-H, sp^2), 2222 (C≡N stretch), 1603, (para disubstituted benzene), 1586, 1488, 1259, 1102, 808, 517. HRMS: (TOF ESI^+) (m/z): $[M+H]^+$ Calculated for $C_{43}H_{45}N_2O$ = 605.3532; found, 605.3480 (- 4.0 PPM difference).

CT6O2Me.5

Yield: 96 %, m.p. 128 °C, ($T_{SmCTBC\text{-}NTB}$ 75 °C), ($T_{NTB\text{-}N}$ 117 °C), $T_{N\text{-}I}$ 191 °C

^{1}H NMR (400 MHz, $CDCl_3$) δ ppm: 8.66 (s, 1H, $\underline{CH}$=N), 8.02 (d, ^3J = 8.7 Hz, 1H, Ar-H), 7.73 (m, appt s, 4H, Ar-H), 7.70 (d, ^3J = 8.7 Hz, 2H, Ar-H), 7.65 (d, ^3J = 8.7 Hz, 2H, Ar-H), 7.55 (d, ^3J = 8.1 Hz, 2H, Ar-H), 7.28 (d, ^3J = 8.1 Hz, 2H, Ar-H), 7.19 (d, ^3J = 8.2 Hz, 2H, Ar-H), 7.11 (d, ^3J = 8.2 Hz, 2H, Ar-H), 6.81 (dd, ^3J = 8.7 Hz, ^4J = 2.5 Hz, 1H, Ar-H), 6.72 (d, ^4J = 2.5 Hz, 1H, Ar-H), 4.00 (t, ^3J = 6.5 Hz, 2H, $ArCH_2(CH_2)_4\underline{CH_2}$-O), 2.69 (t, ^3J = 7.6 Hz, 2H, $Ar\underline{CH_2}(CH_2)_4CH_2$-O), 2.62 (t, ^3J = 7.8 Hz, 2H, $Ar\underline{CH_2}(CH_2)_3CH_3$), 2.54 (s, 3H, $Ar\underline{CH_3}$), 1.82 (m, 2H, $Ar(CH_2)_4\underline{CH_2}CH_2$-O), 1.71 (p, ^3J = 7.6 Hz, 2H, $ArCH_2\underline{CH_2}(CH_2)_4$-O), 1.62 (p, ^3J = 7.8 Hz, 2H, $ArCH_2\underline{CH_2}(CH_2)_2CH_3$), 1.55 (m, 2H, $Ar(CH_2)_3\underline{CH_2}CH_2CH_2$-O), 1.48 (m, 2H, $ArCH_2CH_2\underline{CH_2}(CH_2)_3$-O), 1.34 (m, 4H, $ArCH_2CH_2\underline{CH_2}\underline{CH_2}CH_3$), 0.90 (t, ^3J = 6.8 Hz, 3H, $Ar(CH_2)_4\underline{CH_3}$). ^{13}C NMR (101 MHz, $CDCl_3$) δ ppm: 161.33, 157.85, 150.74, 145.35, 142.55, 141.63, 140.66, 140.48, 137.77, 137.70, 132.78 (2C), 129.77, 129.20 (2C), 129.15 (2C), 127.75 (2C), 127.70 (2C), 127.67 (2C), 127.41, 127.07 (2C), 120.94 (2C), 119.12, 116.68, 112.58, 110.98, 68.00, 35.60, 31.65, 31.43 (2C), 31.08, 29.24, 29.04, 25.99, 22.71, 19.75, 14.20. IR (νmax/cm^{-1}): 2920, 2852 (C-H, sp^2), 2224 (C≡N stretch), 1603 (para disubstituted benzene), 1586, 1504, 1488, 1259, 1102, 1003, 808, 516. HRMS: (TOF ESI^+) (m/z): $[M+H]^+$ Calculated for $C_{44}H_{47}N_2O$ = 619.3688; found, 619.3675 (- 2.1 PPM difference).

CT6O2Me.6

Yield: 88 %. m.p. 100 °C, ($T_{SmCTBC\text{-}NTB}$ 86 °C), $T_{NTB\text{-}N}$ 108 °C, $T_{N\text{-}I}$ 183 °C.

^{1}H NMR (400 MHz, $CDCl_3$) δ ppm: 8.66 (s, 1H, $\underline{CH}$=N), 8.02 (d, ^3J = 8.7 Hz, 1H, Ar-H), 7.73 (m, appt s, 4H, Ar-H), 7.70 (d, ^3J = 8.5 Hz, 2H, Ar-H), 7.65 (d, ^3J = 8.5 Hz, 2H, Ar-H), 7.55 (d, ^3J = 8.1 Hz, 2H, Ar-H), 7.29 (d, ^3J = 8.1 Hz, 2H, Ar-H), 7.19 (d, ^3J = 8.3 Hz, 2H, Ar-H), 7.11 (d, ^3J = 8.3 Hz, 2H, Ar-H), 6.81 (dd, ^3J = 8.7 Hz, ^4J = 2.6 Hz, 1H, Ar-H), 6.72 (d, ^4J = 2.6 Hz, 1H, Ar-H), 4.00 (t, ^3J = 6.5 Hz, 2H, $ArCH_2(CH_2)_4\underline{CH_2}$-O), 2.69 (t, ^3J = 7.6 Hz, 2H, $Ar\underline{CH_2}(CH_2)_4CH_2$-O), 2.62 (t, ^3J = 7.7 Hz, 2H, $Ar\underline{CH_2}(CH_2)_4CH_3$), 2.54 (s, 3H, $Ar\underline{CH_3}$), 1.82 (m, 2H, $Ar(CH_2)_4\underline{CH_2}CH_2$-O), 1.71 (p, ^3J = 7.6 Hz, 2H, $ArCH_2\underline{CH_2}(CH_2)_4$-O), 1.63 (m, 2H, $ArCH_2\underline{CH_2}(CH_2)_3CH_3$), 1.56 (m, 2H, $Ar(CH_2)_3\underline{CH_2}CH_2CH_2$-

O), 1.46 (m, 2H, $ArCH_2CH_2\underline{CH_2}(CH_2)_3$-O), 1.32 (m, 6H, $ArCH_2CH_2\underline{(CH_2)_3}CH_3$), 0.89 (t, ^3J = 6.8 Hz, 3H, $Ar(CH_2)_5\underline{CH_3}$). ^{13}C NMR (101 MHz, $CDCl_3$) δ ppm: 161.33, 157.84, 150.73, 145.34, 142.55, 141.62, 140.65, 140.48, 137.77, 137.69, 132.77 (2C), 129.77, 129.19 (2C), 129.15 (2C), 127.74 (2C), 127.70 (2C), 127.66 (2C), 127.41, 127.07 (2C), 120.93 (2C), 119.12, 116.68, 112.58, 110.97, 67.99, 35.63, 35.60, 31.89, 31.72, 31.43, 29.24, 29.13, 29.04, 25.99, 22.77, 19.75, 14.25. IR (νmax/cm^{-1}): 2920, 2852 (C-H, sp^2), 2225 (C≡N stretch), 1604 (para disubstituted benzene), 1586, 1488, 1243, 1101, 1003, 809, 516. HRMS: (TOF ESI^+) (m/z): $[M+H]^+$ Calculated for $C_{45}H_{49}N_2O$ = 633.3845; found, 633.3832 (- 2.1 PPM difference).

CT6O2Me.7

Yield: 91%. m.p. 112 °C, ($T_{SmCTBC\text{-}NTB}$ 74 °C), ($T_{NTB\text{-}N}$ 110 °C), $T_{N\text{-}I}$ 180 °C.

^1H NMR (400 MHz, $CDCl_3$) δ ppm: 8.66 (s, 1H, $\underline{CH}$=N), 8.02 (d, ^3J = 8.6 Hz, 1H, Ar-H), 7.73 (m, appt s, 4H, Ar-H), 7.70 (d, ^3J = 8.3 Hz, 2H, Ar-H), 7.66 (d, ^3J = 8.3 Hz, 2H, Ar-H), 7.55 (d, ^3J = 8.1 Hz, 2H, Ar-H), 7.28 (d, ^3J = 8.1 Hz, 2H, Ar-H), 7.19 (d, ^3J = 8.2 Hz, 2H, Ar-H), 7.11 (d, ^3J = 8.2 Hz, 2H, Ar-H), 6.81 (dd, ^3J = 8.6 Hz, ^4J = 2.5 Hz, 1H, Ar-H), 6.72 (d, ^4J = 2.5 Hz, 1H, Ar-H), 4.00 (t, ^3J = 6.4 Hz, 2H, $ArCH_2(CH_2)_4\underline{CH_2}$-O), 2.69 (t, ^3J = 7.7 Hz, 2H, $Ar\underline{CH_2}(CH_2)_4CH_2$-O), 2.62 (t, ^3J = 7.8 Hz, 2H, $Ar\underline{CH_2}(CH_2)_5CH_3$), 2.54 (s, 3H, $Ar\underline{CH_3}$), 1.82 (m, 2H, $Ar(CH_2)_4\underline{CH_2}CH_2$-O), 1.71 (p, ^3J = 7.7 Hz, 2H, $ArCH_2\underline{CH_2}(CH_2)_4$-O), 1.62 (m, 2H, $ArCH_2\underline{CH_2}(CH_2)_4CH_3$), 1.50 (m, 2H, $Ar(CH_2)_3\underline{CH_2}CH_2CH_2$-O), 1.46 (m, 2H, $ArCH_2CH_2\underline{CH_2}(CH_2)_3$-O), 1.31 (m, 8H, $ArCH_2CH_2\underline{(CH_2)_4}CH_3$), 0.88 (t, ^3J = 6.8 Hz, 3H, $Ar(CH_2)_6\underline{CH_3}$). ^{13}C NMR (101 MHz, $CDCl_3$) δ ppm: 161.32, 157.84, 150.72, 145.34, 142.54, 141.62, 140.65, 140.48, 137.76, 137.69, 132.77 (2C), 129.76, 129.19 (2C), 129.15 (2C), 127.74 (2C), 127.70 (2C), 127.66 (2C), 127.41, 127.07 (2C), 120.93 (2C), 119.12, 116.67, 112.58, 110.97, 67.99, 35.63, 35.60, 31.98, 31.76, 31.43, 29.42, 29.34, 29.24, 29.04, 25.99, 22.82, 19.75, 14.26. IR (νmax/cm^{-1}): 2920, 2856 (C-H, sp^2), 2225 (C≡N stretch), 1603 (para disubstituted benzene), 1586, 1488, 1258, 1102, 807, 519. HRMS: (TOF ESI^+) (m/z): $[M+H]^+$ Calculated for $C_{46}H_{51}N_2O$ = 647.4001; found, 647.3984 (- 2.1 PPM difference).

CT6O2Me.8

Yield: 90 %. m.p. 106 °C, ($T_{SmCTBC\text{-}SmCTBDH}$ 65 °C), ($T_{SmCTBDH\text{-}SmCTBSH}$ 87 °C), ($T_{SmCTBSH\text{-}SmA}$ 99 °C), $T_{SmA\text{-}N}$ 143 °C, $T_{N\text{-}I}$ 174 °C.

^{1}H NMR (400 MHz, $CDCl_3$) δ ppm: 8.66 (s, 1H, CH=N), 8.02 (d, ^3J = 8.7 Hz, 1H, Ar-H), 7.73 (m, appt s, 4H, Ar-H), 7.70 (d, ^3J = 8.7 Hz, 2H, Ar-H), 7.66 (d, ^3J = 8.7 Hz, 2H, Ar-H), 7.55 (d, ^3J = 8.2 Hz, 2H, Ar-H), 7.28 (d, ^3J = 8.2 Hz, 2H, Ar-H), 7.19 (d, ^3J = 8.3 Hz, 2H, Ar-H), 7.11 (d, ^3J = 8.3 Hz, 2H, Ar-H), 6.81 (dd, ^3J = 8.7 Hz, ^4J = 2.6 Hz, 2H, Ar-H), 6.72 (d, ^4J = 2.6 Hz, 1H, Ar-H), 4.00 (t, ^3J = 6.4 Hz, 2H, $ArCH_2(CH_2)_4\underline{CH_2}$-O), 2.69 (t, ^3J = 7.6 Hz, 2H, $Ar\underline{CH_2}(CH_2)_4CH_2$-O), 2.62 (t, ^3J = 7.7 Hz, 2H, $Ar\underline{CH_2}(CH_2)_6CH_3$), 2.54 (s, 3H, $Ar\underline{CH_3}$), 1.82 (m, 2H, $Ar(CH_2)_4\underline{CH_2}CH_2$-O), 1.71 (p, ^3J = 7.6 Hz, 2H, $ArCH_2\underline{CH_2}(CH_2)_4$-O), 1.61 (m, 2H, $ArCH_2\underline{CH_2}(CH_2)_5CH_3$), 1.53 (m, 2H, $Ar(CH_2)_3\underline{CH_2}CH_2CH_2$-O), 1.47 (m, 2H, $ArCH_2CH_2\underline{CH_2}(CH_2)_3$-O), 1.30 (m, 10H, $ArCH_2CH_2\underline{(CH_2)_5}CH_3$), 0.88 (t, ^3J = 6.8 Hz, 3H, $Ar(CH_2)_7\underline{CH_3}$). ^{13}C NMR (101 MHz, $CDCl_3$) δ ppm: 161.33, 157.85, 150.73, 145.35, 142.55, 141.63, 140.65, 140.49, 137.77, 137.70, 132.78 (2C), 129.77, 129.20 (2C), 129.15 (2C), 127.75 (2C), 127.70 (2C), 127.67 (2C), 127.41, 127.08 (2C), 120.93 (2C), 119.13, 116.68, 112.59, 110.98, 68.00, 35.64, 35.61, 32.04, 31.76, 31.44, 29.64, 29.48, 29.43, 29.24, 29.05, 26.00, 22.82, 19.75, 14.26. IR (vmax/cm^{-1}): 2920, 2844 (C-H, sp^2), 2227 (C≡N stretch), 1607 (para disubstituted benzene), 1590, 1488, 1257, 1032, 1004, 812, 519. HRMS: (TOF ESI$^+$) (m/z): $[M+H]^+$ Calculated for $C_{47}H_{53}N_2O$ = 661.4158; found, 661.4155 (- 0.5 PPM difference).

CT6O2Me.9

Yield: 94 %. m.p. 104 °C, ($T_{SmCTBDH\text{-}SmCTBSH}$ 84 °C), ($T_{SmCTBSH\text{-}SmA}$ 97 °C), $T_{SmA\text{-}N}$ 155 °C, $T_{N\text{-}I}$ 173 °C.

^{1}H NMR (400 MHz, $CDCl_3$) δ ppm: 8.66 (s, 1H, CH=N), 8.02 (d, ^3J = 8.7 Hz, 1H, Ar-H), 7.73 (m, appt s, 4H, Ar-H), 7.70 (d, ^3J = 8.7 Hz, 2H, Ar-H), 7.66 (d, ^3J = 8.7 Hz, 2H, Ar-H), 7.55 (d, ^3J = 8.2 Hz, 2H, Ar-H), 7.28 (d, ^3J = 8.2 Hz, 2H, Ar-H), 7.19 (d, ^3J = 8.3 Hz, 2H, Ar-H), 7.11 (d, ^3J = 8.3 Hz, 2H, Ar-H), 6.81 (dd, ^3J = 8.7 Hz, ^4J = 2.6 Hz, 1H, Ar-H), 6.72 (d, ^4J = 2.6 Hz, 1H, Ar-H), 4.00 (t, ^3J = 6.4 Hz, 2H, $ArCH_2(CH_2)_4\underline{CH_2}$-O), 2.69 (t, ^3J = 7.6 Hz, 2H, $Ar\underline{CH_2}(CH_2)_4CH_2$-O), 2.62 (t, ^3J = 7.6 Hz, 2H, $Ar\underline{CH_2}(CH_2)_7CH_3$), 2.54 (s, 3H, $Ar\underline{CH_3}$), 1.82 (m, 2H, $Ar(CH_2)_4\underline{CH_2}CH_2$-O), 1.71 (p, ^3J = 7.6 Hz,

2H, $ArCH_2\underline{CH_2}(CH_2)_4$-O), 1.61 (m, 2H, $ArCH_2\underline{CH_2}(CH_2)_6CH_3$), 1.53 (m, 2H, $Ar(CH_2)_3\underline{CH_2}CH_2CH_2$-O), 1.47 (m, 2H, $ArCH_2CH_2\underline{CH_2}(CH_2)_3$-O), 1.30 (m, 12H, $ArCH_2CH_2\underline{(CH_2)_6}CH_3$), 0.88 (t, ^3J = 6.7 Hz, 3H, $Ar(CH_2)_8\underline{CH_3}$). ^{13}C NMR (101 MHz, $CDCl_3$) δ ppm: 161.33, 157.84, 150.72, 145.34, 142.55, 141.62, 140.65, 140.49, 137.76, 137.69, 132.77 (2C), 129.76, 129.19 (2C), 129.15 (2C), 127.74 (2C), 127.70 (2C), 127.66 (2C), 127.41, 127.07 (2C), 120.93 (2C), 119.12, 116.67, 112.58, 110.97, 67.99, 35.63, 35.61, 32.05, 31.76, 31.43, 29.72, 29.68, 29.48, 29.47, 29.24, 29.04, 25.99, 22.82, 19.75, 14.26. IR (νmax/cm^{-1}): 2920, 2844 (C-H, sp^2), 2227 (C≡N stretch), 1607 (para disubstituted benzene), 1590, 1488, 1257, 1001, 812, 519. HRMS: (TOF ESI^+) (m/z): $[M+H]^+$ Calculated for $C_{48}H_{55}N_2O$ = 675.4314; found, 675.4277 (- 0.5 PPM difference).

CT6O2Me.10

Yield: 73 %. ($T_{SmCTBDH\text{-}SmCTBSH}$ 80°C), ($T_{SmCTBSH\text{-}SmA}$ 93°C), $T_{SmA\text{-}N}$ 158 °C, $T_{N\text{-}I}$ 167 °C.

^{1}H NMR (400 MHz, $CDCl_3$) δ ppm: 8.66 (s, 1H, $\underline{CH}$=N), 8.02 (d, ^3J = 8.7 Hz, 1H, Ar-H), 7.73 (m, appt s, 4H, Ar-H), 7.70 (d, ^3J = 8.6 Hz, 2H, Ar-H), 7.66 (d, ^3J = 8.6 Hz, 2H, Ar-H), 7.55 (d, ^3J = 8.1 Hz, 2H, Ar-H), 7.28 (d, ^3J = 8.1 Hz, 2H, Ar-H), 7.18 (d, ^3J = 8.3 Hz, 2H, Ar-H), 7.11 (d, ^3J = 8.3 Hz, 2H, Ar-H), 6.81 (dd, ^3J = 8.7 Hz, ^4J = 2.6 Hz, 1H, Ar-H), 6.72 (d, ^4J = 2.6 Hz, 1H, Ar-H), 4.00 (t, ^3J = 6.5 Hz, 2H, $ArCH_2(CH_2)_4\underline{CH_2}$-O), 2.69 (t, ^3J = 7.6 Hz, 2H, $Ar\underline{CH_2}(CH_2)_4CH_2$-O), 2.62 (t, ^3J = 7.8 Hz, 2H, $Ar\underline{CH_2}(CH_2)_8CH_3$), 2.54 (s, 3H, $Ar\underline{CH_3}$), 1.82 (m, 2H, $Ar(CH_2)_4\underline{CH_2}CH_2$-O), 1.71 (p, ^3J = 7.6 Hz, 2H, $ArCH_2\underline{CH_2}(CH_2)_4$-O), 1.61 (m, 2H, $ArCH_2\underline{CH_2}(CH_2)_7CH_3$), 1.54 (m, 2H, $Ar(CH_2)_3\underline{CH_2}CH_2CH_2$-O), 1.46 (m, 2H, $ArCH_2CH_2\underline{CH_2}(CH_2)_3$-O), 1.29 (m, 14H, $ArCH_2CH_2\underline{(CH_2)_7}CH_3$), 0.88 (t, ^3J = 6.6 Hz, 3H, $Ar(CH_2)_9\underline{CH_3}$). ^{13}C NMR (101 MHz, $CDCl_3$) δ ppm: 161.33, 157.85, 150.73, 145.35, 142.55, 141.63, 140.66, 140.50, 137.77, 137.70, 132.78 (2C), 129.77, 129.20 (2C), 129.15 (2C), 127.75 (2C), 127.70 (2C), 127.67 (2C), 127.41, 127.08 (2C), 120.93 (2C), 119.12, 116.68, 112.59, 110.98, 68.00, 35.64, 35.61, 32.06, 31.76, 31.44, 29.78, 29.76, 29.68, 29.49, 29.47, 29.25, 29.05, 26.00, 22.84, 19.75, 14.27. IR (νmax/cm^{-1}): 2919, 2846 (C-H, sp^2), 2232 (C≡N stretch), 1607 (para disubstituted benzene), 1590, 1477, 1255, 812, 520. HRMS: (TOF ESI^+) (m/z): $[M+H]^+$ Calculated for $C_{49}H_{57}N_2O$ = 689.4471; found, 689.4472 (0.1 PPM difference).

CT6O2Me.11

Yield: 85 %. m.p. 94 °C, ($T_{SmCTBDH\text{-}SmCTBSH}$ 77 °C), ($T_{SmCTBSH\text{-}SmA}$ 88 °C), $T_{SmA\text{-}N}$ 158 °C, $T_{N\text{-}I}$ 164 °C.

^{1}H NMR (400 MHz, $CDCl_3$) δ ppm: 8.66 (s, 1H, $\underline{CH}$=N), 8.02 (d, ^{3}J = 8.7 Hz, 1H, Ar-H), 7.73 (m, appt s, 4H, Ar-H), 7.70 (d, ^{3}J = 8.7 Hz, 2H, Ar-H), 7.66 (d, ^{3}J = 8.7 Hz, 2H, Ar-H), 7.55 (d, ^{3}J = 8.1 Hz, 2H, Ar-H), 7.28 (d, ^{3}J = 8.1 Hz, 2H, Ar-H), 7.19 (d, ^{3}J = 8.2 Hz, 2H, Ar-H), 7.11 (d, ^{3}J = 8.2 Hz, 2H, Ar-H), 6.81 (dd, ^{3}J = 8.7 Hz, ^{4}J = 2.6 Hz, 1H, Ar-H), 6.72 (d, ^{4}J = 2.6 Hz, 1H, Ar-H), 4.00 (t, ^{3}J = 6.5 Hz, 2H, $ArCH_2(CH_2)_4\underline{CH_2}$-O), 2.69 (t, ^{3}J = 7.6 Hz, 2H, $Ar\underline{CH_2}(CH_2)_4CH_2$-O), 2.61 (t, ^{3}J = 7.6 Hz, 2H, $Ar\underline{CH_2}(CH_2)_9CH_3$), 2.54 (s, 3H, $Ar\underline{CH_3}$), 1.82 (m, 2H, $Ar(CH_2)_4\underline{CH_2}CH_2$-O), 1.71 (p, ^{3}J = 7.6 Hz, 2H, $ArCH_2\underline{CH_2}(CH_2)_4$-O), 1.62 (m, 2H, $ArCH_2\underline{CH_2}(CH_2)_8CH_3$), 1.54 (m, 2H, $Ar(CH_2)_3\underline{CH_2}CH_2CH_2$-O), 1.47 (m, 2H, $ArCH_2CH_2\underline{CH_2}(CH_2)_3$-O), 1.26 (m, 16H, $ArCH_2CH_2\underline{(CH_2)_8}CH_3$), 0.88 (t, ^{3}J = 6.7 Hz, 3H, $Ar(CH_2)_{10}\underline{CH_3}$). ^{13}C NMR (101 MHz, $CDCl_3$) δ ppm: 161.33, 157.85, 150.73, 145.36, 142.55, 141.63, 140.65, 140.50, 137.78, 137.70, 132.78 (2C), 129.77, 129.20 (2C), 129.15 (2C), 127.75 (2C), 127.71 (2C), 127.67 (2C), 127.42, 127.08 (2C), 120.93 (2C), 119.13, 116.68, 112.59, 110.98, 68.00, 35.64, 35.61, 32.07, 31.77, 31.44, 29.83, 29.79, 29.76, 29.69, 29.50, 29.48, 29.25, 29.05, 26.00, 22.84, 19.75, 14.27. IR (νmax/cm^{-1}): 2918, 2846 (C-H, sp^2), 2232 (C≡N stretch), 1607 (para disubstituted benzene), 1590, 1476, 1254, 1030, 812, 503. HRMS: (TOF ESI^+) (m/z): $[M+H]^+$ Calculated for $C_{50}H_{59}N_2O$ = 703.4627; found, 703.4604 (- 3.3 PPM difference).

CT6O2Me.12

Yield: 82 %. m.p. 93 °C, ($T_{SmCTBDH\text{-}SmCTBSH}$ 82 °C), ($T_{SmCTBSH\text{-}SmA}$ 86 °C), $T_{SmA\text{-}N}$ 162 °C, $T_{N\text{-}I}$ 164 °C.

^{1}H NMR (400 MHz, $CDCl_3$) δ ppm: 8.66 (s, 1H, $\underline{CH}$=N), 8.02 (d, ^{3}J = 8.7 Hz, 1H, Ar-H), 7.73 (m, appt s, 4H, Ar-H), 7.70 (d, ^{3}J = 8.6 Hz, 2H, Ar-H), 7.66 (d, ^{3}J = 8.6 Hz, 2H, Ar-H), 7.55 (d, ^{3}J = 8.1 Hz, 2H, Ar-H), 7.29 (d, ^{3}J = 8.1 Hz, 2H, Ar-H), 7.19 (d, ^{3}J = 8.3 Hz, 2H, Ar-H), 7.11 (d, ^{3}J = 8.3 Hz, 2H, Ar-H), 6.81 (dd, ^{3}J = 8.7 Hz, ^{4}J = 2.6 Hz, 1H, Ar-H), 6.72 (d, ^{4}J = 2.6 Hz, 1H, Ar-H), 4.00 (t, ^{3}J = 6.5 Hz, 2H, $ArCH_2(CH_2)_4\underline{CH_2}$-O), 2.69 (t, ^{3}J = 7.6 Hz, 2H, $Ar\underline{CH_2}(CH_2)_4CH_2$-O), 2.61 (t, ^{3}J = 7.9 Hz, 2H, $Ar\underline{CH_2}(CH_2)_{10}CH_3$), 2.54 (s, 3H, $Ar\underline{CH_3}$), 1.82 (m, 2H, $Ar(CH_2)_4\underline{CH_2}CH_2$-O), 1.71 (p, ^{3}J = 7.6 Hz, 2H, $ArCH_2\underline{CH_2}(CH_2)_4$-O), 1.61 (m, 2H, $ArCH_2\underline{CH_2}(CH_2)_9CH_3$), 1.50 (m, 2H, $Ar(CH_2)_3\underline{CH_2}CH_2CH_2$-

O), 1.46 (m, 2H, ArCH$_2$CH$_2$CH$_2$(CH$_2$)$_3$-O), 1.26 (m, 18H, ArCH$_2$CH$_2$(CH$_2$)$_9$CH$_3$), 0.88 (t, ^3J = 6.7 Hz, 3H, Ar(CH$_2$)$_{11}$CH$_3$). ^{13}C NMR (101 MHz, CDCl$_3$) δ ppm: 161.33, 157.85, 150.73, 145.36, 142.55, 141.63, 140.66, 140.50, 137.78, 137.70, 132.78 (2C), 129.77, 129.20 (2C), 129.15 (2C), 127.75 (2C), 127.71 (2C), 127.67 (2C), 127.42, 127.08 (2C), 120.93 (2C), 119.13, 116.68, 112.59, 110.98, 68.00, 35.64, 35.61, 32.07, 31.77, 31.44, 29.83, 29.82, 29.79, 29.76, 29.68, 29.51, 29.48, 29.25, 29.05, 26.00, 22.84, 19.75, 14.27. IR (νmax/cm^{-1}): 2916, 2847 (C-H, sp^2), 2229 (C≡N stretch), 1607 (para disubstituted benzene), 1591,1488, 1257, 812, 519. HRMS: (TOF ESI$^+$) (m/z): [M+H]$^+$ Calculated for C51H61N2O =717.4784; found, 717.4760 (- 3.3 PPM difference).

CT6O2Me.14

Yield: 73 %. m.p. 74 °C, $T_{SmCTBDH\text{-}SmCTBSH}$ 85 °C, $T_{SmCTBSH\text{-}SmA}$ 88 °C, $T_{SmA\text{-}I}$ 163 °C.

^{1}H NMR (400 MHz, CDCl$_3$) δ ppm: 8.66 (s, 1H, CH=N), 8.02 (d, ^3J = 8.7 Hz, 1H, Ar-H), 7.73 (m, appt s, 4H, Ar-H), 7.70 (d, ^3J = 8.6 Hz, 2H, Ar-H), 7.66 (d, ^3J = 8.6 Hz, 2H, Ar-H), 7.55 (d, ^3J = 8.1 Hz, 2H, Ar-H), 7.28 (d, ^3J = 8.1 Hz, 2H, Ar-H), 7.18 (d, ^3J = 8.3 Hz, 2H, Ar-H), 7.11 (d, ^3J = 8.3 Hz, 2H, Ar-H), 6.81 (dd, ^3J = 8.7 Hz, ^4J = 2.6 Hz, 1H, Ar-H), 6.72 (d, ^4J = 2.6 Hz, 1H, Ar-H), 4.00 (t, ^3J = 6.5 Hz, 2H, ArCH$_2$(CH$_2$)$_4$CH$_2$-O), 2.69 (t, ^3J = 7.6 Hz, 2H, ArCH$_2$(CH$_2$)$_4$CH$_2$-O), 2.61 (t, ^3J = 7.4 Hz, 2H, ArCH$_2$(CH$_2$)$_{12}$CH$_3$), 2.54 (s, 3H, ArCH$_3$), 1.81 (m, 2H, Ar(CH$_2$)$_4$CH$_2$CH$_2$-O), 1.71 (p, ^3J = 7.6 Hz, 2H, ArCH$_2$CH$_2$(CH$_2$)$_4$-O), 1.62 (p, ^3J =7.4 Hz, 2H, ArCH$_2$CH$_2$(CH$_2$)$_{11}$CH$_3$), 1.54 (m, 2H, Ar(CH$_2$)$_3$CH$_2$CH$_2$CH$_2$-O), 1.46 (m, 2H, ArCH$_2$CH$_2$CH$_2$(CH$_2$)$_3$-O), 1.26 (m, 22H, ArCH$_2$CH$_2$(CH$_2$)$_{11}$CH$_3$), 0.88 (t, ^3J = 6.9 Hz, 3H, Ar(CH$_2$)$_{13}$CH$_3$). ^{13}C NMR (101 MHz, CDCl$_3$) δ ppm: 161.33, 157.85, 150.73, 145.36, 142.56, 141.64, 140.66, 140.50, 137.78, 137.71, 132.78 (2C), 129.77, 129.20 (2C), 129.16 (2C), 127.76 (2C), 127.71 (2C), 127.68 (2C), 127.42, 127.08 (2C), 120.94 (2C), 119.13, 116.68, 112.59, 110.98, 68.01, 35.64, 35.62, 32.08, 31.77, 31.45, 29.85, 29.84 (2C), 29.83, 29.81, 29.77, 29.69, 29.51, 29.48, 29.25, 29.05, 26.00, 22.85, 19.76, 14.28. IR (νmax/cm^{-1}): 2919, 2846 (C-H, sp^2), 2229 (C≡N stretch), 1607 (para disubstituted benzene), 1591, 1488, 1255, 1002, 812, 502. HRMS: (TOF ESI$^+$) (m/z): [M+H]$^+$ Calculated for $C_{53}H_{65}N_2O$ = 745.5097; found, 745.5070 (- 3.6 PPM difference).

CT6O2Me.16

Yield: 88 %. m.p. 81 °C, $T_{SmCTBDH\text{-}SmCTBSH}$ 88 °C, $T_{SmCTBSH\text{-}SmA}$ 89 °C, $T_{SmA\text{-}I}$ 163 °C.

^{1}H NMR (400 MHz, $CDCl_3$) δ ppm: 8.66 (s, 1H, $\underline{CH}$=N), 8.02 (d, ^{3}J = 8.6 Hz, 1H, Ar-H), 7.73 (m, appt s, 4H, Ar-H), 7.70 (d, ^{3}J = 8.4 Hz, 2H, Ar-H), 7.66 (d, ^{3}J = 8.4 Hz, 2H, Ar-H), 7.55 (d, ^{3}J = 8.2 Hz, 2H, Ar-H), 7.29 (d, ^{3}J = 8.2 Hz, 2H, Ar-H), 7.18 (d, ^{3}J = 8.3 Hz, 2H, Ar-H), 7.11 (d, ^{3}J = 8.3 Hz, 2H, Ar-H), 6.81 (dd, ^{3}J = 8.6 Hz, ^{4}J = 2.5 Hz, 1H, Ar-H), 6.72 (d, ^{4}J = 2.5 Hz, 1H, Ar-H), 4.00 (t, ^{3}J = 6.5 Hz, 2H, $ArCH_2(CH_2)_4\underline{CH_2}$-O), 2.69 (t, ^{3}J = 7.6 Hz, 2H, $Ar\underline{CH_2}(CH_2)_4CH_2$-O), 2.61 (t, ^{3}J = 7.6 Hz, 2H, $Ar\underline{CH_2}(CH_2)_{14}CH_3$), 2.54 (s, 3H, $Ar\underline{CH_3}$), 1.82 (m, 2H, $Ar(CH_2)_4\underline{CH_2}CH_2$-O), 1.71 (p, ^{3}J = 7.6 Hz, 2H, $ArCH_2\underline{CH_2}(CH_2)_4$-O), 1.62 (p, ^{3}J =7.6 Hz, 2H, $ArCH_2\underline{CH_2}(CH_2)_{13}CH_3$), 1.53 (m, 2H, $Ar(CH_2)_3\underline{CH_2}CH_2CH_2$-O), 1.47 (m, 2H, $ArCH_2CH_2\underline{CH_2}(CH_2)_3$-O), 1.26 (m, 26H, $ArCH_2CH_2\underline{(CH_2)_{13}}CH_3$), 0.88 (t, ^{3}J = 6.7 Hz, 3H, $Ar(CH_2)_{15}\underline{CH_3}$). ^{13}C NMR (101 MHz, $CDCl_3$) δ ppm: 161.33, 157.84, 150.73, 145.35, 142.55, 141.63, 140.65, 140.50, 137.77, 137.70, 132.78 (2C), 129.77, 129.20 (2C), 129.15 (2C), 127.75 (2C), 127.71 (2C), 127.67 (2C), 127.42, 127.08 (2C), 120.94 (2C), 119.13, 116.68, 112.59, 110.98, 68.00, 35.64, 35.61, 32.08, 31.77, 31.44, 29.85 (3C), 29.84 (2C), 29.83, 29.81, 29.77, 29.69, 29.51, 29.48, 29.25, 29.05, 26.00, 22.84, 19.76, 14.28. IR (vmax/cm^{-1}): 2917-2847 (C-H, sp^{2}), 2228 (C≡N stretch), 1607 (para disubstituted benzene), 1591, 1488, 1002, 811, 519. HRMS: (TOF ESI^{+}) (m/z): $[M+H]^+$ Calculated for $C_{55}H_{69}N_2O$ = 773.5410; found, 773.5380 (- 3.9 PPM difference).

CT6O2Me.18

Yield: 86 %. m.p. 91 °C, $T_{SmCTBDH\text{-}SmA}$ 90°C, $T_{SmA\text{-}I}$ 163 °C.

^{1}H NMR (400 MHz, $CDCl_3$) δ ppm: 8.66 (s, 1H, $\underline{CH}$=N), 8.02 (d, ^{3}J = 8.6 Hz, 1H, Ar-H), 7.73 (m, appt s, 4H, Ar-H), 7.70 (d, ^{3}J = 8.3 Hz, 2H, Ar-H), 7.66 (d, ^{3}J = 8.3 Hz, 2H, Ar-H), 7.55 (d, ^{3}J = 8.1 Hz, 2H, Ar-H), 7.29 (d, ^{3}J = 8.1 Hz, 2H, Ar-H), 7.18 (d, ^{3}J = 8.2 Hz, 2H, Ar-H), 7.10 (d, ^{3}J = 8.2 Hz, 2H, Ar-H), 6.81 (dd, ^{3}J = 8.6 Hz, ^{4}J = 2.6 Hz, 1H, Ar-H), 6.72 (d, ^{4}J = 2.6 Hz, 1H, Ar-H), 4.00 (t, ^{3}J = 6.5 Hz, 2H, $ArCH_2(CH_2)_4\underline{CH_2}$-O), 2.69 (t, ^{3}J = 7.7 Hz, 2H, $Ar\underline{CH_2}(CH_2)_4CH_2$-O), 2.61 (t, ^{3}J = 7.9 Hz, 2H, $Ar\underline{CH_2}(CH_2)_{16}CH_3$), 2.54 (s, 3H, $Ar\underline{CH_3}$), 1.82 (m, 2H, $Ar(CH_2)_4\underline{CH_2}CH_2$-O), 1.71 (p, ^{3}J = 7.7 Hz,

2H, $ArCH_2\underline{CH_2}(CH_2)_4$-O), 1.61 (m, 2H, $ArCH_2\underline{CH_2}(CH_2)_{15}CH_3$), 1.53 (m, 2H, $Ar(CH_2)_3\underline{CH_2}CH_2CH_2$-O), 1.46 (m, 2H, $ArCH_2CH_2\underline{CH_2}(CH_2)_3$-O), 1.26 (m, 30H, $ArCH_2CH_2\underline{(CH_2)_{15}}CH_3$), 0.88 (t, ^3J = 6.6 Hz, 3H, $Ar(CH_2)_{17}\underline{CH_3}$). ^{13}C NMR (101 MHz, $CDCl_3$) δ ppm: 161.33, 157.85, 150.73, 145.36, 142.56, 141.64, 140.65, 140.50, 137.78, 137.70, 132.78 (2C), 129.77, 129.20 (2C), 129.15 (2C), 127.75 (2C), 127.71 (2C), 127.67 (2C), 127.42, 127.08 (2C), 120.94 (2C), 119.13, 116.68, 112.59, 110.98, 68.00, 35.64, 35.62, 32.08, 31.77, 31.44, 29.85 (5C), 29.84 (2C), 29.83, 29.81, 29.77, 29.69, 29.51, 29.48, 29.25, 29.05, 26.00, 22.85, 19.76, 14.28. IR (νmax/cm^{-1}): 2917, 2848 (C-H, sp^2), 2224 (C≡N stretch), 1604 (para disubstituted benzene), 1590, 1488, 811, 505. HRMS: (TOF ESI^+) (m/z): $[M+H]^+$ Calculated for $C_{57}H_{73}N_2O$ = 801.5723; found, 801.5696 (- 3.4 PPM difference).

2. Additional results

Supplementary Table 2: Phase transition temperatures (°C) and associated entropy changes (in parenthesis, scaled by R) for the CT6O2Me.*m* series.

m	Cr-	N	N_{TB}	SmC_{TB-C}	SmA	SmC_{TB-SH}	SmC_{TB-DH}
1	156 (13.98)	212 (0.19)	122 (4x10^{-3})*				
2	139 (16.82)	202 (0.17)	114 (6x10^{-3})*				
3	134 (11.74)	201 (0.16)	122 (5x10^{-3})*				
4	122 (9.13)	193 (0.12)	115 (5x10^{-3})*	86 (0.44)*			
5	128 (18.04)	191 (0.20)	117 (8x10^{-3})*	75 (0.47)*			
6	100 (13.35)	183 (0.17)	108 (7x10^{-3})*	86 (0.78)*			
7	112 (13.15)	180 (0.14)	110 (5x10^{-3})*	74 (0.42)*			
8	106 (25.44)	174 (0.16)		65 (0.65)*	143 (0.02)	99 (4x10^{-3})*	87 (0.01)*
9	104 (24.73)	173 (0.18)			155 (0.03)	97 (6x10^{-3})*	84 (0.01)*
10	114 (25.15)	167 (0.11)			158 (0.08)	93‡	80‡
11	94 (20.61)	164 (0.11)			158 (0.08)	88 (0.03)*	77 (4x10^{-3})*
12	83 (10.23)	164 (0.38)[a]			162[a]	86 (0.05)*	82 (0.04)*
14	74 (9.20)				163 (0.70)	88 (2x10^{-3})*	85 (2x10^{-3})*
16	81 (8.23)				163 (0.93)	89‡	88 (0.01)*
18	91 (16.75)				163 (1.16)		90 (0.01)*

‡denotes a temperature obtained using POM.

*denotes a temperature obtained from the DSC cooling trace.

[a]denotes peak overlap; quoted entropy change encompasses both N and SmA phases.

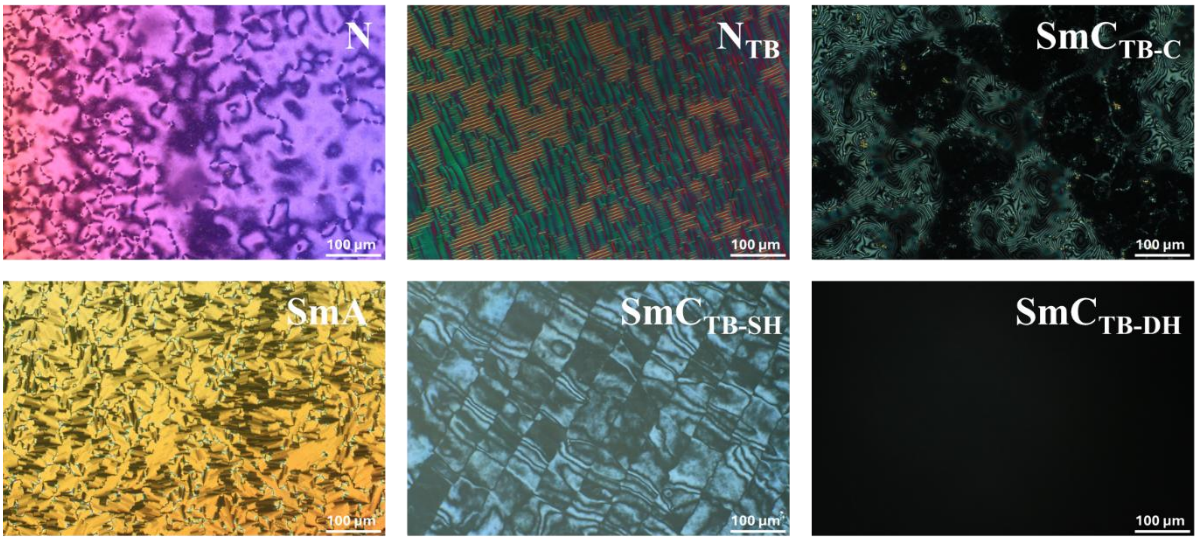

Supplementary Figure 4. Polarizing optical microscope textures of LC phases (a) N phase for $m = 4$, (b) N_{TB} phase for $m = 4$ observed in a homogeneously aligned cell, (c) $SmC_{TB\text{-}C}$ phase for $m = 8$ observed in a homeotropically aligned cell, (d) SmA phase for $m = 9$, (e) $SmC_{TB\text{-}SH}$ phase for $m = 8$ observed in a wedge cell treated for homeotropic anchoring, and (f) $SmC_{TB\text{-}DH}$ phase for $m = 8$ in a homeotropically aligned cell.

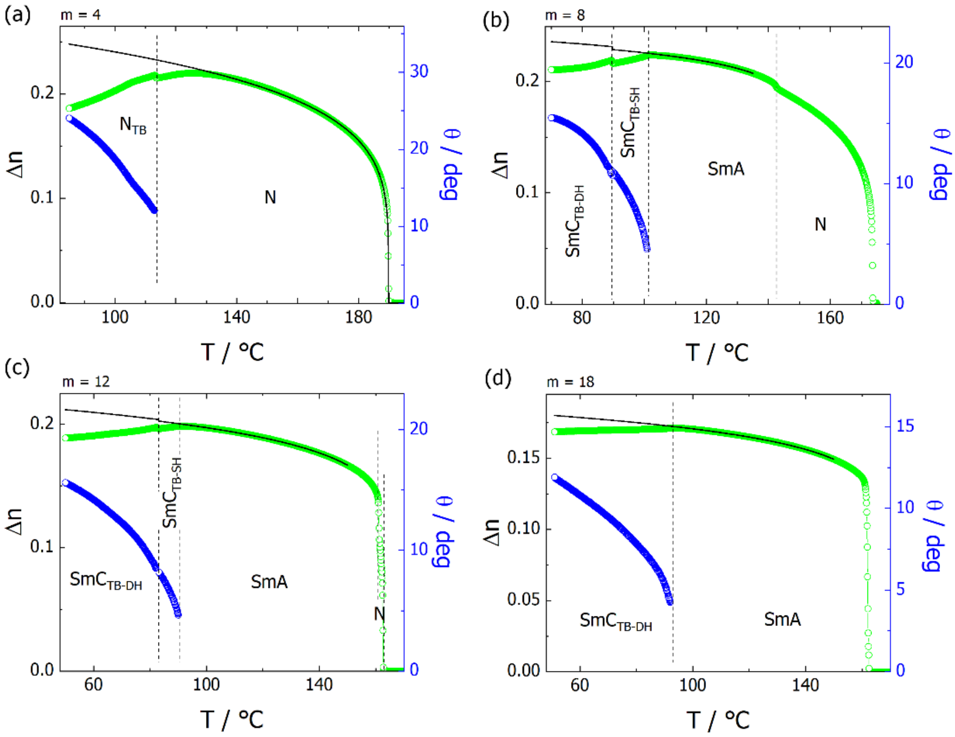

Supplementary Figure 5. Optical birefringence (Δn, green circles) and tilt angle (θ, blue circles) in heliconical phases. Tilt angle was calculated from the deviation of the measured birefringence from the

extrapolated values (black lines, the extrapolation was done assuming a general power-law temperature dependence of birefringence), according to the formula [1]: $\Delta n = \Delta n_0 \left(\frac{3}{2}\cos^2\theta - \frac{1}{2}\right)$, where Δn and Δn_0 are the measured and extrapolated values of birefringence, respectively. Data for the homologues with (a) $m = 4$, (b) $m = 8$, (c) $m = 12$ and

(d) $m = 18$.

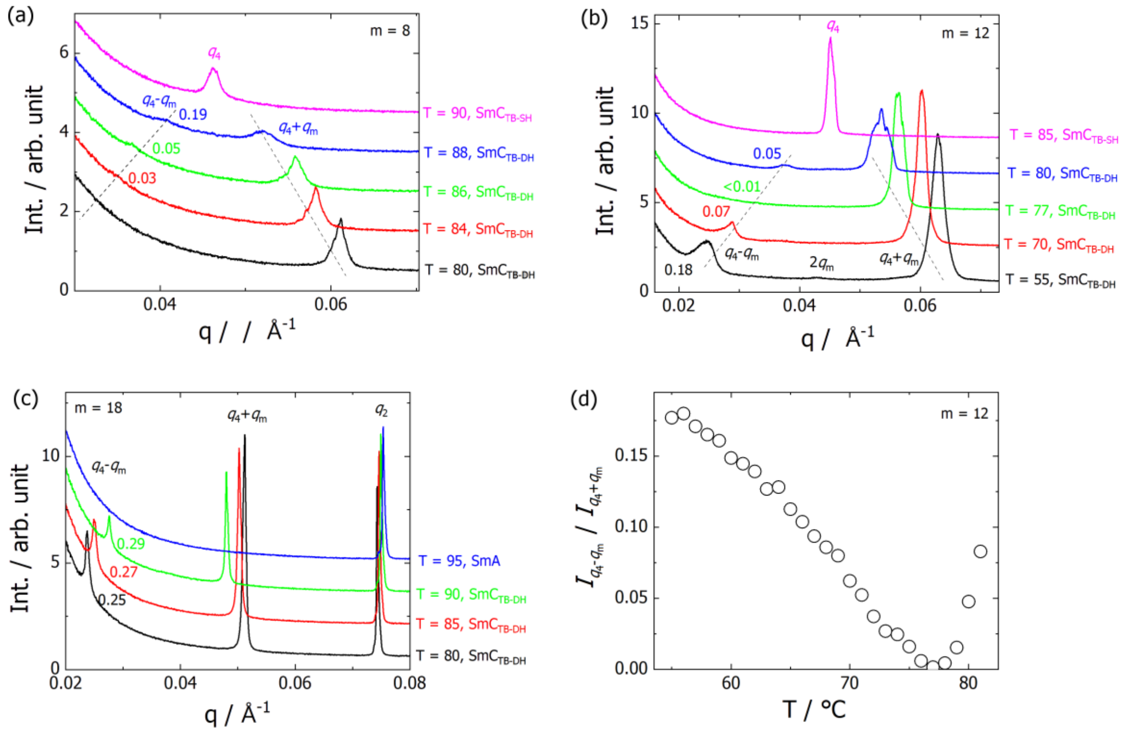

Supplementary Figure 6. Resonant X-ray diffraction data. Intensity of resonant X-ray diffraction signals vs. scattering vector q, recorded at chosen temperatures in SmA, $SmC_{TB\text{-}SH}$ and $SmC_{TB\text{-}DH}$ phases for the homologues with (a) $m = 8$, (b) $m = 12$ and (c) $m = 18$. Curves are vertically shifted for clarity of presentation. (d) Intensity ratio of resonant signals, $I_{q_4-q_m}/I_{q_4+q_m}$, vs. temperature in the $SmC_{TB\text{-}DH}$ phase of the homologue with $m = 12$.

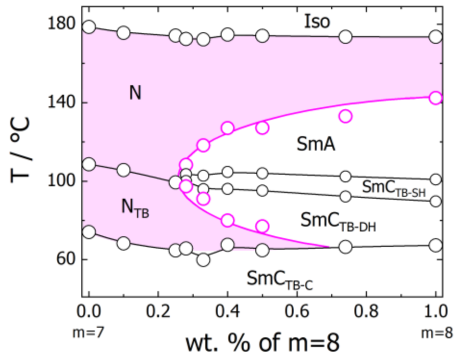

Supplementary Figure 7. Phase diagram for binary mixtures of homologues with $m = 7$ and $m = 8$. Region of the nematic phase is marked by magenta colour. Re-entrant nematic phase is observed in the concentration range between $0.28 - 0.75$ wt. % of the homologue with $m = 8$. The transition temperatures were obtained by analysing optical birefringence changes.

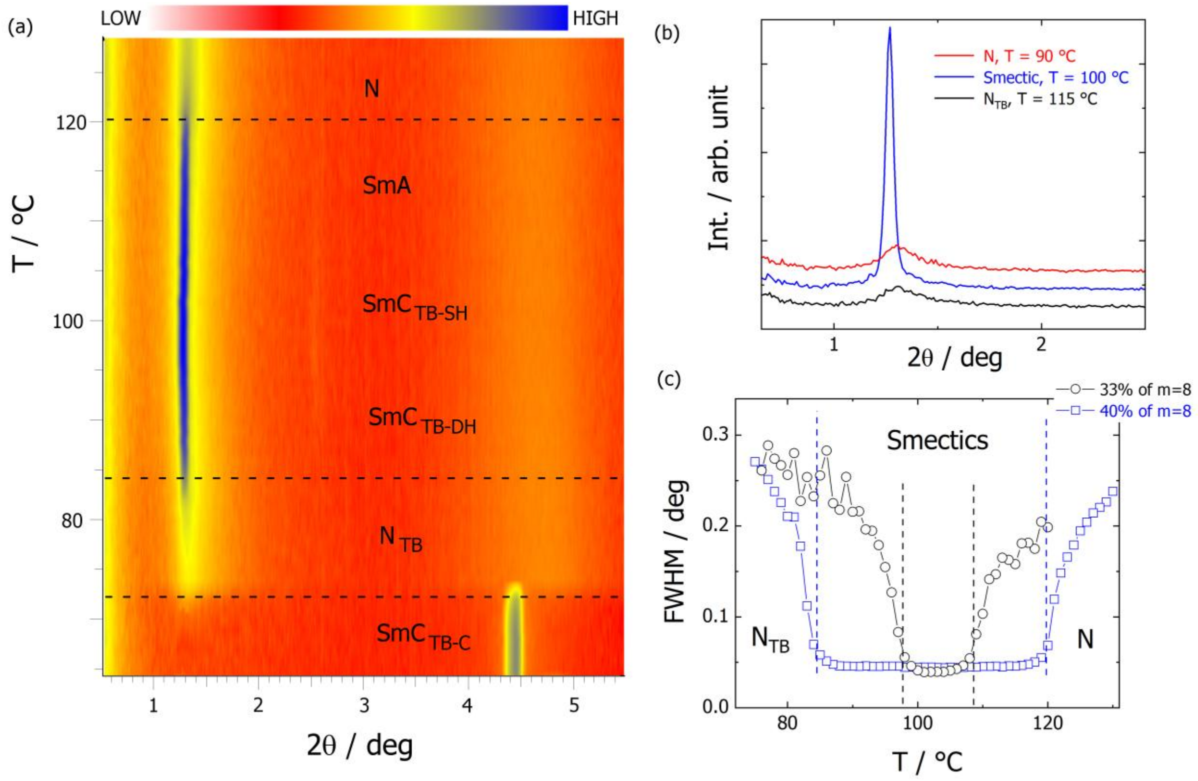

Supplementary Figure 8. X-ray diffraction data for binary mixtures of homologues with $m = 7$ and $m = 8$. (a) Temperature evolution of small-angle XRD signals recorded for a mixture with 33 wt. % of the homologue with $m = 8$, formation of the re-entrant nematic (N_{TB}) phase is marked by broadening of the signal due the loss of the long-range positional order of molecules. Note, that in the $SmC_{TB\text{-}C}$ phase the diffraction

signal corresponds roughly to half the molecular length, indicating intercalated structure of this phase. (b) Small angle X-ray diffractograms recorded in nematic (N), smectic and re-entrant nematic (N_{TB}) phases for a mixture with a 33 wt. % of the homologue with $m = 8$. (c) Temperature dependence of the full width at half maximum (FWHM) of the small-angle diffraction signal corresponding to a bilayer periodicity for mixtures of homologues with $m = 7$ and $m = 8$ (with 33 wt. % of m $= 8$, black circles and with 40 wt. % of $m = 8$, blue squares), note that in both, nematic and re-entrant N_{TB} phases, the FWHM is considerably higher than in the smectic phase (in smectic phases it is defined by instrumental resolution).

3. Theoretical modelling

In modelling the response in resonant x-ray scattering, we follow the procedure, presented in detail in the Supplementary Information (SI) to [2]. Here, we briefly outline the main steps. The dimer is modelled by placing one resonant dipole with a uniaxial polarizability in each "arm" of the dimer. The tensor form factor in the eigensystem of the dipole ($\mathbf{F}_{ei}$), where the local z-axis is aligned with the arm of the dimer, is [3]:

$$\mathbf{F}_{ei} = f_0 \begin{pmatrix} 1 & 0 & 0 \\ 0 & 1 & 0 \\ 0 & 0 & -2 \end{pmatrix}. \tag{1}$$

The parameter f_0 depends on the scattering strength. In resonant scattering at the carbon K-edge, several atoms in the molecule respond, and f_0 is expected to be much larger than in case when there is only a single resonant atom incorporated into the molecule.

The tensor form factor in the laboratory coordinate system is obtained through the following set of rotations of $\mathbf{F}_{ei}$ (see Supplementary Figure 6a):

- a rotation by angle α (lower arm of the dimer) or $-\alpha$ (upper arm of the dimer) about the x-axis;
- a rotation by angle θ about the y-axis;
- a rotation by angle φ about the z-axis.

The tensor form factor ($\mathbf{F}$) in the laboratory system is obtained as:

$$\mathbf{F} = \mathbf{R}_{\varphi}\mathbf{R}_{\theta}\mathbf{R}_{\alpha}\mathbf{F}_{ei}\mathbf{R}_{\alpha}^{-1}\mathbf{R}_{\theta}^{-1}\mathbf{R}_{\varphi}^{-1} \;, \tag{2}$$

Where $\mathbf{R}_{\alpha}$, $\mathbf{R}_{\varphi}$, and $\mathbf{R}_{\theta}$ are rotation matrices:

$$\mathbf{R}_{\alpha} = \begin{pmatrix} 1 & 0 & 0 \\ 0 & \cos\alpha & \sin\alpha \\ 0 & -\sin\alpha & \cos\alpha \end{pmatrix} \;, \tag{3}$$

$$\mathbf{R}_{\varphi} = \begin{pmatrix} \cos\varphi & -\sin\varphi & 0 \\ \sin\varphi & \cos\varphi & 0 \\ 0 & 0 & 1 \end{pmatrix} \;, \tag{4}$$

and

$$\mathbf{R}_{\theta} = \begin{pmatrix} \cos\theta & 0 & \sin\theta \\ 0 & 1 & 0 \\ -\sin\theta & 0 & \cos\theta \end{pmatrix} \;. \tag{5}$$

With the chosen set of rotation matrices, a positive angle α for the lower arm of the dipole and a negative α for its upper arm, together with positive θ and φ, describe the dimer with the apex oriented in the direction perpendicular to the plane defined by the vectors $\hat{z}$ and $\vec{n}$ ($\vec{n}$ connects the ends of the dimer, see Supplementary Figure 6a), and pointing in the direction of helix rotation.

The tensor form factor of the ferri-like 4-layer structure (Supplementary Figure 6) is obtained by summing the form factors given in equation (2), taking into account the orientation of the dimer axis (direction of $\vec{n}$) in each smectic layer and considering the phase difference due to scattering from different layers, each of thickness d_0. The scattering amplitude tensor (**A**) is obtained as a combination of the form factor corresponding to the four-layer structure and the structure factor of the four-layer repeating unit. The summation over a very large (effectively infinite) number of layers leads to delta functions, and the tensor elements are non-zero only for specific values of the scattering vector (for details, see SI to [2]).

The magnitude of the scattering vector is expressed as

$$q = \frac{2\pi}{d_0} h \;, \tag{6}$$

where h is the Miller index. If $\varepsilon = 0$, the basic periodicity is $4d_0$, thus one expects interference peaks at h being equal to multiples of $1/4$. When $\varepsilon \neq 0$, these peaks split. Tensor elements are different from zero only for $\pm 8\varepsilon + 8\pi h = 2\pi i$ and $\pm 4\varepsilon + 8\pi h = 2\pi i$, where i is an integer, which means:

$$h = \frac{i}{4} \pm \frac{\varepsilon}{\pi} \tag{7}$$

and

$$h = \frac{i}{4} \pm \frac{\varepsilon}{2\pi} \quad . \tag{8}$$

We are interested in the integer i being $0, 1$ or 2. The magnitudes of the scattering vectors related to the allowed peaks are denoted by $q_2 \pm q_m$, $q_2 \pm 2q_m$, $q_4 \pm q_m$, $q_4 \pm 2q_m$, q_m and $2q_m$, where $q_4 = \pi/(2d_0)$, $q_2 = \pi/d_0$ and $q_m = \varepsilon/d_0$.

The scattering amplitude tensor $\underline{A}$ can be expressed in a general form as:

$$\underline{A} = \begin{pmatrix} f_{11} & f_{12} & f_{13} \\ f_{21} & f_{22} & f_{23} \\ f_{31} & f_{32} & f_{33} \end{pmatrix} \quad . \tag{9}$$

For a ferri-like four layer structure with an additional superimposed helix, the tensor elements f_{11}, f_{22}, f_{12} and f_{21} are different from zero at $h = \frac{i}{4} \pm \frac{\varepsilon}{\pi}$, f_{11} and f_{22} also at $h = \frac{i}{4}$, f_{33} only at $h = \frac{i}{4}$, while f_{13}, f_{31}, f_{23} and f_{32} are different from zero only at $h = \frac{i}{4} \pm \frac{\varepsilon}{2\pi}$.

After constructing the scattering amplitude tensor, the intensity of the peak at a given value of q is obtained by following the procedure introduced by Dmitrienko [4], as described in detail in the SI of [2]. The intensity of the scattered light is calculated for the unpolarised incident light. The scattered light is polarised either within the scattering plane (π-polarization) or perpendicular to it (σ-polarization), and the two contributions are summed.

We are interested in the intensities of the peaks corresponding to scattering vector magnitudes $2q_m$, $q_4 \pm q_m$ and $q_2 - 2q_m$. The intensities depend on the angles α, θ, δ and ε. Only α is expected to be temperature independent. The temperature variation of θ for the studied materials is shown in

Supplementary Figure 2. In the double helix phase (SmC$_{\text{TB-DH}}$), we approximated it by a square-root dependence as:

$$\theta = \theta_0 + k_\theta \sqrt{T_0 - T} \ , \tag{10}$$

where T_0 is the transition temperature from the SmC$_{\text{TB-SH}}$ to the SmC$_{\text{TB-DH}}$ phase, θ_0 is the tilt angle at the transition temperature, and k_θ is a coefficient. The value of ε at a given temperature is obtained from the measured magnitude of the scattering vector in the split q_4 peak (see Figure 4), as $\varepsilon = q_m/d_0$. Close to the phase-transition temperature, the dependence $\varepsilon(T)$ can also be approximated by a square-root dependence:

$$\varepsilon = \varepsilon_0 + k_\varepsilon \sqrt{T_0 - T} \ , \tag{11}$$

where ε_0 is the value at the phase-transition temperature and k_ε is a coefficient estimated from the measured data. We note that this temperature dependence of ε (equation (11)) yields values of ε that are too large far from the phase transition temperature. However, as our aim is only to capture the qualitative characteristics of the experimental results, this is not problematic. The main uncertainty concerns the temperature variation of the angle δ. We know that δ should be far from $\pi/2$ in the single-helix phase, where biaxiality is significant (see Supplementary Figure 2). From previous studies [1,2], however, we also know that $\delta \approx \pi/2$ at the temperature at which the peak at $q_4 - q_m$ disappears. Thus, δ should increase with decreasing temperature, and for this angle we also employed a square-root dependence on temperature:

$$\delta = \delta_0 + k_\delta \sqrt{T_0 - T} \ . \tag{12}$$

The parameters used to model intensities of the resonant peaks for materials with $m = 8, 12$ and 18 are given in Supplementary Table 3. The parameters were chosen such that qualitative agreement with the measured intensity ratios $I_{q_4-q_m}/\ I_{q_4+q_m}$ was obtained, while the ratio $I_{q_2-2q_m}/\ I_{q_4+q_m}$ remained very small (as this peak is not observed in any of the materials). For the material with $m = 12$, the ratio $I_{2q_m}/\ I_{q_4+q_m}$should be small, but larger than $I_{q_2-2q_m}/\ I_{q_4+q_m}$, since a very weak but distinct peak at $2q_m$ was observed in this material. The temperature dependence of the intensity ratios for the

chosen set of parameters is shown in the main text. Here, we illustrate how the intensities change with variation of the parameters listed in Supplementary Table 3.

The effect of varying δ_0, k_δ and α for materials with $m = 8, 12$ and 18 is shown in Supplementary Figures 7, 8 and 9. Unless stated otherwise, the parameter values given in Supplementary Table 3 are used in all the figures. We find that the largest effect on the intensity ratios arises from variation of α. The greatest influence is observed for the ratios $I_{2q_m}/\ I_{q_4+q_m}$ and $I_{q_2-2q_m}/\ I_{q_4+q_m}$; however, for materials with $m = 8$ and 18, the intensities of the peaks at $2q_m$ and $q_2 - 2q_m$ are very low, below the detection limit. By inspecting the effect of variation of α on the intensity of these two peaks for the material with $m = 12$, we can determine the sign of α, as it is evident that a positive value of α would render the peak at $q_2 - 2q_m$ larger than that at $2q_m$. Conversely, for material with $m = 18$, α is approximately zero, since the correct temperature variation of the intensity ratio $I_{q_4-q_m}/\ I_{q_4+q_m}$ (which should decrease slightly with decreasing temperature) can be obtained only by $\alpha \approx 0$. Even a slight bend of the dimer ($\alpha = \pm 0.05$) produces a temperature dependence that disagrees with the experimental observations.

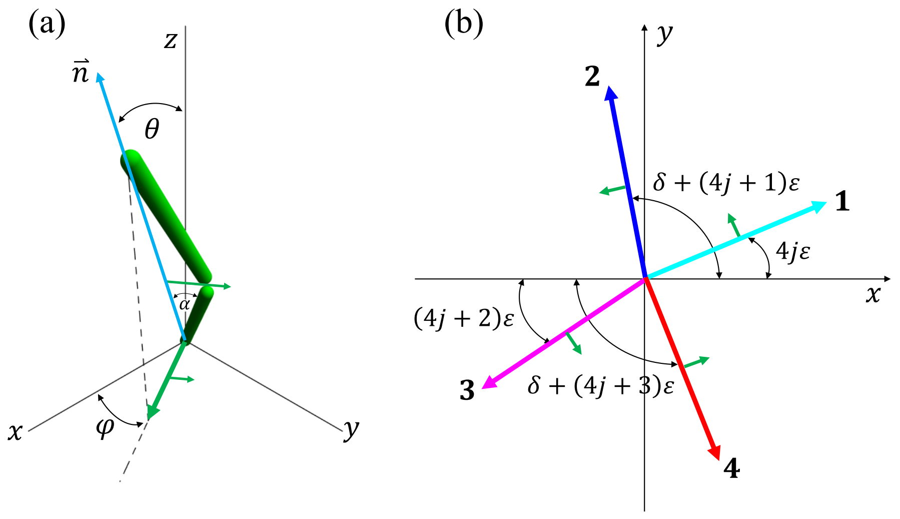

Supplementary Figure 9. (a) The orientation of the long dimer axis (vector $\vec{n}$) in the laboratory coordinate system is defined by the tilt angle θ and the azimuthal angle φ. The apex angle of the dimer is π − 2α. The apex of the dimer points in the direction perpendicular to the plane defined by $\vec{n}$ and $\hat{z}$. The green arrows in the xy-

plane show the projection of the vector n⃗ onto the xy-plane and the direction of the dimer apex. (b) In the four-layer structure, the molecules in successive layers differ in the orientation of n⃗. The figure shows the projection of n⃗ on the smectic plane (xy-plane) in the j-th stack of four successive layers. The structure is ferri-like the angle between projections of n⃗ in layers 1 and 2 and layers 3 and 4 is δ, while the angle between layers 1 and 3 and 2 and 4 is π. An additional rotation by an angle ε is superimposed on this "basic" ferri-like structure.

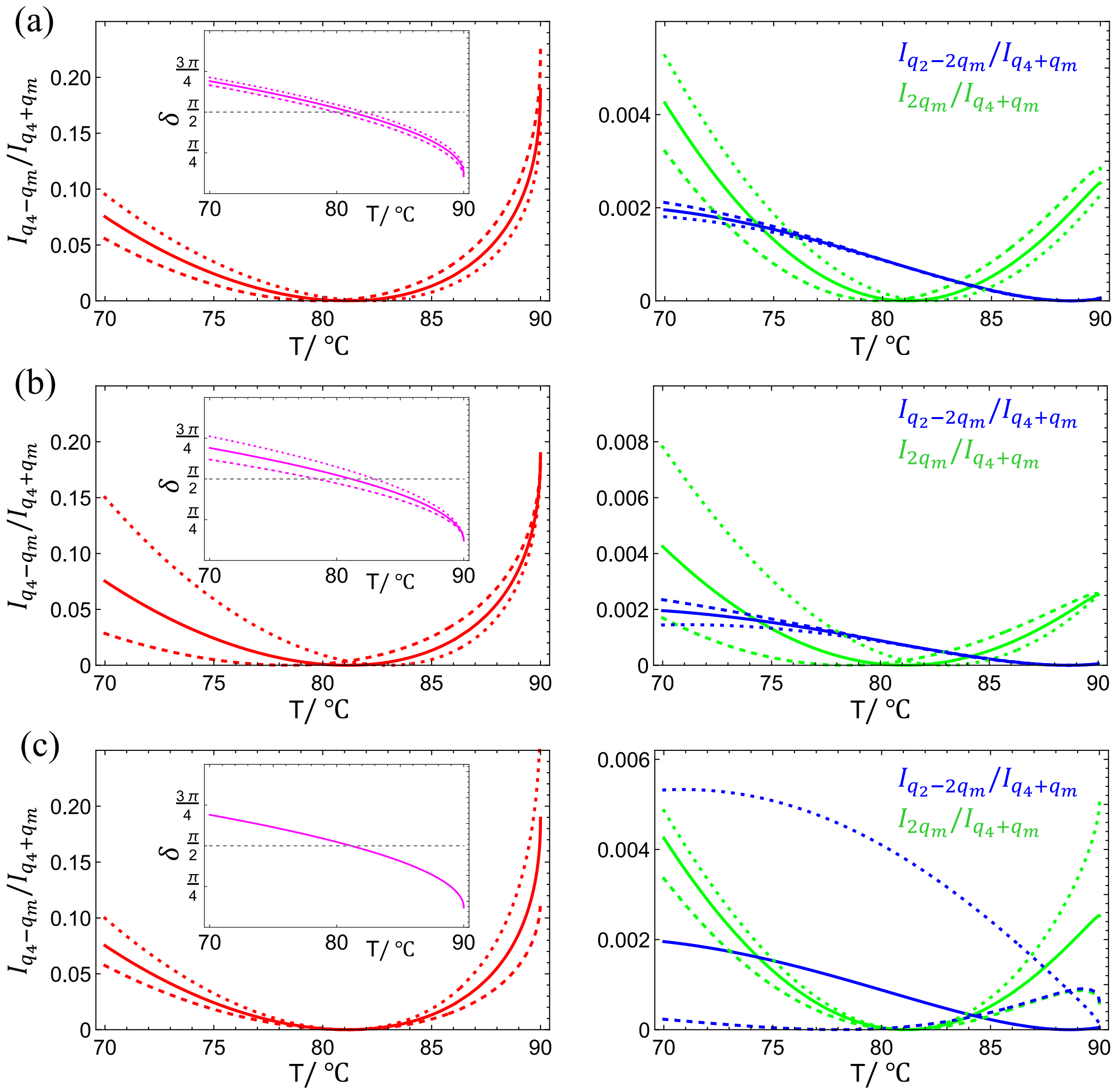

Supplementary Figure 10. Effect of parameter variation on the RSoXS signals intensity ratios for the material with $m = 8$. (a) Variation of δ_0; $\delta_0 = 0.38$ (solid lines), $\delta_0 = 0.30$ (dashed lines), $\delta_0 = 0.45$ (dotted lines). (b) Variation of k_δ; $k_\delta = 0.40\ K^{-1/2}$ (solid lines), $k_\delta = 0.35\ K^{-1/2}$ (dashed lines), $k_\delta = 0.45\ K^{-1/2}$ (dotted lines). (c) Variation of α; $\alpha = -0.10$ (solid lines), $\alpha = -0.15$ (dashed lines), $\alpha = -0.05$ (dotted lines).

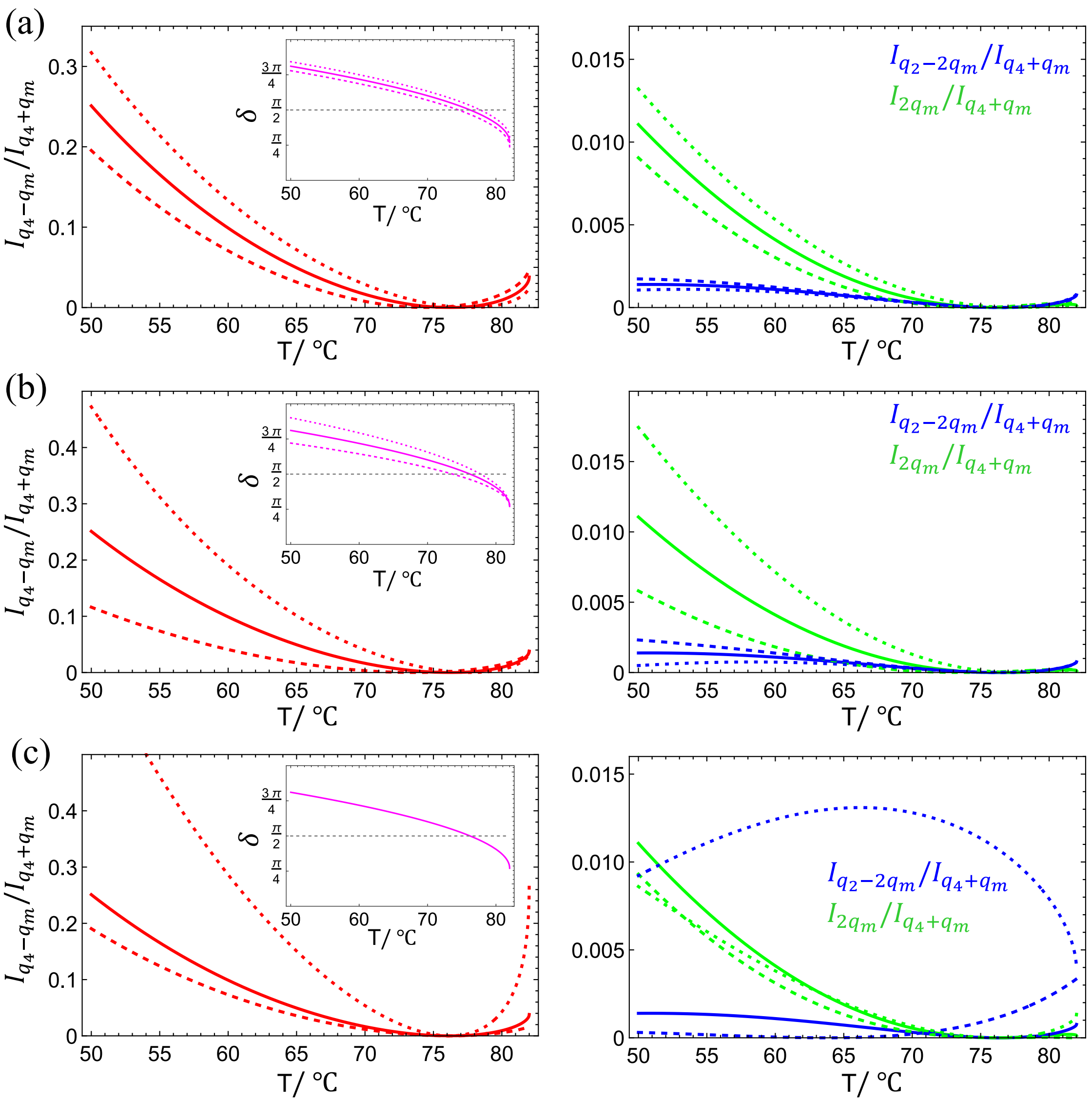

Supplementary Figure 11. Effect of parameter variation on the RSoXS signals intensity ratios for the material with $m = 12$. (a) Variation of δ_0; $\delta_0 = 0.85$ (solid lines), $\delta_0 = 0.75$ (dashed lines), $\delta_0 = 0.95$ (dotted lines). (b) Variation of k_δ; $k_\delta = 0.30\ \text{K}^{-1/2}$ (solid lines), $k_\delta = 0.25\ \text{K}^{-1/2}$ (dashed lines), $k_\delta = 0.35\ \text{K}^{-1/2}$ (dotted lines). (c) Variation of α; $\alpha = -0.10$ (solid lines), $\alpha = -0.15$ (dashed lines), $\alpha = +0.05$ (dotted lines).

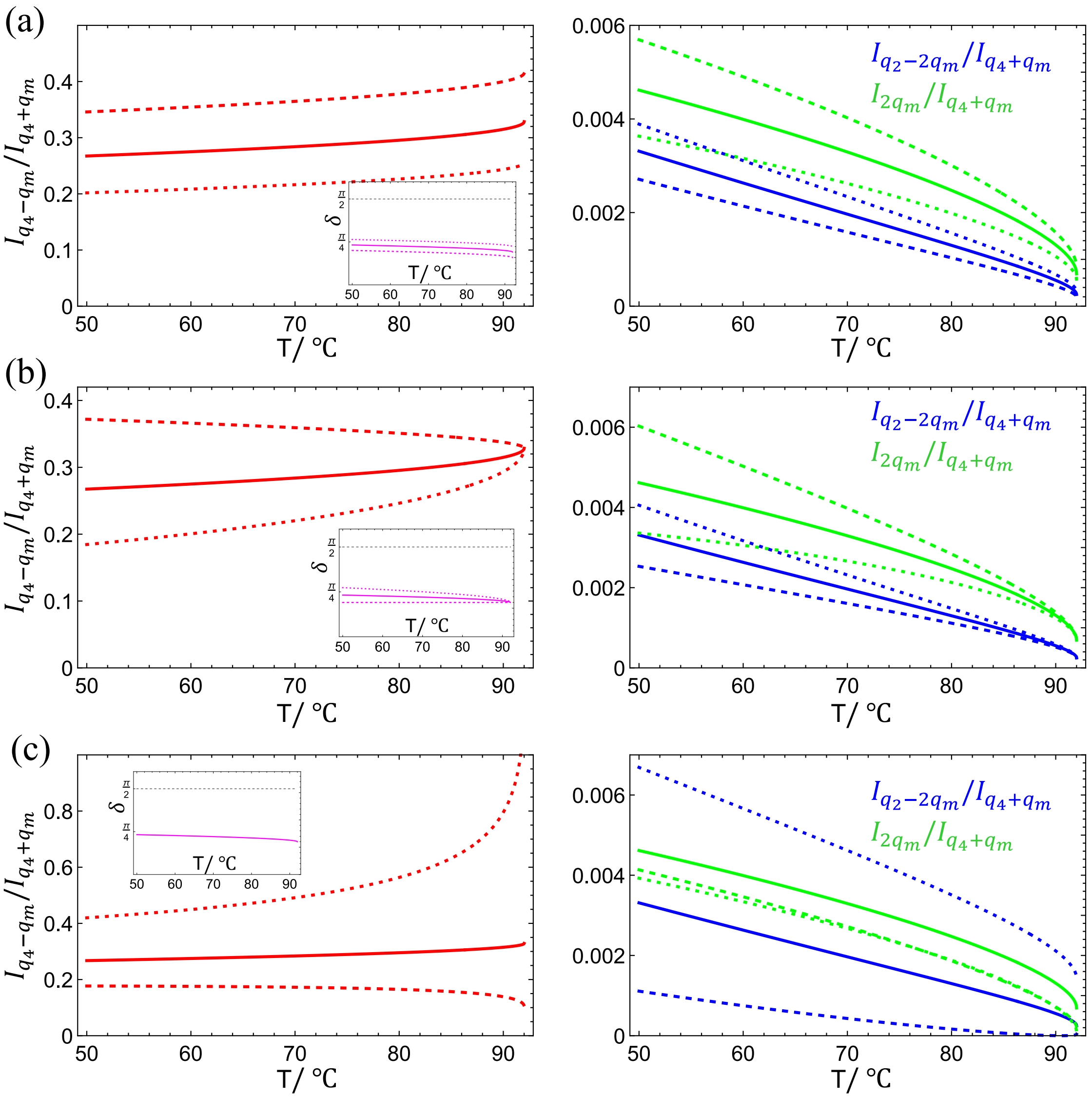

Supplementary Figure 13. Effect of parameter variation on the RSoXS signals intensity ratios for the material with $m = 18$. (a) Variation of δ_0; $\delta_0 = 0.60$ (solid lines), $\delta_0 = 0.50$ (dashed lines), $\delta_0 = 0.70$ (dotted lines). (b) Variation of k_δ; $k_\delta = 0.02\ \mathrm{K}^{-1/2}$ (solid lines), $k_\delta = 0\ \mathrm{K}^{-1/2}$ (dashed lines), $k_\delta = 0.04\ \mathrm{K}^{-1/2}$ (dotted lines). (c) Variation of α; $\alpha = 0$ (solid lines), $\alpha = -0.05$ (dashed lines), $\alpha = +0.05$ (dotted lines).

Supplementary Table 3. Model parameters. Parameters used to model the intensity ratios of the resonant peaks for materials with $m = 8, 12$ and 18. For a description of the parameters, see the text accompanying eqs. (10). (11) and (12).

m	θ_0	k_θ [K$^{-1/2}$]	ε_0	k_ε [K$^{-1/2}$]	δ_0	k_δ [K$^{-1/2}$]	T_0 [°C]	α
8	0.19	0.020	0	0.16	0.38	0.40	90	−0.10
12	0.13	0.023	0	0.18	0.85	0.30	82	−0.10
18	0.07	0.020	0.34	0.039	0.60	0.020	92	0

Supplementary References

[1] C. Meyer, G.R. Luckhurst, I. Dozov, The temperature dependence of the heliconical tilt angle in the twist-bend nematic phase of the odd dimer CB7CB, J. Mater. Chem. C. 3 (2015) 318–328. https://doi.org/10.1039/c4tc01927j. [Ref. 33 in main text]

[2] M. Salamończyk, N. Vaupotič, D. Pociecha, R. Walker, J. M. D. Storey, C. T. Imrie, C. Wang, C. Zhu, E. Górecka, Multi-level chirality in liquid crystals formed by achiral molecules, Nat. Commun. 10, 1922 (2019). DOI: 10.1038/s41467-019-09862-y [Ref. 17 in main text]

[3] A.-M. Levelut, B. Pansu, Tensorial X-ray structure factor in smectic liquid crystals, Phys. Rev. E 60, 6803–6815 (1999). DOI: 10.1103/PhysRevE.60.6803. [Ref. 26 in main text]

[4] V. E. Dmitrienko, Forbidden reflections due to anisotropic X-ray susceptibility of crystals, *Acta Crystallogr. Sect. A* **39**, 29-35 (1983).
