# Supplementary material for: Diverse helical structures made of achiral mesogenic dimers
Source: Nat Commun. 2026 May 6;17:6090. doi: 10.1038/s41467-026-72565-8 (PMC13357721; doi:10.1038/s41467-026-72565-8)
Supplement: Supplementary file 2 — Transparent Peer Review file [file 41467_2026_72565_MOESM2_ESM.pdf]

nature portfolio

Peer Review File

Diverse helical structures made of achiral mesogenic dimers

Corresponding Author: Dr Rebecca Walker

This file contains all reviewer reports in order by version, followed by all author rebuttals in order by version.

Version 0:

Reviewer comments:

Reviewer #1

(Remarks to the Author)
The manuscript presents a structural investigation of heliconical smectic phases in a homologous series of achiral dimers, a combination of polarized optical microscopy (POM), differential scanning calorimetry (DSC), X-ray diffraction (XRD), and resonant soft X-ray scattering (RSoXS), with complementary circular dichroism (CD) and AFM measurements on selected compounds. The identification of a previously unreported SmCTB-C phase and the modeling of double-helical arrangements are notable. However, the manuscript in its current form lacks sufficient interpretive depth, mechanistic analysis, and comparative context. Overall, the work presents promising structural observations, but major revisions are needed to clarify its contributions, strengthen the interpretation, and improve the manuscript's overall coherence.
Below are specific points that should be addressed.
1. The suggestion that a terminal methyl group induces helicity is not supported by references or comparative data. This should either be framed as a hypothesis or substantiated with prior examples.
2. The reported transitions between 4-layer, 3-layer, and long-pitch heliconical structures are presented descriptively, but the physical basis for these transitions is not explored. There is no discussion of possible steric, elastic, or packing-related mechanisms.
3. The stabilization of the SmCTB–C phase is not explained. A more mechanistic perspective even if speculative would significantly improve the interpretive value of the work.
4. Although relevant literature is cited, there is little direct comparison with prior studies involving heliconical phases or RSoXS analyses. A more structured discussion of how the present findings differ from existing work would help clarify the contribution.
5. The manuscript lacks a dedicated Discussion section. Interpretations are embedded within the Results and remain largely descriptive. A standalone section synthesizing the broader significance of the findings would be beneficial.
6. The overall structure would benefit from clearer sectioning, improved integration of comparative and mechanistic content, and a more substantive Conclusion.

Reviewer #2

(Remarks to the Author)
The paper describes the chemical synthesis and a thorough physical characterization including birefringence, small-angle and resonant x-ray scattering of a homolog series of flexible bent-shape molecules. Depending on terminal chain length and temperature, it is found that the materials form a twist-bend nematic (NTB) phase and several helical smectic phases labeled as SmCTB–SH, SmCTB–DH and SmCTB–C phase featuring wide-range helical pitches. Based on the abstract, the punchline of the paper appears to be the exceptional diversity of spontaneously chiral phases formed by achiral mesogens.

The material synthesis and the physical characterization are professionally and carefully carried out. However, I have several issues with the claim of "exceptional diversity", with the arguments that are likely understandable only for a very narrow group of experts in the field of bent-shape dimers, and consequently with the limited interest the results will attract.

1. Spontaneously chiral phases with wide-range of ambidextrous helical pitches are well-known in the B2, B4 and B7 phases of rigid-bent-core molecules (some materials have all these phases). Interestingly the authors only mention the B7 phase that form micron-scale pitch even when the molecule is achiral. They, however, completely ignore the case of the B4 materials that also form helical structures ranging from 0.1-1 µm pitch in form of helical nanofilaments (L. E. Hough et al, Science 2009 , 325 , 452) which can also have a secondary helix (Zhang et al, Nat. Commun.2014 , 5 , 3302) similar to that

of the SmCTB–DH mentioned here. Additionally, with variation of the molecular structures, more variants such as modulated helical nanofilament phase (HNFmod) and even helical microfilament phases have been observed by Tsai et al (Angew. Chem. Int.Ed. 2013 , 52 , 5254) and Li et al (Nat. Commun. 2018, 9, 714). Therefore, it looks necessary to me that the authors compare their helical phases of flexible bent-core molecules with the helical B4 phases of rigid bent-core molecules.

2. The origin of the naming of the SmCTB–SH, SmCTB–DH and SmCTB–C phases is unclear (I assume SH stands for single helix and DH means double helix, but I am unsure what TB-C means) and indicate subtle differences even if they are distinct as the authors claim. The meaning of angles δ and ε introduced in line 198, and the adopted temperature dependences of ε and the tilt angle θ are unclear. I assume if someone were familiar with all the previous papers of the authors on these systems, they were clear, but I think they should be understandable also for people reading only this paper.

3. Some statements in the conclusions, such as “deformations of the director field, effectively rendering negative values of the bend elastic constant” and “molecular diffusion between adjacent layers (an entropic effect) destabilises this structure and favours tilting of molecules in a single plane” requires more explanation and at least references to be able to follow them.

4. The description of the birefringence in the Supporting Information is inadequate. From the description “1f and 2f components to yield a retardation induced by the sample. Knowing the sample thickness, the retardation was recalculated into optical birefringence” it is not clear how they measured the birefringence. At least a reference where this technique was described in detail is necessary.

In summary, the presented work is likely correct and valid, but the presentation makes it understandable only for a very small number of experts who are completely familiar with the previous work of the authors in this subject. Also, a discussion about similarity and differences of helical systems in achiral rigid bent-core systems seems to be necessary. It is up to the editor to decide whether such presentation is publishable in Nature Communication or not.

Reviewer #3

(Remarks to the Author)
This contribution reports a series of heliconical smectics and nematic liquid crystalline (LC) phases for a homologous series of newly synthesized non-symmetric dimers formed by two different rod-like units interconnected by an odd-numbered hexamethyleneoxy spacer.
The synthesis of the compounds is reported in sufficient detail and the required analytical data (1H, 13C-NMR, IR, HR-MS) are provided in the SI.
In the series of compounds, a chain length and temperature dependent transitions between nematic, twist-bend nematic (NTB) and different heliconical tilted smectics LC phases (SmTB) were observed for the synthesized achiral compounds. The Investigation of the LC phases was conducted by POM, DSC and XRD. The structures of the distinct heliconical smectic phases were further investigated by RSoXS, AFM and CD. This confirms the presence of a primary and development of a secondary helix with a complex dependence on molecular structure and temperature (i.e. degree of intercalation, tilt, molecular helicity/conformation). In addition, a reentrant NTB phase was observed at the transition between intercalated and non-intercalated bilayer SmTB phases.
Overall, this is an interesting work with significance for the understanding of spontaneous development of chirality in soft matter systems and providing insights into the relations between different modes of heliconical organization. The work is sound, appropriate research methods have been used and the data are convincingly interpreted, and the conclusions are properly evidenced by experimental data. The paper is considered as appropriate with respect to content and significance for Nat Comm.
However, the following points need consideration before publication:
p.2, line ~80: the methyl group is also expected to have an influence on the molecular conformation of the Schiff base unit, thus favoring helical conformations and supporting helix formation.
P. 2, line89: I would suggest to show the local structure in N/NTB (intercalated with d = L) in the SI for more clarity (similar to Fig. 1e,f).
p.3 (Fig. 1f) why is structure (f) assigned as partly interdigitated bilayer, at least in the shown figure there is only a minimal overlapping between the CH3 groups at the ends (if the figure would be in scale, no numerical molecular parameters are given). In this case the small difference could easily be explained by conformational disorder and the orientational order parameter being <1 in a (non-intercalated) bilayer structure? (also the tilt contributes to the slight reduction of d). For a “partly interdigitated bilayer phase” (“SmXd”) a much lager difference between d and 2L of around d = 0 1.4-1.8 Lmol is usually found. A graphic or table comparing molecular lengths with d-values or d/L values in the SI would be helpful. It would also be easier for the reader if an intercalated and a non-intercalated bilayer smectics phases would be distinguished.
p.3 line 91: Why is the intercalated bilayer structure named SmCTB-C? what is the meaning of “C” here? Why not, for example, SmCTB-1/2 ?
p. 5, Fig. 3a: there is no visible change of d at the SmA-SmC transition which would suggest that SmA is in fact at tilt-

randomized SmC phase (similar winding/unwinding process as at the N-NTB transition). Could this be commented?
p. 5: d0 is the single molecular layer (= d1/2), it would be helpful to also indicate this distance d0 in Fig 1f.
In order to make this paper more accessible to readers from a broader field of mirror symmetry breaking and helical self-assembly, I would suggest to add at the end of the conclusion one or two sentence outlining the importance of the obtained results with a broader view.
Moreover, in the introduction: On p2 lines 51ff, during the discussion of chirality by achiral molecules in fluid systems, it should be noted that even in Isotropic liquids spontaneous chirality/helicity, (though in this case with helical instead of heliconical structure) has been observed (Dressel et al. Nat. Chem. 2014, 6, 971). Moreover, it should be pointed out (lines 57/58) that the first example of heliconical smectic phases of achiral molecules (in this case a polar version of SmTB with incommensurate 4-layer structure) was discovered for achiral bent-core mesogens (Sreenilayam et al. Nat Commun. 2016, 7, 11369). This work has appeared 2 years before the authors own work with dimesogens (ref. 13).
SI: The original references for the used synthesis procedures and the sources of the starting materials should be given. Are 6-bromo-1-[4-(4-bromophenyl)phenyl]hexan-1-one, 1-bromo-4-[4-(6-bromohexyl)phenyl]benzene, 4-[4-[4-(6-bromohexyl)phenyl]phenyl]benzonitrile, new compounds? Otherwise please give literature refences to previous synthesis. Please also add original 1H and 13C NMR spectra for at least one representative of the final compounds.

Version 1:

Reviewer comments:

Reviewer #1

(Remarks to the Author)
The revised manuscript is clearer, better structured, and substantially improved compared with the original submission. The authors now provide a more careful hypothesis rather than an overly speculative mechanistic interpretation, making the overall argument more coherent and convincing. In particular, the additional mechanistic discussion, the expanded comparison with prior studies, and the inclusion of a dedicated Discussion section have strengthened the paper. Most of my original concerns have been addressed. Although direct evidence that the mechanism of the evolution of four-layer distorted clock-like heliconical arrangement toward eventually anticlinic bilayer structure is still lacking, the revised manuscript presents a more convincing mechanistic framework in which the chiral structure is interpreted as arising from the competition between negative bend elasticity, which favors heliconical ordering, and steric-entropic interlayer diffusion effects, which favor in-plane ordering. The added references sufficiently support this general framework, although they do not uniquely establish the specific structural evolution proposed for the present system. With this remaining concern in mind, obtaining more direct evidence to substantiate the proposed mechanism would be an important direction for future work.

Point-to-point assessment is as follows:
1. Original concern: The suggestion that a terminal methyl group induces helicity was not sufficiently supported by references or comparative data.
Assessment of revision:
This point has been addressed satisfactorily. The authors have now introduced a comparison with the analogous CT6O.m series and clarified that the role of the methyl substituent is not to directly “induce helicity,” but rather to reduce the tendency to form more highly ordered smectic phases, thereby allowing the heliconical NTB phase to emerge more readily. The logical chain here is more defensible than original claim.
2. Original concern: The manuscript described these transitions phenomenologically, but did not discuss possible steric, elastic, or packing-related mechanisms.
Assessment of revision:
The author acknowledges the niche of the theoretical model for these multi-helical smectic structures, and the explanation remains speculative. This clarification is appreciated and current interpretation is literature supported by added references. However, the proposed pathway from a distorted four-layer structure toward a ultimately toward a bilayer anticline tendency is still not directly demonstrated, which could be the future direction for authors to explore more.
This concern has been partially resolved. The mechanism discussion is improved and acceptable, but it could be stronger if more direct evidence could be provided.
3. Original concern: The stabilization of the SmCTB-C phase was not explained, and even a speculative mechanistic interpretation would improve the paper.
Assessment of revision:
This point has been addressed well in current version.
4. Original concern: The original manuscript did not sufficiently position the present work relative to prior studies on heliconical phases and resonant soft X-ray scattering.
Assessment of revision:
This concern has been addressed sufficiently. The revised version added relevant reference to earlier RSoXS work on helical structures in rod-like mesogens, NTB phase, and bent-core smectic systems, the novelty of present work are stated more clearly.
5. Original concern: Lack of discussion section.
Assessment of revision:
This concern has been fully addressed. A new discussion section has been added.
6. Original concern: The overall manuscript structure required clearer sectioning, better integration of comparative and mechanistic content, and a more substantive conclusion.
Assessment of revision:
This concern has been fully addressed. The structure of updated manuscript is improved, which makes the paper more

accessible and coherent.

Reviewer #2

(Remarks to the Author)
The authors made appropriate changes according my comments. based on these, I suggest acceptance of the paper.

Reviewer #3

(Remarks to the Author)
The manuscript was improved according to the referee suggestions

Open Access This Peer Review File is licensed under a Creative Commons Attribution 4.0 International License, which permits use, sharing, adaptation, distribution and reproduction in any medium or format, as long as you give appropriate credit to the original author(s) and the source, provide a link to the Creative Commons license, and indicate if changes were made.
In cases where reviewers are anonymous, credit should be given to 'Anonymous Referee' and the source.
The images or other third party material in this Peer Review File are included in the article's Creative Commons license, unless indicated otherwise in a credit line to the material. If material is not included in the article's Creative Commons license and your intended use is not permitted by statutory regulation or exceeds the permitted use, you will need to obtain permission directly from the copyright holder.
To view a copy of this license, visit https://creativecommons.org/licenses/by/4.0/

REVIEWER COMMENTS

Reviewer #1 (Remarks to the Author):

The manuscript presents a structural investigation of heliconical smectic phases in a homologous series of achiral dimers, a combination of polarized optical microscopy (POM), differential scanning calorimetry (DSC), X-ray diffraction (XRD), and resonant soft X-ray scattering (RSoXS), with complementary circular dichroism (CD) and AFM measurements on selected compounds. The identification of a previously unreported SmCTB-C phase and the modeling of double-helical arrangements are notable. However, the manuscript in its current form lacks sufficient interpretive depth, mechanistic analysis, and comparative context. Overall, the work presents promising structural observations, but major revisions are needed to clarify its contributions, strengthen the interpretation, and improve the manuscript's overall coherence.

We thank the referee for the favourable opinion, and we provide a detailed response to specific remarks below.

Below are specific points that should be addressed.

1. The suggestion that a terminal methyl group induces helicity is not supported by references or comparative data. This should either be framed as a hypothesis or substantiated with prior examples.

The sentence that referred to analogous compounds without a terminal methyl group, CT6O.m, was added to the manuscript, together with a reference to the paper describing them (reference: R. Walker, D. Pociecha, J. M. D. Storey, E. Gorecka, C. T. Imrie, Remarkable smectic phase behaviour in odd-membered liquid crystal dimers: The CT6O.m series. J. Mater. Chem. C, 9, 5167, (2021). doi:10.1039/d1tc00904d). For those mesogens, the helical N_{TB} phase was not observed, and the temperature range of smectic phases, which were formed by all studied homologues CT6O.1-CT6O.10, was broader than in the new compounds reported here. It is not that the terminal methyl group induces helicity, but rather that the tendency to form more ordered smectic phases is reduced and consequently the helical N_{TB} phase is revealed.

2. **The reported transitions between 4-layer, 3-layer, and long-pitch heliconical structures are presented descriptively, but the physical basis for these transitions is not explored. There is no discussion of possible steric, elastic, or packing-related mechanisms.**

Unfortunately, there is still no theoretical model that would predict the observed multi-helical structures of smectic phases formed by bent dimers; therefore, our explanation remains speculative.

In many bent-dimer mesogens that had been studied so far, we have observed a transition from a helical smectic structure characterized by a four-layer pitch and a distorted clock-like

molecular arrangement (in which the magnitude of distortion is defined by angle δ being different than 90 deg., see Figure 1 in the manuscript), to a phase exhibiting a double-helix, the pitch of secondary helix being defined by the angle ε. As discussed in our previous work [M. Salamończyk, et al. Multi-level chirality in liquid crystals formed by achiral molecules", Nat. Commun. ***10****:1922, (2019)], a double-helix structure is most probably a 'transient state' on the evolution of the system from a 4-layer structure toward a 2-layer anticlinic smectic phase. The 2-layer anticlinic structure at low temperatures is common for many compounds exhibiting tilted smectic phases. It has also been observed in rod-like systems, where an anticlinic SmC$_A$ phase is formed in the temperature range below the synclinic SmC$_S$ phase. However, while in the systems made of rod-like molecules such evolution is step-like (possibly also with intermediate smectic phases having four- or three-layer repeating units as distinct steps), in the system studied here the evolution is continuous. The evolution between two limiting cases: a 4-layer structure with consecutive synclinic and anticlinic layer interfaces and a 2-layer anticlinic structure, can be obtained by a continuous change of angle ε , defining the pitch of the secondary helix, from 0 to 180 degree. Consequently, the transformation between the four-layer and bilayer structure may occur through a continuous winding of the secondary helix on cooling.*

To clarify these structural aspects, we have added additional explanations to the Introduction and Discussion to better familiarize the reader with the model.

3. **The stabilization of the SmCTB–C phase is not explained. A more mechanistic perspective even if speculative would significantly improve the interpretive value of the work.**

An intercalated structure with a layer spacing d~L/2 is often observed for dimers (bent and linear) in which the length of terminal chains matches the length of an internal spacer; this allows for efficient mixing of terminal chains and internal spacers in alkyl sublayers, well separated from aromatic cores' sublayers. This mechanism was described by e.g. A. F. Alshammari, et al., Soft Matter, ***18,*** *4679-4688, (2022), where it is shown that for some dimers the 'island' of stability of intercalated smectic is achieved if the length of the terminal chain is close to half the spacer length. Extending the spacer moves the 'island' of intercalated smectic stability to longer terminal chains. For the SmC$_{TB-C}$ phase, this intercalation is present along with heliconical structure. A paragraph describing this mechanism and reference was added to the manuscript.*

4. **Although relevant literature is cited, there is little direct comparison with prior studies involving heliconical phases or RSoXS analyses. A more structured discussion of how the present findings differ from existing work would help clarify the contribution.**

The manuscript is not intended to be a review article; therefore, the Introduction presents earlier reports on spontaneous symmetry breaking only briefly and we cited a few publications concerning helical structures formed by achiral molecules. In the revised manuscript we have included additional references to the pioneering resonant X-ray studies: the method was first applied to investigate helical structures of intermediate tilted smectic phases formed by chiral rod-like mesogens [new ref 22,26]. In the supplementary material, an additional reference to

the pioneering work of Dmitrienko was already present. References to RSoXS studies of the N_{TB} phase [29] and smectic phases of bent-core mesogens [27, 28] are also added to the revised manuscript.

Regarding the novelty of the RSoXS measurements reported in the manuscript, what is new is revealing a continuous development from a four-layer to a bilayer structure through the temperature range with stability of nearly perfect-clock three-layer structure. In our previous paper (M. Salamończyk, et al. „Multi-level chirality in liquid crystals formed by achiral molecules", Nat. Commun. ***10****:1922, (2019)) such a structural evolution was predicted but not observed. The materials presented in this manuscript thus confirm the predictions - the secondary helix occurs due to the tendency of the four-layer structure to wind as a way to reach a bilayer anticlinic structure at low temperatures. In current work we also measured the SmCTB-DH evolution in the whole homologue series, including the materials showing direct SmA- SmCTB-DH phase transition.*

5. The manuscript lacks a dedicated Discussion section. Interpretations are embedded within the Results and remain largely descriptive. A standalone section synthesizing the broader significance of the findings would be beneficial.

We moved the interpretation of the results to new Discussion section. A few sentences summarising the importance of the results have been added at the end of the text.

6. The overall structure would benefit from clearer sectioning, improved integration of comparative and mechanistic content, and a more substantive Conclusion. *We followed the reviewer suggestions for a more clear sectioning of the text.*

Reviewer #2 (Remarks to the Author):

The paper describes the chemical synthesis and a thorough physical characterization including birefringence, small-angle and resonant x-ray scattering of a homolog series of flexible bent-shape molecules. Depending on terminal chain length and temperature, it is found that the materials form a twist-bend nematic (NTB) phase and several helical smectic phases labeled as SmCTB–SH, SmCTB–DH and SmCTB–C phase featuring wide-range helical pitches. Based on the abstract, the punchline of the paper appears to be the exceptional diversity of spontaneously chiral phases formed by achiral mesogens. The material synthesis and the physical characterization are professionally and carefully carried out. However, I have several issues with the claim of "exceptional diversity", with the arguments that are likely understandable only for a very narrow group of experts in the field of bent-shape dimers, and consequently with the limited interest the results will attract.

We thank the referee for the favourable opinion, and we hope our responses have clarified all the issues raised by the referee.

1.Spontaneously chiral phases with wide-range of ambidextrous helical pitches are well-known in the B2, B4 and B7 phases of rigid-bent-core molecules (some materials have all these phases). Interestingly the authors only mention the B7 phase that form micron-scale pitch even when the molecule is achiral. They, however, completely ignore the case of the B4 materials that also form helical structures ranging from 0.1-1 μm pitch in form of helical nanofilaments (L. E. Hough et al, Science 2009 , 325 , 452) which can also have a secondary helix (Zhang et al, Nat. Commun.2014 , 5 , 3302) similar to that of the SmCTB–DH mentioned here. Additionally, with variation of the molecular structures, more variants such as modulated helical nanofilament phase (HNFmod) and even helical microfilament phases have been observed by Tsai et al (Angew. Chem. Int.Ed. 2013 , 52 , 5254) and Li et al (Nat. Commun. 2018, 9, 714). Therefore, it looks necessary to me that the authors compare their helical phases of flexible bent-core molecules with the helical B4 phases of rigid bent-core molecules.

This manuscript is not intended as a review article; therefore, the Introduction summarizes only very briefly some earlier studies related to the subject of the manuscript. In the original version, we cited only a limited number of publications related to helical structures formed by achiral molecules. In response to the reviewer's suggestion, we have expanded this section to include additional references related, among others, to the B4 phase [C. Zhang et al Nat Commun 5, 3302 (2014)]. At the same time, we would like to emphasize that the structure of the B4 phase differs substantially from the structure of the SmCTB phases. The B4 phase is structurally closer to a lamellar crystal and the mirror symmetry breaking is associated to its morphology – i.e. formation of twisted fibers. In contrast, the helix observed in the SmCTB phases is related to the helicoidal superstructure known from the chiral SmC-type phases. A sentence related to the superstructure of a B4 phase and related references were added to the text.*

2. The origin of the naming of the SmCTB–SH, SmCTB–DH and SmCTB–C phases is unclear (I assume SH stands for single helix and DH means double helix, but I am unsure what TB-C means) and indicate subtle differences even if they are distinct as the authors claim. The meaning of angles δ and ε introduced in line 198, and the adopted temperature dependences of ε and the tilt angle θ are unclear. I assume if someone were familiar with all the previous papers of the authors on these systems, they were clear, but I think they should be understandable also for people reading only this paper.

The naming of the SmCTB–C phase is based on previous nomenclature for intercalated smectic phases (e.g. the SmAc phase) as described in papers eg. [Attard, G.S.; Date, R.W.; Imrie, C.T.; Luckhurst, G.R.; Roskilly, S.J.; Seddon, J.M.; Taylor, L. Nonsymmetrical dimeric liquid-crystals—The preparation and properties of the alpha-(4-cyanobiphenyl-4'-yloxy)-omega-(4-n-alkylanilinebenzylidene-4'-o xy)alkanes. Liq. Cryst. ***1994****, 16, 529–581] and more recently [Imrie, C.T.; Walker, R.; Storey, J.M.D.; Gorecka, E.; Pociecha, D. Liquid Crystal Dimers and Smectic Phases from the Intercalated to the Twist-Bend. Crystals* ***2022****, 12, 1245.]. We have added a sentence in the manuscript to more clearly explain this naming. According to the reviewer suggestion, we have extended the paragraph in which the details regarding the double helix structure were introduced.*

Square-root temperature dependence of angle ε and the tilt angle θ was estimated from experimental data (RSoXS and optical birefringence, respectively), as explained in detail in Sect. 4 of the Supplementary Material, and taken as a base to model the evolution of unit cell distortion (angle δ). However, we have also checked that other temperature dependence with a proper trend would produce qualitatively similar results. The major point to show was, that the temperature dependence of these angles indeed leads to the disappearance of the $q_4 - q_m$ peak in RSoXS pattern. Major points on the determination of the temperature dependence of angles ε, θ and δ is now added also to the manuscript.

3. Some statements in the conclusions, such as "deformations of the director field, effectively rendering negative values of the bend elastic constant" and "molecular diffusion between adjacent layers (an entropic effect) destabilises this structure and favours tilting of molecules in a single plane" requires more explanation and at least references to be able to follow them.

The concept of a negative bend elastic constant in systems composed of bent-shaped molecules was introduced in the seminal theoretical work by Dozov (Europhys. Lett., 56, 247-253, (2001)). He demonstrated that for negative bend elastic constant the uniform nematic state may lose stability with respect to spatially modulated structures. As a result, the system minimizes its free energy by forming a heliconical structure, which involves bend and twist deformations of director field. Experimental studies have indeed shown that the bend elastic constant in the nematic phase preceding the N_{TB} phase decreases and approaches zero near the transition to the N_{TB} phase (G. Babakhanova et al. Phys Rev E., 96, 062704 (2017)). A negative elastic constant is also believed to be responsible for the formation of heliconical structures in some smectic C-type phases.

The concept of an "in-plane structure" of molecules in the tilted smectic phases was introduced to explain the distorted clock-like structures of SmCFi2 and SmCFi1 phases (see e.g. work by M. Čepič J. Chem. Phys. 22 July 2002; 117 (4): 1817–1826.). The same concept can be applied to smectic phases formed by bent-shaped molecules: the molecules can diffuse more easily between layers (which increases entropy of the system) if they stay in the same tilt plane. In this context, the distorted clock-like structure of SmCTB-SH phase can be explained by two contradictory tendencies. A negative bend elastic constant promotes uniform rotation of director on the tilt cone in successive layers, thus favoring a clock-like model of azimuthal ordering. At the same time, molecular diffusion between layers tends to reduce differences in azimuthal positions of the director on the tilt cone between neighboring layers, thus promoting in-plane ordering. The competition between these two effects results in the formation of the distorted clock-like superstructures of the tilted smectic phases studied here.

4. The description of the birefringence in the Supporting Information is inadequate. From the description "1f and 2f components to yield a retardation induced by the sample. Knowing the sample thickness, the retardation was recalculated into optical birefringence" it is not clear how they measured the birefringence. At least a reference where this technique was described in detail is necessary.

The procedure was described in PEM manual and in 'Polarized Light and Its Interaction With Modulating Devices - a Methodology Review', Hinds International Inc., 1987. This reference was added to SI, and we have also expanded the description of birefringence measurement in the experimental methods section.

In summary, the presented work is likely correct and valid, but the presentation makes it understandable only for a very small number of experts who are completely familiar with the previous work of the authors in this subject. Also, a discussion about similarity and differences of helical systems in achiral rigid bent-core systems seems to be necessary. It is up to the editor to decide whether such presentation is publishable in Nature Communication or not.

We followed the reviewer's suggestions to improve the clarity and accessibility of the manuscript. We hope that with the changes made the manuscript will be more understandable also to readers from outside the liquid crystal field.

Reviewer #3 (Remarks to the Author):

This contribution reports a series of heliconical smectics and nematic liquid crystalline (LC) phases for a homologous series of newly synthesized non-symmetric dimers formed by two different rod-like units interconnected by an odd-numbered hexamethyleneoxy spacer. The synthesis of the compounds is reported in sufficient detail and the required analytical data (1H, 13C-NMR, IR, HR-MS) are provided in the SI.
In the series of compounds, a chain length and temperature dependent transitions between nematic, twist-bend nematic (NTB) and different heliconical tilted smectics LC phases (SmTB) were observed for the synthesized achiral compounds. The Investigation of the LC phases was conducted by POM, DSC and XRD. The structures of the distinct heliconical smectic phases were further investigated by RSoXS, AFM and CD. This confirms the presence of a primary and development of a secondary helix with a complex dependence on molecular structure and temperature (i.e. degree of intercalation, tilt, molecular helicity/conformation). In addition, a reentrant NTB phase was observed at the transition between intercalated and non-intercalated bilayer SmTB phases.

Overall, this is an interesting work with significance for the understanding of spontaneous development of chirality in soft matter systems and providing insights into the relations between different modes of heliconical organization. The work is sound, appropriate research methods have been used and the data are convincingly interpreted, and the conclusions are properly evidenced by experimental data. The paper is considered as appropriate with respect to content and significance for Nat Comm.

We thank the referee for the favourable opinion. We carefully considered all the points given below.

However, the following points need consideration before publication:

p.2, line ~80: the methyl group is also expected to have an influence on the molecular conformation of the Schiff base unit, thus favoring helical conformations and supporting helix formation.

Although we cannot exclude that specific molecular conformations may favour the formation of helical structures (such a feedback effect was suggested as a possible mechanism for mirror symmetry breaking in the isotropic liquid phase by C. Tschierske), we currently do not have experimental evidence that would allow us to unambiguously confirm this hypothesis.

P. 2, line89: I would suggest to show the local structure in N/NTB (intercalated with d = L) in the SI for more clarity (similar to Fig. 1e,f).

There is probably some misunderstanding – 'intercalated' refers to a structure with d~L/2, where L is the full length of dimeric molecules, which is presented in Figure 1e. We would like to stress that this figure shows schematically local molecular packing arrangement, which is present not only in SmCTB-C phase but also in N and NTB phases of short homologues.

p.3 (Fig. 1f) why is structure (f) assigned as partly interdigitated bilayer, at least in the shown figure there is only a minimal overlapping between the CH3 groups at the ends (if the figure would be in scale, no numerical molecular parameters are given). In this case the small difference could easily be explained by conformational disorder and the orientational order parameter being <1 in a (non-intercalated) bilayer structure? (also the tilt contributes to the slight reduction of d). For a "partly interdigitated bilayer phase" ("SmXd") a much lager difference between d and 2L of around d = 0 1.4-1.8 Lmol is usually found. A graphic or table comparing molecular lengths with d-values or d/L values in the SI would be helpful. It would also be easier for the reader if an intercalated and a non-intercalated bilayer smectics phases would be distinguished.

The difficulty in the case of bent-shape dimers is that the molecular length is not easily defined based on molecular structure simulations. Even small changes in the conformation of the spacer can lead to significant variations in the bending angle between mesogenic units, and consequently in the effective molecular length defined as the distance between molecular ends. Indeed, the conformation may also change with temperature, and this effect becomes more pronounced with increasing length of the spacer between mesogenic units or terminal chains. This makes it difficult to precisely define the relationship between d and L. Because the exact ratio d/L is not known, we decided to avoid using the term "partially interdigitated". Instead, for simplicity, we refer to this phase as a bilayer structure, since the layer spacing d is approximately twice the molecular length (L defined for all trans conformation of the spacer).

p.3 line 91: Why is the intercalated bilayer structure named SmCTB-C? what is the meaning of "C" here? Why not, for example, SmCTB-1/2 ?

The naming of the SmCTB–C phase is based on previous nomenclature for intercalated smectic phases (e.g. the SmAc phase) as described in papers eg. [Attard, G.S.; Date, R.W.; Imrie, C.T.;

Luckhurst, G.R.; Roskilly, S.J.; Seddon, J.M.; Taylor, L. Nonsymmetrical dimeric liquid-crystals—The preparation and properties of the alpha-(4-cyanobiphenyl-4'-yloxy)-omega-(4-n-alkylanilinebenzylidene-4'-o xy)alkanes. Liq. Cryst. ***1994****, 16, 529–581] and more recently [Imrie, C.T.; Walker, R.; Storey, J.M.D.; Gorecka, E.; Pociecha, D. Liquid Crystal Dimers and Smectic Phases from the Intercalated to the Twist-Bend. Crystals* ***2022****, 12, 1245.]. We have added a sentence in the manuscript to more clearly explain this naming.*

p. 5, Fig. 3a: there is no visible change of d at the SmA-SmC transition which would suggest that SmA is in fact at tilt-randomized SmC phase (similar winding/unwinding process as at the N-NTB transition). Could this be commented?

In the case of bent-shaped dimers, the temperature dependence of the layer spacing below the SmA–SmC phase transition, might be more complex than for rod-like materials as it results from several competing effects, including the increasing molecular tilt, changes in molecular length arising from conformational variations, and the intercalation of alkyl chains between the layers. Consequently, the decrease of the layer spacing d may not be very pronounced, particularly for homologues with long spacers between the mesogenic cores and long terminal chains. The additional information was included in the text.

p. 5: d0 is the single molecular layer (= d1/2), it would be helpful to also indicate this distance d0 in Fig 1f.

The definition of d_o as single layer thickness was added to Fig 1b.

In order to make this paper more accessible to readers from a broader field of mirror symmetry breaking and helical self-assembly, I would suggest to add at the end of the conclusion one or two sentence outlining the importance of the obtained results with a broader view.

The materials presented in this manuscript confirm the predictions made in Nat. Commun. 9, 228 (2018), the secondary helix occurs due to the tendency of the four-layer structure to wind and form anticlinic bilayer structure at low temperatures. The materials studied here exhibit a continuous development from a four-layer to a bilayer phase through the changes of the secondary helix pitch length. Because the helical structure determines the reflection and transmission of light with certain polarization and wavelength, the presented findings are also important for photonic engineering.

Moreover, in the introduction: On p2 lines 51ff, during the discussion of chirality by achiral molecules in fluid systems, it should be noted that even in Isotropic liquids spontaneous chirality/helicity, (though in this case with helical instead of heliconical structure) has been observed (Dressel et al. N Nat. Commun. 9, 228 (2018).at. Chem. 2014, 6, 971). Moreover, it should be pointed out (lines 57/58) that the first example of heliconical smectic phases of achiral molecules (in this case a polar version of SmTB with incommensurate 4-layer structure) was discovered for achiral bent-core mesogens (Sreenilayam et al. Nat Commun. 2016, 7, 11369). This work has appeared 2 years before the authors own work with dimesogens (ref. 13).

The suggested reference was added.

SI: The original references for the used synthesis procedures and the sources of the starting materials should be given. Are 6-bromo-1-[4-(4-bromophenyl)phenyl]hexan-1-one, 1-bromo-4-[4-(6-bromohexyl)phenyl]benzene, 4-[4-[4-(6-bromohexyl)phenyl]phenyl]benzonitrile, new compounds? Otherwise please give literature refences to previous synthesis. Please also add original 1H and 13C NMR spectra for at least one representative of the final compounds.

The listed intermediates are not new compounds, literature reference has been provided.

Sample NMR spectra have been included for compound: CT6O2Me.1.

REVIEWERS' COMMENTS

Reviewer #1 (Remarks to the Author):

The revised manuscript is clearer, better structured, and substantially improved compared with the original submission. The authors now provide a more careful hypothesis rather than an overly speculative mechanistic interpretation, making the overall argument more coherent and convincing. In particular, the additional mechanistic discussion, the expanded comparison with prior studies, and the inclusion of a dedicated Discussion section have strengthened the paper. Most of my original concerns have been addressed. Although direct evidence that the mechanism of the evolution of four-layer distorted clock-like heliconical arrangement toward eventually anticlinic bilayer structure is still lacking, the revised manuscript presents a more convincing mechanistic framework in which the chiral structure is interpreted as arising from the competition between negative bend elasticity, which favors heliconical ordering, and steric-entropic interlayer diffusion effects, which favor in-plane ordering. The added references sufficiently support this general framework, although they do not uniquely establish the specific structural evolution proposed for the present system. With this remaining concern in mind, obtaining more direct evidence to substantiate the proposed mechanism would be an important direction for future work.

We thank the Reviewer for their comments on the revised manuscript, and their positive assessment of the revisions made.

Point-to-point assessment is as follows:

1. Original concern: The suggestion that a terminal methyl group induces helicity was not sufficiently supported by references or comparative data.

   Assessment of revision:
   This point has been addressed satisfactorily. The authors have now introduced a comparison with the analogous CT6O.m series and clarified that the role of the methyl substituent is not to directly “induce helicity,” but rather to reduce the tendency to form more highly ordered smectic phases, thereby allowing the heliconical NTB phase to emerge more readily. The logical chain here is more defensible than original claim.

2. Original concern: The manuscript described these transitions phenomenologically, but did not discuss possible steric, elastic, or packing-related mechanisms.

   Assessment of revision:
   The author acknowledges the niche of the theoretical model for these multi-helical smectic structures, and the explanation remains speculative. This clarification is appreciated and current interpretation is literature supported by added references. However, the proposed pathway from a distorted four-layer structure toward a ultimately toward a bilayer anticline tendency is still not directly demonstrated, which could be the future direction for authors to explore more. This concern has been partially resolved. The mechanism discussion is improved and acceptable, but it could be stronger if more direct evidence could be provided.

3. Original concern: The stabilization of the SmCTB-C phase was not explained, and even a speculative mechanistic interpretation would improve the paper.

   Assessment of revision:
   This point has been addressed well in current version.

4. Original concern: The original manuscript did not sufficiently position the present work relative to prior studies on heliconical phases and resonant soft X-ray scattering.

   Assessment of revision: This concern has been addressed sufficiently. The revised version added relevant reference to earlier RSoXS work on helical structures in rod-like mesogens, NTB phase, and bent-core smectic systems, the novelty of present work are stated more clearly.

5. Original concern: Lack of discussion section.

   Assessment of revision:
   This concern has been fully addressed. A new discussion section has been added.

6. Original concern: The overall manuscript structure required clearer sectioning, better integration of comparative and mechanistic content, and a more substantive conclusion.

   Assessment of revision:
   This concern has been fully addressed. The structure of updated manuscript is improved, which makes the paper more accessible and coherent.

Reviewer #2 (Remarks to the Author):
The authors made appropriate changes according my comments. based on these, I suggest acceptance of the paper.

We thank the reviewer for their positive response.

Reviewer #3 (Remarks to the Author):
The manuscript was improved according to the referee suggestions.

We thank the reviewer for their positive assessment of our revision.
